# Model-guided chemical environment and metabolic network design to couple pathways with cell fitness

**DOI:** 10.1016/j.mec.2025.e00267

**Published:** 2025-11-22

**Authors:** Natalia Kakko von Koch, Tuula Tenkanen, Sandra Castillo, Virve Vidgren, Tino Koponen, Kristoffer Krogerus, Merja Penttilä, Paula Jouhten

**Affiliations:** a VTT Technical Research Centre of Finland Ltd, Espoo, Finland; b Aalto University, School of Chemical Engineering, Department of Bioproducts and Biosystems, Espoo, Finland

**Keywords:** Adaptive laboratory evolution, Genome-scale metabolic model, Glycolic acid, Genetic algorithm

## Abstract

Heterologous compound production is a complex trait since the native metabolic fluxes supplying the precursors, redox power, and energy are under multilevel cellular regulation. Improving complex traits using targeted engineering needs combinatorially charting the complex genetic underpinnings. While this is laborious, adaptive laboratory evolution (ALE) has been used to improve many traits of microbial strains that are of application relevance such as tolerance of harsh conditions and nutrient utilization. However, in contrast to such traits, heterologous production can seldom be intuitively coupled with cellular fitness.

Here, a novel method EvolveXGA was developed for genome-scale metabolic model guided design of strategies combining chemical environments and genetic engineering of the metabolic network to allow ALE of desired traits. Adaptive evolution of traits occurs when the co-variance between the traits and fitness involves a genetic dependency like a flux coupling would indicate. Thus, combinations of chemical environments and metabolic network structures were searched using a genetic algorithm to identify those that render desired traits (i.e., sets of metabolic fluxes) flux-coupled with fitness. The search was performed for the production of 29 heterologous compounds in yeast *Saccharomyces cerevisiae*. Strategies for coupling the production routes of 13 compounds with fitness were found with four metabolic reaction knock outs and three components in the chemical environment. In addition, strategies for fitness-coupling native fluxes involved in the production was found for the remaining compounds. In addition, a model-guided strategy was implemented for fitness-coupling of heterologous glycolic acid (GA) synthesis in *S*. *cerevisiae* via oxaloacetase, oxalyl-CoA synthetase, and oxalyl-CoA reductase (i.e., oxalate pathway). ALE was performed and evolved populations and isolated clones were characterized using whole-genome sequencing and quantitative metabolite analysis. Three out of six isolates had better GA yield from glucose than a non-optimized control strain expressing the oxalate pathway and glyoxylate reductase.

EvolveXGA generalizes metabolic model-guided design of strategies to couple production routes with cell fitness. The strategies bring optimizing heterologous production in engineered microbial cells in the realm of ALE. Slow and expensive strain optimization is a major hinder of novel processes using engineered microbial cells reaching industrial realization. Thus, EvolveXGA contributes to biotechnological solutions for the brighter future.

## Highlights

• EvolveXGA is a novel method for genome-scale metabolic model guided design of strategies combining chemical environments and genetic engineering of the metabolic network to allow adaptive laboratory evolution (ALE) of desired traits.
• Strategies were found for using ALE to enhance the production of 29 heterologous compounds in yeast *Saccharomyces cerevisiae.*
• A model-guided strategy was demonstrated for Darwinian selection of a heterologous route for glycolic acid synthesis in *S. cerevisiae*.

## Graphical abstract

**Figure:**
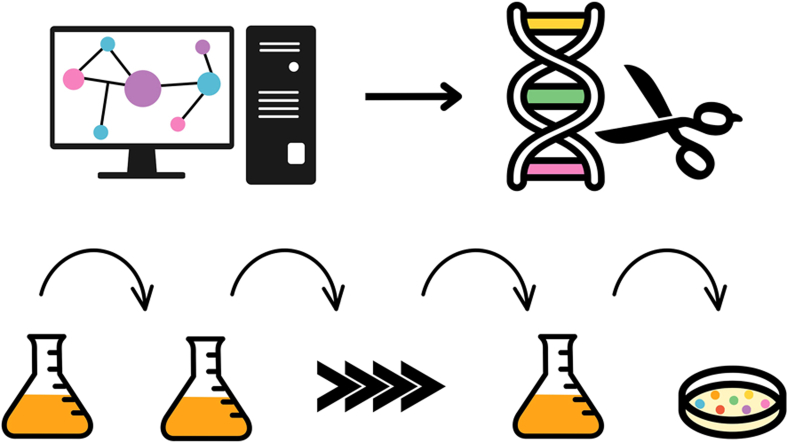

## 1 Introduction

Adaptive laboratory evolution (ALE) has been used to improve numerous invariably fitness-associated traits of microbial strains such as tolerance to high temperatures, low pH, and utilization of non-natural nutrients (Lee and Palsson Bernhard, 2010; Liu et al., 2025; Oide et al., 2015). Trait evolution in ALE occurs when there is covariance with genetic underpinnings between the trait and fitness (Price, 1970; Rausher, 1992; Robertson, 1966, 1968). However, the trait-fitness dependency may be conditional. Previously, we took advantage of the genetic underpinnings of chemical environment dependent metabolic flux couplings (Burgard et al., 2004) to perform genome-scale metabolic model (GEM) guided design of suitable chemical environments for improving specific, not necessary fitness increasing, metabolic traits using ALE (Jouhten et al., 2022). Model-guided ALE was demonstrated for non-GMO strain improvement. However, the major advantage of ALE in strain improvement, i.e., the ability to circumvent the need to know the genetic underpinnings of the desired traits, is extremely valid also when genetic engineering can be used. Optimizing the central metabolic fluxes for providing precursors, redox and energy for heterologous production is challenging due to complex, multi-level regulation of metabolic pathway activities (Cakar et al., 2012; Lee and Kim, 2020; Nielsen, 2017). Intuitive ALE strategies for improving heterologous production are limited. Most heterologous production does not benefit the fitness of cells but rather withdraws resources and causes burden to cells (Raman et al., 2014; Wehrs et al., 2019; Wu et al., 2016). A rare example of ALE use for improving heterologous product yield is an enhancement of beta-carotene synthesis under intuitively set up oxidative conditions (Reyes et al., 2014).

More commonly heterologous production strains are optimized using targeted genetic engineering of the metabolic network. GEM-guided methods for metabolic network optimization fall into two major categories. They either aim at pushing and pulling precursors towards product synthesis (Li et al., 2016) or creating growth-product coupling (Burgard et al., 2003; Klamt et al., 2025; Li et al., 2024; Patil et al., 2005; Tepper and Shlomi, 2010). Growth-product coupling strategies modify the metabolic network in a way that over-production may occur or has to occur in order for the cells to synthesize all essential biomass components (Burgard et al., 2003; Tepper and Shlomi, 2010). *In silico* growth-product coupling strategies can be found for a wide range of compounds, like all *Escherichia coli* metabolites (Klamt and Mahadevan, 2015). Unfortunately, implementing the strategies may involve such high numbers of metabolic gene deletions that the hosts would unlikely be viable (Klamt and Mahadevan, 2015; von Kamp and Klamt, 2017). However, the network engineering strategies depend on the chemical growth environment of the cells, which is scarcely exploited so far (Agrawal and Stinchcombe, 2009).

Here an algorithm EvolveXGA was developed to generalize the GEM-guided strain design for coupling production traits with cellular fitness. EvolveXGA is implemented as a genetic algorithm similarly to OptGene algorithm (Patil et al., 2005), but instead of considering only gene deletions, it combines metabolic network engineering with the selection of chemical environments to allow using ALE for enhancing native and heterologous pathways involved in production. The generalized design was demonstrated *in silico* for identifying metabolic reaction knock outs and evolution environments suitable for coupling traits of producing 29 heterologous compounds in yeast *S. cerevisiae*. The design for glycolic acid (GA) production using a heterologous oxalate pathway (Toivari et al., 2020) was implemented, the experimental ALE performed and with minimal screening strains producing GA from glucose with near wild type growth could be isolated and characterized.

## 2 Results

### 2.1 Chemical environment and metabolic network couple synthetic metabolic routes to cell growth

**Fig. 1:**
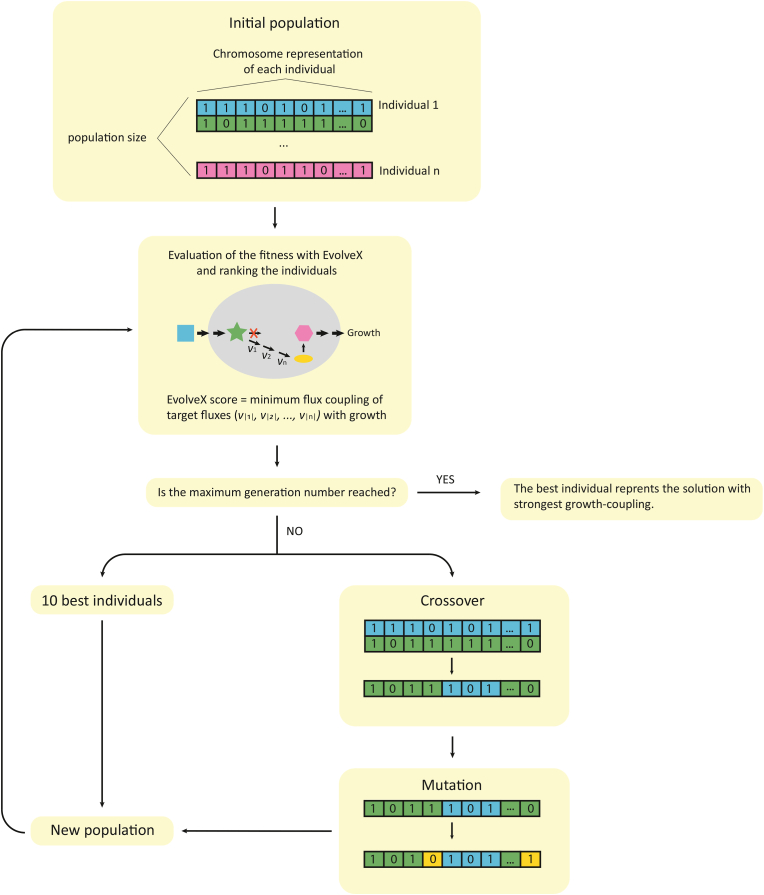
Schematic figure of EvolveXGA algorithm.

Metabolic network structure and the chemical environment of the cells determine which metabolic fluxes are quantitatively dependent on each other during steady-state metabolism (i.e., are flux-coupled) (Burgard et al., 2004). Previously the dependency of the flux couplings on the chemical environment was used to score chemical environments by the sum of GEM simulated selected flux couplings to growth (Jouhten et al., 2022). The stronger the coupling of flux to growth (i.e., proxy of fitness) the stronger the expected Darwinian selection on the flux in the particular chemical environment. Since the flux coupling indicates genetic dependency (Notebaart et al., 2008), adaptive evolution of metabolic traits (i.e., defined as sets of metabolic fluxes) is expected and determining flux couplings could be used to score chemical environments by their suitability for adaptively evolving selected fluxes (Jouhten et al., 2022). Here, this previously developed scoring algorithm EvolveX (Jouhten et al., 2022) was extended from only chemical environments to metabolic network modifications (i.e., deletions, synthetic pathways). The scoring algorithm was introduced into an optimization routine, a genetic algorithm (EvolveXGA) (Fig. 1) that uses a GEM to find such a chemical environment and reaction deletions (achievable with gene deletions) that selected fluxes become coupled with growth (i.e., proxy of fitness) as strongly as possible.

As optimization in genetic algorithms mimic Darwinian selection, the terminology of genetic algorithms also originates from evolution. EvolveXGA is initialized by forming the first “population” of individuals. An individual consists of a chromosome that represents one solution. Each metabolic flux and chemical component in the EvolveXGA search space is represented with a binary value in the chromosome. The binary value 0 indicates reaction knock outs and components in the chemical environment. After generating the initial population each individual is scored with the EvolveX algorithm (Jouhten et al., 2022). EvolveX calculates the sum of minimum flux couplings with growth of up-regulation flux targets and maximum flux couplings with growth of down-regulation flux targets. Thus, the score represents a combined flux coupling of selected fluxes with fitness that is specific for an individual of the genetic algorithm, thus, metabolic network structure and chemical environment. After scoring each individual a new population is generated using elitism, crossover and mutation. The ten best individuals from the previous generation are selected to the new population (i.e., elitism). Then the rest of the new population is filled performing crossover and mutation. In crossover new combinations of two randomly selected individuals (“parents”) are formed. The offspring is then mutated by randomly changing one reaction knock out or chemical component. The optimization is continued until a predefined number of generations is reached.

**Fig. 2:**
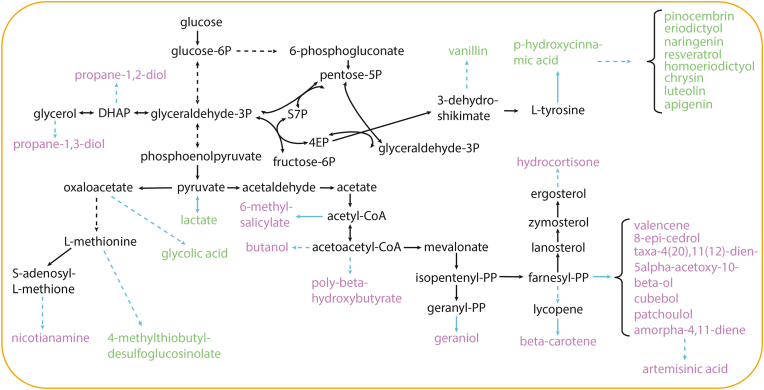
Heterologous compounds for which strategies to couple the production pathways with cells' fitness were searched. Strategies with four reaction knockouts and three components in the chemical environment were found for coupling the heterologous production pathways of 13 compounds (green) with fitness. Strategies for fitness coupling of native fluxes involved in the production were additionally found for the remaining 16 compounds (purple). Only relevant native metabolites, pathways and reactions (black) are shown. The heterologous pathways are indicated in blue.

First, the EvolveXGA algorithm was evaluated *in silico* by searching strategies for Darwinian selection of routes to 28 heterologous compounds synthesized from precursors distributed in central metabolism (Fig. 2) (Jouhten et al., 2016). The routes (Supplementary material) were added to *S. cerevisiae* GEM and the search space was limited in potential reaction knock outs and components of chemical environment (Supplementary material). Each individual in the initial population was randomly assigned with four reaction knock outs. In addition, three chemical components were randomly selected to the initial chemical environment. The number of reaction knock outs and chemical components was kept constant during the optimization. Both crossovers and mutations were performed with 80 % probability separately for reaction knock outs and chemical components.

Strategies for coupling fluxes of heterologous production pathways with fitness combining reaction knock outs (achievable with gene deletions) and chemical environments were found for twelve heterologous production pathways including pathways to 4-methylthiobutyl-desulfoglucosinolate, apigenin, chrysin, eriodictyol, homoeriodictyol, lactate, luteolin, naringenin, p-hydroxycinnamic acid, pinocembrin, resveratrol and vanillin (Fig. 2, Supplementary material). The optimization was continued for 5000 generations, but the optimal score was found in all simulations before 1000 generations.

**Fig. 3:**
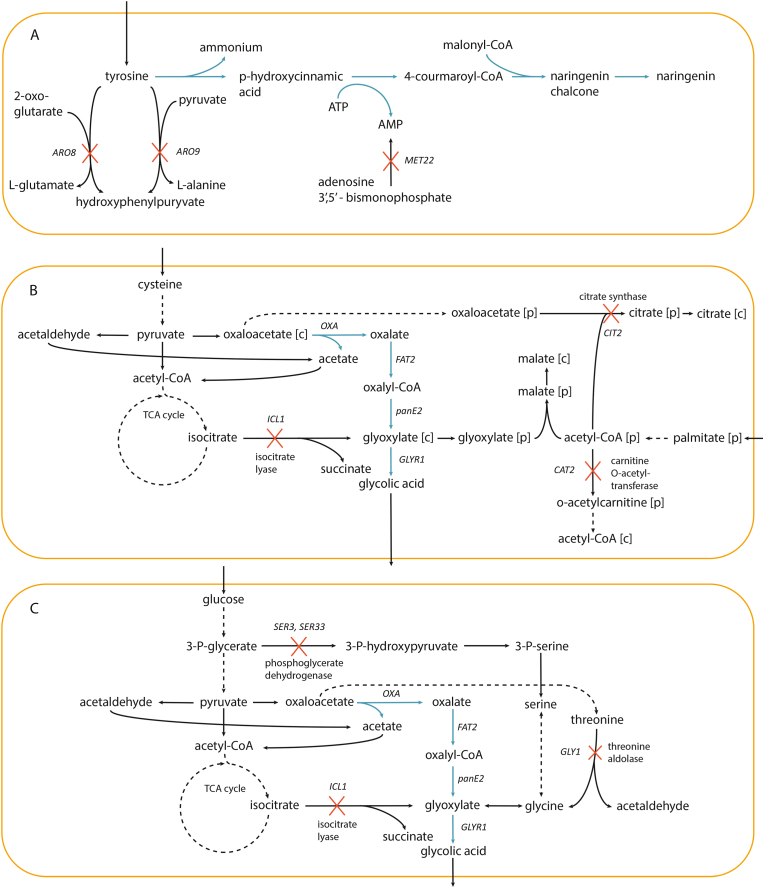
Strategies combining genetic engineering and chemical environments for coupling pathways with growth. Strategies for fitness coupling of naringenin production route (a), glycolic acid production route (b), and glycolic acid production route when glucose and ammonia are used in ALE chemical environment as sole carbon and nitrogen sources (c). In a-c only relevant reactions are shown and cofactors participating in the reactions are omitted. The heterologous pathways are presented in blue and native metabolic reactions in black. Suggested reaction knock outs are marked with red crosses.

Solutions for coupling apigenin, chrysin, eriodictyol, homoeriodictyol, luteolin, naringenin, pinocembrin and resveratrol production routes with cell fitness had L-tyrosine, glycerol and palmitate in the chemical environment. Furthermore, the solution for p-hydroxycinnamic acid production route had L-tyrosine and palmitate in the chemical environment. L-tyrosine was found in the chemical environment as all the routes start with L-tyrosine ammonia lyase deaminating L-tyrosine to the product precursor p-hydroxycinnamic acid (Fig. 3a). However, the GEM lacked the Ehrlich pathway reactions for tyrosine catabolism (Hazelwood et al., 2008). Therefore, if these solutions were implemented, *ARO8* and *ARO9* would have to be deleted. *ARO8* and *ARO9* encode aromatic amino acid aminotransferases catalyzing tyrosine + 2-oxoglutarate - > L-glutamate + hydroxyphenylpyruvate and tyrosine + pyruvate - > L-alanine + hydroxyphenylpyruvate, respectively (Fig. 3a). The chemical environment was determining for the coupling of naringenin synthesis with cell fitness. The EvolveX score decreased only by 5 % when reaction deletions were not considered. Only 3′,5′-bisphosphate nucleotidase knock out contributed notably to the score (other deletions increased the score only by 0.03 %). 3′,5′-bisphosphate nucleotidase generates AMP to be used e.g., in RNA biosynthesis. If it was knocked out, AMP generated in naringenin production route would contribute to RNA synthesis, and thus, the fitness of cells.

### 2.2 Darwinian selection can be extended to native reactions providing precursors, energy and redox power for heterologous production

Since heterologous synthesis pathways of all compounds could not be coupled with fitness with up to four of reaction knock outs, we next searched for strategies to couple the changes in native metabolism needed for enhancing the production with fitness improvement. The minimum set of fluxes that need to change when objective changes from growth (i.e., proxy of wild type) to production was determined and fluxes that needed to increase were extracted. These native fluxes were used as targets in EvolveXGA algorithm. Strategies combining ALE chemical environments and metabolic reaction deletions were found for the native flux target sets of all remaining 16 heterologous compounds investigated (Fig. 2, Supplementary material).

Strategies for enhancing the production of ten compounds (6-methylsalicylate, 8-epi-cedrol, amorpha-4-11-diene, artemisinic acid, cubebol, geraniol, hydrocortisone, patchoulol, taxa-4(20),11(12)-dien-5alpha-acetoxy-10beta-ol and valencene) by Darwinian selection of the native fluxes involved an evolution environment containing 2-oxoglutarate and L-methionine and had equal or related reaction knock outs. The strategies included a knock out of malate synthase and a knock out of glucose 6-phosphate dehydrogenase, 6-phosphogluconolactonase or phosphogluconate dehydrogenase that form a linear oxidative branch of pentose phosphate pathway. Knocking out the oxidative pentose phosphate pathway, a major source of NADPH, targets selection to other NADPH generating reactions including acetaldehyde dehydrogenase (i.e., Ald6) involved in the route to cytosolic acetyl-CoA, which is a common precursor for all of the compounds. In addition, if malate dehydrogenase is knocked out, acetyl-CoA cannot be lost to malate. Thus, ALE could be used to develop chassis strains suitable for the production of several heterologous compounds.

### 2.3 Model-guided synthetic pathway coupling with cell fitness to enhance glycolic acid precursor formation using ALE

To further validate the EvolveXGA algorithm a Darwinian selection strategy for the GA synthesis pathway (i.e., oxalate pathway (Toivari et al., 2020), Supplementary material) was searched. GA production has been demonstrated in several hosts like yeasts and *E. coli* (Deng et al., 2018; Koivistoinen et al., 2013; Zahoor et al., 2014). The production levels of GA in *S. cerevisiae* have remained low, despite it having many benefits over non-conventional yeasts, such as fast glucose utilization. The GEM of *S. cerevisiae* was augmented with the first three reactions of the synthetic oxalate pathway (i.e., oxaloacetase, oxalyl-CoA synthetase, and oxalyl-CoA reductase) and strategies for the Darwinian selection of the pathway fluxes were searched by running EvolveXGA for 3000 generations. The strategy consisting of a specific chemical environment and reaction knock outs with the highest EvolveX score representing the highest sum of the pathway reactions’ flux couplings with growth involved a chemical environment with L-methionine, pyruvate and spermidine and knock outs of 6-phosphogluconolactonase, formate-tetrahydrofolate ligase, pyruvate decarboxylase and pyruvate dehydrogenase (Table 1, Solution A). However, the predicted total nutrient utilization per biomass was very high (i.e., pyruvate utilization was 1714 mmol/g CDW and L-methionine 19 mmol/g CDW) indicating of very inefficient growth likely leading to viability issues. Therefore, fitness-coupling solutions were searched by restricting the total nutrient utilization to a maximum of 75 mmol/g CDW. The solution with highest score (Table 1, Solution B) had acetate, palmitate and L-cysteine in the chemical environment and knock outs of peroxisomal carnitine O-acetyltransferase, peroxisomal catalase, peroxisomal citrate synthase and isocitrate lyase (Fig. 3b). If peroxisomal citrate synthase, peroxisomal carnitine-O-acetyltransferase and isocitrate lyase were deleted the cells would have to get glyoxylate from oxalate route to utilize acetyl-CoA derived from palmitate for growth.

**Table 1:** Strategies for fitness coupling the flux of oxalate pathway to glycolic acid synthesis in *S. cerevisiae*. Solutions A-B combined reaction knock outs with chemical environments and in solutions C-D glucose and ammonium as the chemical environment was considered.

Solution A			
**EvolveX score**	5060.78 mmol/g CDW		
	**compound name**		
**Evolution environment**	methionine		
	pyruvate		
	spermidine		
**Deleted reactions**	**reaction name**	**reaction**	**gene(s) encoding for the enzyme(s) catalyzing** **the reaction**
	6-phosphogluconolactonase	6-O-phosphono-D-glucono-1,5-lactone + H2O → 6-phospho-D-gluconate + H+	*SOL3*, *SOL4*
	formate-tetrahydrofolate ligase	ATP + formate + THF ≤> 10-formyl-THF + ADP + phosphate	*MIS1*
	pyruvate decarboxylase	H+ + pyruvate → acetaldehyde + carbon dioxide	*PDC1*, *PDC5*, *PDC6*
	pyruvate dehydrogenase	coenzyme A + NAD + pyruvate → acetyl-CoA + carbon dioxide + NADH	*PDB1*, *PDA1*, *LPD1*, *PDX1*, *LAT1*

**Solution B**			
**EvolveX score**	415.85 mmol/g CDW		
	**compound name**		
**Evolution environment**	acetate		
	cysteine		
	palmitate		
	**reaction name**	**reaction**	**gene(s) encoding for the enzyme(s) catalyzing** **the reaction**
	carnitine O-acetyltransferase	(R)-carnitine + acetyl-CoA → coenzyme A + O-acetylcarnitine	*CAT2*
**Deleted reactions**	catalase	2.0 hydrogen peroxide → 2.0 H2O + oxygen	*CTA1*
	citrate synthase, peroxisomal	acetyl-CoA + H2O + oxaloacetate → citrate + coenzyme A + H+	*CIT2*
	isocitrate lyase	isocitrate → glyoxylate + succinate	*ICL1*

**Solution C**			
**EvolveX score**	11.55 mmol/g CDW		
**Deleted reactions**	**reaction name**	**reaction**	**gene(s) encoding for the enzyme(s) catalyzing** **the reaction**
	acetyl-CoA synthetase	acetate + ATP + coenzyme A → acetyl-CoA + AMP + diphosphate	*ACS1*
	isocitrate dehydrogenase (NAD+)	isocitrate + NAD → 2-oxoglutarate + carbon dioxide + NADH	*IDH1*, *IDH2*
	pyruvate decarboxylase	H+ + pyruvate → acetaldehyde + carbon dioxide	*PDC1*, *PDC5*, *PDC6*
	pyruvate dehydrogenase	coenzyme A + NAD + pyruvate → acetyl-CoA + carbon dioxide + NADH	*PDB1*, *PDA1*, *LPD1*, *PDX1*, *LAT1*

**Solution D**			
**EvolveX score**	5.08 mmol/g CDW		
**Deleted reactions**	**reaction name**	**reaction**	**gene(s) encoding for the enzyme(s) catalyzing** **the reaction**
	3′,5′-bisphosphate nucleotidase	adenosine 3′,5′-bismonophosphate + H2O → AMP + phosphate	*MET22*
	isocitrate lyase	isocitrate → glyoxylate + succinate	*ICL1*
	phosphoglycerate dehydrogenase	3-phosphonato-D-glycerate (3-) + NAD → 3-phospho-hydroxypyruvate + H+ + NADH	*SER3*, *SER33*
	threonine aldolase	L-threonine → acetaldehyde + L-glycine	*GLY1*

Finally, we evaluated if EvolveXGA can find Darwinian selection strategies for the oxalate pathway when only metabolic network modifications with glucose and ammonia as sole carbon and nitrogen sources are considered, respectively. EvolveXGA was run ten times for 1000 generations and always converged to two different reaction knock out solutions (Table 1, Solutions C and D). The highest scoring solution with glucose and ammonium as the sole carbon and nitrogen sources (Solution C) suggested knocking out pyruvate decarboxylase and pyruvate dehydrogenase. Since both of these enzymes play central roles in energy and central carbon metabolism, their knock out was not appealing though theoretically feasible. The other solution (Solution D) suggested knock outs of 3′,5′-bisphosphate nucleotidase, isocitrate lyase, phosphoglycerate dehydrogenase and threonine aldolase. Without the 3′,5′-bisphosphate nucleotidase knock out the score was reduced only 12 %. Thus, the oxalate pathway could be exposed to Darwinian selection on glucose and ammonium medium by deleting genes encoding isocitrate lyase (*ICL1*), phosphoglycerate dehydrogenase (*SER3*, *SER33*) and threonine aldolase (*GLY1*).

### 2.4 Replacement of the glycolytic route to L-serine and glycine synthesis with the synthetic oxalate pathway coupled glycolic acid precursor formation to growth

**Fig. 4:**
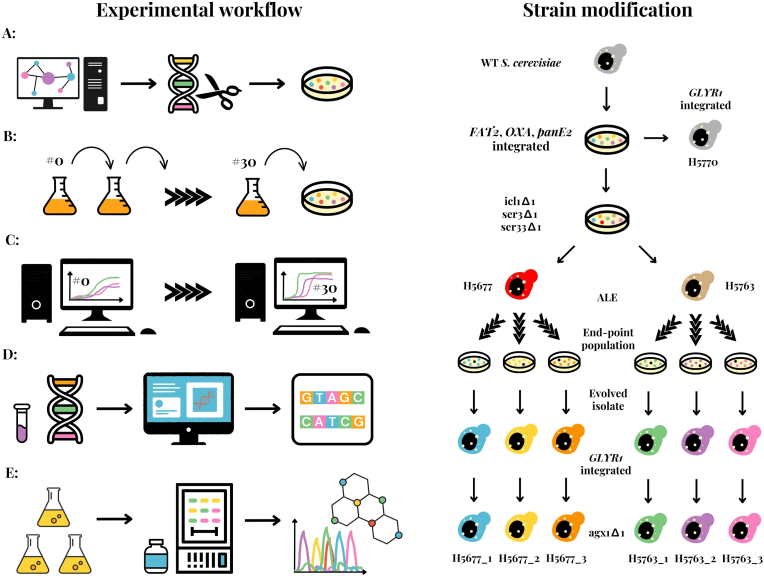
Experimental workflow and strain manipulations. Implementation of the strategy for coupling glycolic acid production route with fitness and subsequent strain improvement using adaptive laboratory evolution (on the left). Metabolic network modifications were introduced following a model-guided strategy designed using EvolveXGA algorithm (a). According to the model-guided strategy the adaptive laboratory evolution conditions were set up for evolving the modified strains in three replicate lineages (b). Quantitative characterization of the growth of evolved populations and isolated clones, whole-genome sequencing (c) and quantitative metabolite analyses (d) were performed. Strain manipulations including all gene deletions and integrations performed (on the right). Wild type (WT) *S. cerevisiae* CEN.PK 113-7D had the oxalate pathway except GLYR1 integrated. GLYR1 was integrated into the control strain H5770, while *ICL1*, *SER3* and *SER33* were deleted from the parental strains H5677 and H5763. After ALE of six independent lineages started from the two parental strains, GLYR1 was introduced into one evolved isolate from each lineage. Additional strains having *AGX1* deleted were also engineered.

To validate the model-guided strategy for enhancing GA production using ALE (Fig. 3c), *ICL1*, *SER3* and *SER33* were deleted from the wild type *S. cerevisiae* CEN.PK113-7D (H3887) (Fig. 4). The deletions of *SER3* and *SER33* disabled the glycolytic route to L-serine and glycine synthesis. When combined with *ICL1* deletion, the remaining routes for L-serine and glycine synthesis were either via the synthetic oxalate pathway (Toivari et al., 2020) or via threonine aldolase Gly1. Threonine aldolase encoding *GLY1* was not deleted because has a ΔG close to zero (Flamholz et al., 2012), substrate and product concentrations are similar in *S. cerevisiae* (Stanley and Pamment, 1993), and therefore the net flux per enzyme abundance is expected to be low (Noor et al., 2014). Furthermore, increased threonine aldolase flux would direct resources towards oxaloacetate, the precursor of our synthetic oxalate pathway. The synthetic oxalate pathway was introduced into *S. cerevisiae* CEN.PK113-7D (H3887) (*icl1*Δ*1 ser3*Δ*1* s*er33*Δ*1)* as *FAT2*, *OXA* and *panE2* genes (Fig. 3c). In the first step of the oxalate pathway, oxaloacetase, encoded by *OXA*, converts oxaloacetate to oxalate and acetate. Next, oxalyl-CoA synthetase, encoded by *FAT2*, converts oxalate to oxalyl-CoA. Finally, oxalyl-CoA reductase, encoded by *panE2*, converts oxalyl-CoA to glyoxylate, a precursor of glycine, L-serine, and GA.

### 2.5 Near wild type growth restored during adaptive laboratory evolution

**Fig. 5:**
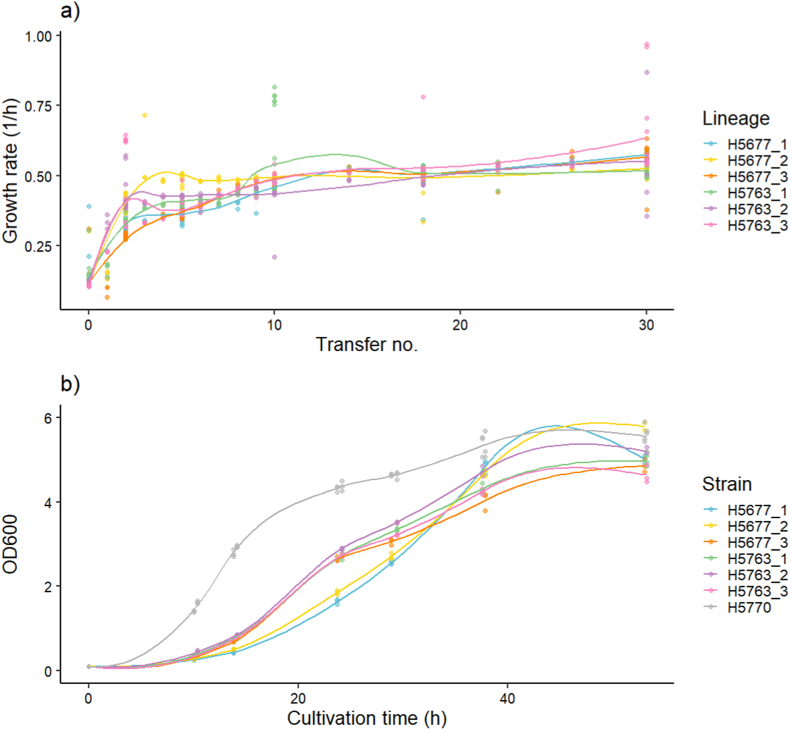
Growth characteristics of the parental strains (H5677 and H5763) and all evolved populations and isolated clones. Growth rates of three end-point populations of the evolution lineages (H5677_1 in blue, H5677_2 in yellow, H5677_3 in orange, H5763_1 in green, H5763_2 in purple and H5763_3 in pink) (a). Full lines are the *loess* fits to average growth rates of three to ten replicate cultures. Growth dynamics as culture turbidity (OD600) as a function of cultivation time of evolved isolates (H5677_1_B in blue, H5677_2_A in yellow, H5677_3_A in orange, H5763_1_C in green, H5763_2_C in purple, H5763_3_C in pink) and a control strain (H5770 in grey) (b). Full lines are the *loess* fits to average culture turbidities of three replicate cultures for evolved isolates and six biological replicates for the control strain.

Engineered *S. cerevisiae* CEN.PK113-7D (*icl1*Δ*1 ser3*Δ*1* s*er33*Δ*1 FAT2 OXA panE2*) clones (H5677 and H5763, Supplementary material) showed severely impaired growth on glucose and ammonium as sole carbon and nitrogen sources, respectively (Supplementary material), despite the rescue of glycine and L-serine synthesis by the synthetic oxalate pathway (Toivari et al., 2020). To select for enhanced growth and flux towards glycine and glyoxylate in the oxalate pathway, two equally engineered transformants (H5677 and H5763) were used to initiate three independent evolution lineages on glucose and ammonium as carbon and nitrogen sources, respectively. The adaptive evolution was performed as serial batch cultures with ca. 0.7 % transfers when visible turbidity was observed for over 210 generations (Fig. 4). The growth rate improved notably already within the first few transfers (i.e., by transfer 5, at least 35 generations), after which the increase was smaller but monotonous (Fig. 5a). Lag phase duration was substantially reduced (i.e., from at least 20 h to less than 10 h) in all lineages already during the early ALE (i.e., by transfer 10, at least 70 generations, Supplementary material). Three clones were isolated from each of the end-point populations of each adaptive evolution lineage and their growth dynamics were compared to the corresponding end-point populations using multi-well plate cultures. The evolved clones isolated from H5763 lineages at transfer 30, reached similar maximum specific growth rates but higher population densities than their respective populations (Table 2). In contrast, the evolved H5677 populations at transfer 30 all had higher final population densities than the isolated clones, with some end-point populations (H5677_1) also having a higher maximum specific growth rate (Table 2). Such behavior suggests of interdependences between the clones in the populations. A single evolved isolate from each evolution lineage was selected based on the growth performance (Supplementary material) for characterization in GA production.

**Table 2:** Maximum specific growth rates of the end-point populations and three isolated clones with standard deviations of three biological replicates. The maximum specific growth rates of the evolved isolated clones were tested for difference to the maximum specific growth rate of evolved end-point populations (one-way ANOVA and Tukey's test (n_control_ = 12, n_case_ = 3), P-value <0.01).

Lineage	Maximum specific growth rate (h^−1^):		Tukey's test
	Evolved end-point population	Evolved isolated clone	P-value
H5677_1	0.58 ± 0.02	0.460 ± 0.04	0.40
		0.400 ± 0.3	0.069
		0.300 ± 0.1	0.0016
H5677_2	0.52 ± 0.02	0.530 ± 0.06	1.0
		0.400 ± 0.2	0.064
		0.400 ± 0.1	0.042
H5677_3	0.57 ± 0.07	0.400 ± 0.2	0.072
		0.600 ± 0.1	0.83
		0.490 ± 0.09	0.61
H5763_1	0.51 ± 0.02	0.501 ± 0.004	0.40
		0.508 ± 0.003	0.86
		0.498 ± 0.001	0.27
H5763_2	0.60 ± 0.1	0.480 ± 0.002	0.71
		0.532 ± 0.005	0.99
		0.600 ± 0.1	1.0
H5763_3	0.60 ± 0.2	0.506 ± 0.006	0.39
		0.560 ± 0.007	0.76
		0.511 ± 0.001	0.42

To characterize the evolved isolates for GA production with respect to the control strain (H5770, having intact *ICL1*, *SER3* and *SER33*), one copy of gene *GLYR1* encoding glyoxylate reductase was genomically integrated in each (Fig. 3, Fig. 4). The evolved isolates with *GLYR1* achieved similarly high maximum population densities and maximum specific growth rates as the control strain (H5770) with *GLYR1* (Table 3, Fig. 5b). To investigate whether threonine aldolase route contributes to the growth of the evolved clones with *GLYR1*, *AGX1* encoding a glycine-glyoxylate transaminase was deleted from the evolved isolates with *GLYR1* (Fig. 4). Although the *agx1Δ1* strains had similar maximum specific growth rates as the evolved isolated clones with *GLYR1* (Table 4), the lag phases were much longer (i.e, ∼10 h and up to 17 h in evolved isolated clones with *GLYR1* and *agx1Δ1*, respectively) (Supplementary material). Thus, both pathways, the oxalate pathway and the route via threonine aldolase, appeared to contribute to the growth of evolved clones.

**Table 3:** Maximum specific growth rates of the parental strains (H5677 and H5763), evolved isolated clones with *GLYR1*, and the control strain (H5770) with standard deviations of three biological replicates. The maximum specific growth rates of the parental strains and the evolved isolated clones were tested for a difference to the maximum specific growth rate of the control strain (ANOVA and Tukey's test (n_control_ = 12, n_case_ = 3), P-value <0.01).

	Maximum specific growth rate (h-1)	Tukey's test P-value
H5677	0.068 ± 0.004	9.8E-12
H5763	0.064 ± 0.003	5.6E-12
H5677_1_B	0.210 ± 0.009	0.0024
H5677_2_A	0.160 ± 0.01	0.086
H5677_3_A	0.162 ± 0.007	0.48
H5763_1_C	0.167 ± 0.002	0.86
H5763_2_C	0.169 ± 0.001	0.96
H5763_3_C	0.178 ± 0.009	1.0
H5770	0.180 ± 0.02	na

na = not applicable.

**Table 4:** Maximum specific growth rates of the evolved isolated clones with *GLYR1* and the *agx1Δ1* mutants of evolved isolated clones with *GLYR1* with standard deviations of three biological replicates.

Maximum specific growth rate (h^−1^)	Evolved isolated clone with *GLYR1*	*agx1Δ1* mutant
H5677_1_B	0.180 ± 0.05	0.200 ± 0.02
H5677_2_A	0.220 ± 0.02	0.160 ± 0.07
H5677_3_A	0.240 ± 0.02	0.213 ± 0.006
H5763_1_C	0.236 ± 0.006	0.210 ± 0.01
H5763_2_C	0.210 ± 0.04	0.200 ± 0.02
H5763_3_C	0.270 ± 0.01	0.240 ± 0.02

### 2.6 ALE lineages acquired chromosomal copy number variations and single nucleotide variants in the pathways involved

To identify mutations enriched during ALE, the parental strains (H5677 and H5763), intermediate populations (i.e., at ten transfers, over 70 generations), end-point populations (i.e., at 30 transfers, over 210 generations), and a single isolated clone from the end-point populations of each ALE lineage, were whole-genome sequenced. The parental strains H5677 and H5763 were found to differ by a single missense variant in *DEP1* (197C > A, Pro66Gln) a component of the Rpd3L histone deacetylase complex (Carrozza et al., 2005) in H5677. In the ALE lineages started from the parental strains H5677 and H5763 the most recurringly mutated native gene was *RTK1* encoding a putative protein kinase whose deletion mutant has previously been observed to show an altered free amino acid availability in cells (Mülleder et al., 2016) (Table 5). Lineages H5677_2 and H5763_3 gained stop codons in *RTK1* (658C > T and 455C > G in H5677_2 and H5763_3, respectively) that were detected already in intermediate populations (i.e., at transfer ten). In addition, the isolated clone H5763_1_C had gained a missense mutation in *RTK1* (1290T > A, Asp430Glu). Furthermore, *DAL81* encoding a regulator of nitrogen metabolism had a mutation in the isolated clone H5677_2_A (1085C > A, Ala362Glu). Mutations were found also in metabolic enzyme encoding genes. Lineage H5763_2 had gained a missense mutation in *GLY1* (1159T > C, Tyr387His) encoding threonine aldolase that forms a route alternative to oxalate pathway to glyoxylate. Upstream of both threonine aldolase and oxalate pathway routes in metabolism (Fig. 3c), in *PYC1/2* encoding pyruvate carboxylase, a missense mutation (498C > A, Asp166Glu) was detected in the isolated clone H5677_2_A.

**Table 5:** Single nucleotide variants (SNVs) detected in high alternate allele frequences (AF) in the evolved lineages in the intermediate population (i.e., at ten transfers), the end-point population (i.e., at 30 transfers) and in the isolated evolved clones.

Lineage	Intermediate	End-point	Evolved isolated clone	SNV	Amino acid change
	population	population			
	(transfer 10)	(transfer 30)			
H5677_2	*RTK1* (AF = 1)	*RTK1* (AF = 0.98)	*RTK1* (AF = 1)	658C > T	Gln220∗
			*DAL81* (AF = 1)	1085C > A	Ala362Glu
			*PYC1/2* (AF = 1)	498C > A	Asp166Glu
H5677_3			*FAT2* (AF = 0.55)	1033A > T	Ile345Phe

H5763_1			*RTK1* (AF = 1)	1290T > A	Asp430Glu
			*CBP4* (AF = 1)	∗212C > A	Downstream
			*TGL4/5* (AF = 1)	−201C > A	Upstream
			hypothetical protein SCEN_O01930 (AF = 1)	1146A > T	Lys382Asn
H5763_2	*FKS1* (AF = 0.95)		*FKS1* (AF = 1)	4888G > T	Val1630Phe
	*GLY1* (AF = 0.93)		*GLY1* (AF = 1)	1159T > C	Tyr387His
		*FAT2* (AF = 0.50)	*FAT2* (AF = 0.53)	2956C > T	Leu986Phe
H5763_3	*RTK1* (AF = 0.51)	*RTK1* (AF = 0.69)	*RTK1* (AF = 0.98)	455C > G	Ser152∗
	*FRE1* (AF = 0.53)	*FRE1* (AF = 0.74)	*FRE1* (AF = 0.98)	2027A > G	Asp676Gly
	*STU1* (AF = 0.95)	*STU1* (AF = 1)	*STU1* (AF = 1)	470G > A	Ser157Asn
		*SIP3* (AF = 0.72)	*SIP3* (AF = 1)	2943T > C	Val981Val

The heterologous *FAT2* and *OXA* genes had also gained mutations during ALE. The intermediate population (i.e., at ten transfers) of H5763_2 lineage, the evolved isolated clone H5763_3_C, the end-point populations (i.e., at 30 transfers) of the lineages H5677_1 and H5677_3 all had a missense mutation in *OXA* gene (1396C > G, His457Asp, AF = 1). At ten transfers the mutation was already detected in the lineages H5677_1 and H5677_3 but in lower alternative allele frequency of 0.47. H5763_2 ALE end-point population (i.e., at 30 transfers) and the clone H5763_2_C isolated from that population had a missense mutation in the heterologous *FAT2* gene (2956C > T Leu986Phe). A different mutation in the *FAT2* gene (1033A > T, Ile345Phe) was detected also in the isolated clone H5677_3_A.

Since variants in the evolved isolated clones were detected in approximately 50 % allele frequency, ploidy of the clones was determined. The parental strains H5677 and H5763 were confirmed as haploids, but evolved isolated clones H5677_1_B, H5677_3_A and H5763_2_C were found to have become diploid during ALE. Evolved isolated clone H5677_2_A remained haploid throughout ALE, while H5763_1_C and H5763_3_C appeared to have acquired noticeable aneuploidy. Accordingly, copy number variant (CNV) segments (i.e., copy ratio to parental strain >1.5) were detected in all intermediate populations, end-point populations and evolved isolates (Supplementary material). Lineages originating from H5677 had gained in copy number in approximately the first half of chromosome V (1–319001), while lineages originating from H5763 had CNV segments detected throughout chromosome V (1–577587). Importantly, the early part of chromosome V (4001–116000), containing *GLY1* (69126–70289), was triplicated (i.e., copy ratio to parental strain >2.7) in lineage H5677_2 (intermediate population, end-point population and evolved isolated clone) and in intermediate populations of H5677_3 and H5763_3 (i.e., at ten transfers), end-point population H5763_2, and evolved isolated clones H5763_1_C and H5763_3_C.

### 2.7 Strains achieving enhanced glycolic acid production were found with minimum screening

**Fig. 6:**
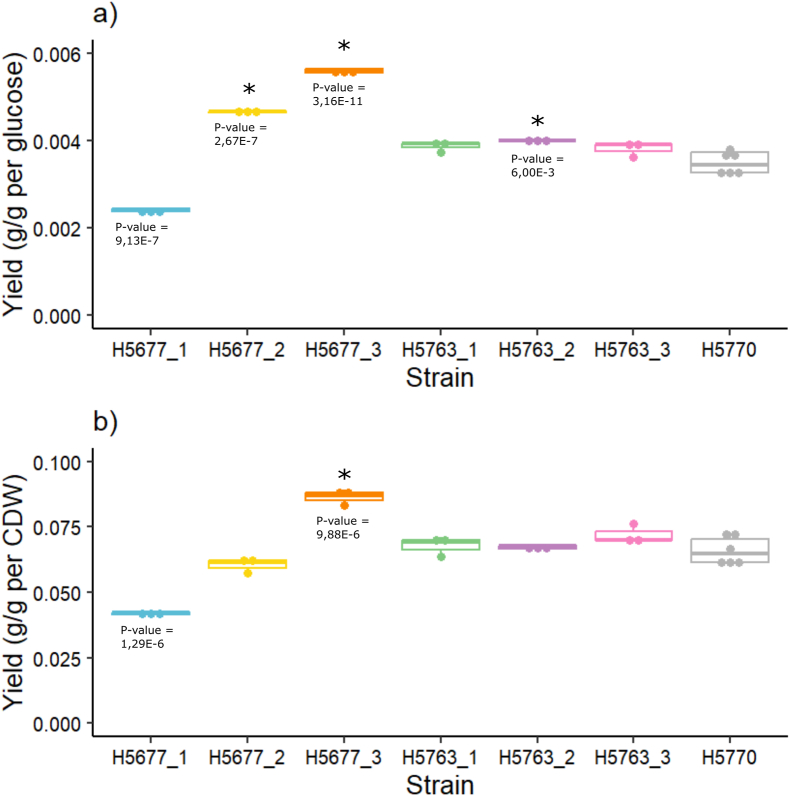
GA yield on glucose and on cell dry weight (CDW) (H5677_1_B in blue, H5677_2_A in yellow, H5677_3_A in orange, H5763_1_C in green, H5763_2_C in purple, H5763_3_C in pink and control H5770 in grey). GA yields on glucose at 53 h of cultivation for the evolved isolated clones and at 29 h of cultivation for the control strain (H5770) (a). Stars denote significantly higher yield compared to H5770 (ANOVA and Tukey's test; n_control_ = 6, n_sample_ = 3; g GA/g glucose; P-value <0.01) GA yields on biomass at 53 h for isolates and 29h for control of cultivation (b). Stars denote significantly higher yield compared to H5770 (ANOVA and Tukey's test; n_control_ = 6, n_sample_ = 3; g GA/g CDW; P-value <0.01).

**Fig. 7:**
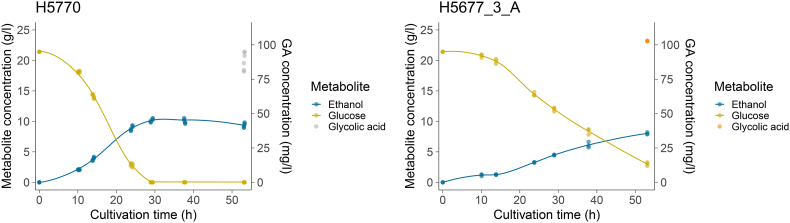
Glucose consumption, ethanol production, and glycolic acid (GA) final titre of the control strain (H5770) and the evolved isolate H5677_3_A. Glucose and ethanol concentrations over time are shown in dark yellow and in dark blue, respectively, on the left-hand y-axis. The curves are shown as loess fits to averages of three or six biological replicates. Final titre of GA is shown on the right-hand y-axis in grey for the control strain and orange for the evolved isolate H5677_3_A.

The evolved isolates consumed glucose and produced ethanol slower than the control strain H5770 (Fig. 7 and Supplementary material). To quantify differences in GA production, yields on glucose and cell dry weight (CDW) were determined when glucose had been consumed by the control strain, and the cultures of the isolates had 0–3 g/l glucose left (Fig. 6, Fig. 7). Yield on glucose was improved in three evolved isolated clones H5677_2_A (5 mg GA/g glucose; P-value = 2.66826E-07), H5677_3_A (6 mg GA/g glucose; P-value = 3.1562E-11), and H5763_2_C (4 mg GA/g glucose; P-value = 0.005998175) compared to the control strain H5770 (3 mg GA/g glucose) (ANOVA and Tukey's test; n_control_ = 6, n_sample_ = 3; P-value <0.01) (Fig. 5a). Yield on CDW was improved in the isolated clone H5677_3_A (90 mg GA/g CDW; P-value = 0.0000099) compared to the control strain H5770 (70 mg GA/g CDW) (ANOVA and Tukey's test; n_control_ = 6, n_sample_; P-value <0.01) (Fig. 5b). The evolved isolate H5677_3_A achieved the highest GA titer of 103 mg/l (P-value 0.00089) after 53 h of cultivation and utilization of 18.4 g glucose, higher than the control strain (88 mg/l) (ANOVA and Tukey's test; n_control_ = 6, n_sample_ = 3; P-value <0.01).

## 3 Discussion

In this work a model-guided ALE design was extended to scoring both conditions affecting the flux-couplings in cells, the chemical environment and the metabolic network structure. The new algorithm EvolveXGA found Darwinian selection strategies for enhancing heterologous production in *S. cerevisiae* using ALE for all 29 evaluated products. The heterologous pathways or native reactions that supply precursors, energy, and/or redox power could be coupled to fitness with combinations of maximum of four reaction knock outs and three nutrients in the chemical environment. Von Kamp and Klamt (2017) showed that growth-product coupling is achievable in aerobic conditions for almost all compounds in five different organisms including *S. cerevisiae*. However, growth-coupling of some of the compounds required more than 20 gene deletions which is likely to lead to inviability of cells. In addition, simple evolution environments are preferred due to uncertainty on organisms’ nutritional preferences (Tramontano et al., 2018). With kinetic data the nutritional preferences could be incorporated in the developed design method by replacing the GEM with e.g., a convex kinetic model of metabolism (Taylor et al., 2024).

Reducing the metabolic network with such a reasonable number of reactions as maximum of four is not expected to be detrimental for production strains. Here, the triple deletion strains of *S. cerevisiae* improved fitness notable in the short-term ALE. Previously, *S. cerevisiae* single gene deletion mutants have been shown to rapidly restore near wild type growth (Szamecz et al., 2014). Adaptations that restored growth were found specific to the deleted function, but the compensatory mutations were rarely detected in gene duplicates, which favors the metabolic network reduction as a means to enhance redirected fluxes using ALE. Successful examples of redirected flux enhancement have been made when adaptively evolving substantially modified, growth-product coupled, microbial strains (Fong et al., 2005; Tokuyama et al., 2018).

The power of adaptive evolution in redistributing flux in central metabolism of cells is evident in the functions of the genes found with enriched mutations in this study. A transcription factor, a protein kinase and metabolic enzymes span the multiple regulatory levels that control the central metabolic fluxes and make it challenging to alter the flux distribution by targeted engineering (Daran-Lapujade et al., 2007; Nielsen and Keasling, 2016). Networks of protein kinases and their targets have been characterized as highly redundant (Oliveira et al., 2015; Ptacek et al., 2005). Yet, in this specific case, mutations in a single protein kinase *RTK1* appeared to enable adaptation of cells.

Here, the EvolveXGA method allowed identification of a strain with improved GA production with minimal screening of adapted clones. The short-term ALE achieved a minor production improvement. The demonstrated heterologously expressed glyoxylate reductases have been NADPH dependent enzymes (Salusjärvi et al., 2019). Previous works have tried to ameliorate this by enhancing NADPH availability with the inclusion of a nicotinamide adenine dinucleotide phosphate (NADP)-dependent glyceraldehyde-3-phosphate dehydrogenase (GapN) (Kakko et al., 2023) or by converting glycolaldehyde to GA, to avoid NADPH requirements (Carniel et al., 2023; Senatore et al., 2024). Improving the availability of NADPH, perhaps via ALE, is the solution for further improving the GA production from glucose.

## 4 Material and methods

### 4.1 GEM

Three first reactions of oxalate pathway for GA synthesis (Toivari et al., 2020) and other heterologous pathways (Jouhten et al., 2016) (Supplementary material) were individually introduced into the *S. cerevisiae* consensus genome-scale model (Lu et al., 2019), v. 8.3.5 (Sánchez et al., 2020). In addition, some modifications to flux lower and upper bounds were made (Supplementary material). Furthermore, the search space was limited to increase the probability of finding optimal flux-coupling solution as genetic algorithms do not search the whole search space. Removed reactions included essential reactions for growth; essential reactions for the heterologous production in question; blocked reactions (i.e. reactions without flux in any nutritional environment); reactions without gene annotation; transport, exchange and diffusion reactions; and some additional reactions such as reactions taking part to lipid metabolism (Supplementary material). In addition, components of the chemical environment were searched from a limited amount of carbon and nitrogen sources (Supplementary material). The total nutrient utilization was restricted to 75 mmol/g CDW when searching for fitness coupling strategies combining evolution environment and reaction knock outs for native precursor, energy and redox power providing reactions and oxalate pathway. Flux bounds were set to −1000 and 1000 and the growth yield to an arbitrary 10 when reaction knock out strategies were searched for oxalate pathway and the flux bounds were set to −20 000 and 20000 and growth yield to 1 when combined evolution environment and reaction knock out strategies were searched. Jupyter notebook v. 6.0.3, python v. 3.7.6 and cobrapy v. 0.18.1 (https://github.com/opencobra/cobrapy) were used to manipulate the model.

### 4.2 EvolveXGA algorithm

Pyeasyga genetic algorithm (https://github.com/remiomosowon/pyeasyga) was used as a starting point for the development of EvolveXGA. Individuals in the genetic algorithm were scored for the predicted total response to selection of the flux targets in worst-case scenario i.e., as the minimum sum of the minimum up-regulation target fluxes and the additive inverse maximum down-regulation target fluxes given arbitrary fixed growth (i.e., as a proxy of fitness) (Equation (1)). Thus, the score represents the worst-case sum of flux couplings of the target fluxes to growth. This scoring was previously derived by Jouhten et al. (2022) from the Robertson-Price identity (Price, 1970; Rausher, 1992; Robertson, 1966, 1968) and the flux couplings as indications of genetic dependency (Notebaart et al., 2008). Thus, the score has the units of a flux per growth (i.e., mmol/(g CDW)) and solving it does not require information on the *in vivo* uptake rates of compounds in the chemical environment. EvolveXGA was run with python v. 3.7.6 (simulations for GA pathway) or v. 3.6.8 (simulations for other heterologous production pathways). IBM ILOG CPLEX v. 12.10 was used as a linear programming (LP) and mixed-integer linear programming (MILP) problem solver.

Equation 1 $$\min\sum_{\left|u\right|=1}^{k}v_{\left|u\right|}+\sum_{\left|d\right|=1}^{l}\left({-v}_{\left|d\right|}\right)$$

s.t.

S·v=0

$$v_{\mu }=a$$

$$\begin{array}{cc} v_{n}\leq -1 & n\in N \end{array}$$

$$\begin{array}{cc} v_{inh}=0 & inh\in I \end{array}$$

$$v_{lb}\leq v\leq v_{ub}$$

$$c\cdot v\geq r_{uptake,\max}$$

$$\begin{array}{ccc} 0\leq v_{\left|u\right|}\leq v_{\left|u\right|,ub} & \left|u\right|\in U & v_{\left|u\right|,ub}=\max\left(v_{u,lb},v_{u,ub}\right) \end{array}$$

$$\begin{array}{ccc} 0\leq v_{\left|d\right|}\leq v_{\left|d\right|,ub} & \left|d\right|\in D & v_{\left|d\right|,ub}=\max\left(v_{d,lb},v_{d,ub}\right) \end{array}$$

$$\begin{array}{cc} v_{u}-v_{\left|u\right|}\leq 0 & u\in H \end{array}$$

$${-v}_{u}-v_{\left|u\right|}\leq 0$$

$$\begin{array}{cc} v_{d}-v_{\left|d\right|}\leq 0 & d\in L \end{array}$$

$${-v}_{d}-v_{\left|d\right|}\leq 0$$

$${-v}_{d}-{M\cdot d_{\left|d\right|}+v}_{\left|d\right|}\leq 0$$

$$\begin{array}{cc} v_{d}+{M\cdot d_{\left|d\right|}+v}_{\left|d\right|}\leq M & d_{\left|d\right|}\;\epsilon \;\left\{0,1\right\} \end{array}$$

where *r*_*uptake,max*_ is the minimum absolute total uptake from the chemical environment for an arbitrary fixed growth *a* (set to 10 or 1 when the chemical environment was fixed or free, respectively), *H* and *L* are the sets of flux targets (and *k* and *l* the sizes of sets) requiring up- and down-regulation, respectively, and *U* and *D* are sets of absolute flux variables representing the flux targets requiring up- and down-regulation, respectively. *v*_*|u|,ub*_ and *v*_*|d|,ub*_ are the upper bounds of the absolute flux variables whose values were derived as maximum absolute value of the flux bounds *v*_*lb*_ and *v*_*ub*_. *M* is a parameter for which a value of twice the flux bounds should be used. It is double the maximum flux upper bound. *d*_*|d|*_ is a binary variable introduced for each reversible flux target that requires down-regulation.

### 4.3 Determination of precursor, energy, and redox power providing native reactions

The identification of precursor, energy and redox power providing native reactions was formulated as a MILP problem similarly to ROOM (Shlomi et al., 2005). ROOM predicts mutant phenotypes by minimizing the number of flux changes beyond thresholds after gene deletions compared to wild type flux state. ROOM was modified to instead of the constraint introducing the gene deletion to have heterologous production above a threshold level as a constraint. Thus, a minimum set of flux changes when cellular objective was changed from growth to production was solved. From this flux set reactions with upregulated flux were extracted and formed the set of native precursor, energy and redox providing reactions. The constraint on heterologous production was set to a minimum of 99 % of the maximum theoretical production and δ and ε parameters used in ROOM formulation were set to 0.5 and 0.00001, respectively.

### 4.4 Cultivation media

*E. coli* was grown in lysogeny broth (LB) with 100 μg/ml ampicillin (MERCK). For transformations, yeast cells were cultivated in YPD containing 20 g/l peptone (VWR Chemicals), 10 g/l yeast extract (OXOID) and 20 g/l D-glucose (VWR Chemicals). For selection and maintenance of plasmids, selective YPD media with 200 μg/ml nourseothricin dihydrogen sulfate (NAT) (Jena Bioscience, AB-101) and/or 200 μg/ml Geneticin® G-418 sulfate powder (G418) (MERCK) was used.

For ALE, growth characterisation and genome extraction, a 10x stock solution of yeast nitrogen base (YNB) media was used. The 10x stock, containing 6.8 g YNB without amino acids (BD Diagnostic Systems) and 5 g D-glucose (VWR Chemicals) in 100 mL distilled de-Ionized water (DDIW), was diluted with DDIW to 1x solution for culturing.

For metabolite analysis, *S. cerevisiae* strains were grown in synthetic defined medium (SDM) with 6.7 g/l YNB without amino acids (BD Diagnostic Systems) and 20 g/l D-glucose (VWR Chemicals).

### 4.5 Plasmid construction

The plasmids constructed and used in this study are listed in Supplementary material. Restriction enzymes were obtained from Thermo Scientific (USA), New England BioLabs (USA) and Roche (Switzerland). Oligos were ordered from MERCK life science Oy (Germany) or Integrated DNA technologies (IDT) (USA). Amplified polymerase chain reaction (PCR) products and digested DNA fragments were purified before cloning with gel electrophoresis using 0.8 % agarose and QIAquick PCR Purification Kit (Qiagen, Netherlands), or with Monarch PCR & DNA Cleanup Kit (NewEngland BioLabs). Plasmid isolation was done with the GeneJET Plasmid Miniprep Kit (Thermo Scientific).

All integration plasmids were constructed according to manufacturer's protocol using Kapa Hifi enzyme (Kapa Biosystems), EasyClone-Markerfree integrative expression vectors (Jessop-Fabre et al., 2016) obtained from Addgene, and Gibson Assembly (E2611S, New England BioLabs). gRNA plasmids complementary to each integrative vector were obtained from Addgene. The *ICL1*, *SER3*, *SER33* and *AGX1* deletion cassettes and gRNA fragments were amplified with Kapa Hifi enzyme. The EasyClone XI-2 gRNA plasmid (pCfB3044) was used as a template for all constructed gRNAs, with the original gRNA sequence replaced with correct crRNA for each native gene, and the plasmids ligated using Gibson Assembly. All ligated plasmids were transformed into *E. coli* TOP10 by electroporation (Dower et al., 1988) and verified with analytical digestion and Sanger sequencing (Microsynth).

### 4.6 Strain construction

The *S. cerevisiae* strains used and constructed in this work are listed in Supplementary material. *S. cerevisiae* CEN.PK parent strains were kindly provided by Dr. P. Kötter (Institut für Mikrobiologie, J.W. Goethe Universität Frankfurt, Germany). The heterologous genes integrated into strains are listed in Supplementary material.

All *S. cerevisiae* transformations were done using the standard lithium acetate protocol (Gietz, 2014) and the CRISPR/Cas9 protocol of the EasyClone-Markerfree kit (Jessop-Fabre et al., 2016), with expression plasmids linearized by NotI enzyme (FD0596, Thermo Scientific). Correct integration was confirmed with DreamTaq colony PCR (Thermo Scientific).

The native genes *ICL1*, *SER3* and *SER33* were deleted with constructed deletion casettes and gRNAs. The following genes for GA pathway were integrated as in Kakko et al. (2023): *FAT2*, *OXA* and *panE2*. After ALE, *GLYR1* was integrated as previously described. *AGX1* was then deleted with the constructed deletion casette and gRNA.

### 4.7 ALE and clone isolation

From each of the two separate transformants of the initial synthetically modified strain (H5677 and H5763) three replicate evolution lineages were initiated. Cells were cultivated in 20 ml YNB medium with glucose in 100 ml Erlenmeyer flasks. Cultures were visually assessed for notable turbidity before each transfer. Subsequent transfers were made in fresh medium with a starting OD600 of 0.02.

Cells were cultivated in the beginning for as many days as necessary to achieve higher turbidity cultures. As growth improved and stabilized, transfers were completed every two to three nights. ALE was continued for a total of 30 transfers, with intermediate cultures collected and cryogenically stored. Population samples were preserved in glycerol stocks during each transfer for transfers until ten, and then every four transfers from transfers ten to 30.

From the final adapted populations, cells from glycerol stocks were plated on YPD plates to randomly collect isolated colonies for characterization. Only three colonies from each lineage were chosen for screening. The isolates were grown in YNB medium before preservation in glycerol stock and growth characterisation.

### 4.8 Growth characterisation

Before choosing final isolates for GA production, growth characterization was performed on the populations of all six lineages at each available transfer, as well as the randomly selected single colonies. Cells from glycerol stocks were pre-cultured in 2.5 ml YNB medium with 5 % glucose. Then, the pre-cultures were used to inoculate 200 μl of YNB medium with 5 % glucose into an OD600 of 0.02, on a 100-well Honeycomb Microplate (Bioscreen), with 3–10 replicates per strain. Cells were cultured for over 100 h. Bioscreen assays were run at +30 °C, normal speed, with continuous shaking and medium amplitude in Bioscreen machine FP-1100-C (Labsystems). Filter 600 nm brown was used to measure OD every 30 min.

### 4.9 Cultivations for metabolite analysis

For metabolite analysis, all cultures were cultivated in 50 ml SDM (in 250 ml flasks) at +30 °C with 200–220 rpm shaking. First, pre-cultures were initiated from glycerol stocks and incubated for 2–3 days. Then, the pre-cultures were used to inoculate fresh medium into an OD600 of 0.2 to create second pre-cultures. Second pre-cultures were incubated for two days after which they were used to inoculate fresh medium into an OD600 of 0.1 to start the cultivations for metabolite analysis.

### 4.10 HPLC metabolite analysis

Culture supernatant samples (1.2 ml) were prepared by pelleting the cells by centrifugation at room temperature (3 min, 4000 rpm). The concentrations of metabolites in the supernatants were measured with Vanquish (Thermo Fisher) high-performance liquid chromatography (HPLC) system using Aminex HPX-87H (Bio-Rad) column. The column was eluted with 5 mmol/l H_2_SO_4_ as mobile phase with a flow rate of 0.5 ml/min, at column temperature of +55 °C. Glucose, glycerol, ethanol, and acetate were detected with an RI detector (RefractoMax521), and glycolic acid with an UV/VIS detector (VC-D11-A, set to 210 nm). Chromeleon software v. 7.3.1 was used for the data processing. Linear standard curves of reference standards (Merck) prepared in DDIW were used for quantification.

### 4.11 Growth rate calculations and statistical analysis

All data from multiwell plate cultivation using Bioscreen FP-1100-C was converted to equivalent spectrophotometer values as in Warringer and Blomberg (2003). The growth rates were calculated using R v. 4.3.2 with package growthcurver v. 0.3.1 (Sprouffske and Wagner, 2016).

Statistical testing of differences in growth rates and yields was performed with ANOVA and Tukey's test (TukeyHSD), with R v. 4.3.2 and dplyr v. 1.1.4 package.

### 4.12 Genomic DNA extraction

The parental populations (H5677 and H5763), best performing isolates, and intermediate populations ten and 30, from each evolved lineage, were sent for whole-genome sequencing. Glycerol stocks of the chosen strains were inoculated into 25 mL of YNB medium for pre-cultures and then cultivated in fresh YNB medium for two nights. Cells were pelleted and washed twice in 10 mL sterile MilliQ water by centrifuging at 4000×*g* for 2 min. Genomic DNA was extracted from pellets using the MasterPure™ Yeast DNA Purification Kit (MPY80200 Lucigen, BioSearch Tech) according to manufacturer's protocol.

### 4.13 Whole genome sequencing

Library preparations and NGS sequencing was performed by Novogene on Illumina NovaSeq 6000 sequencing system (Microbial Whole Genome Sequencing (WOBI) producing PE150 reads). The raw reads of genomic DNA samples (Supplementary material) were deposited in ENA database (https://www.ebi.ac.uk/ena/) in project PRJEB49512.

### 4.14 Whole genome sequence data analysis

The quality of the obtained reads was checked using Fastqc v. 0.11.4 (Andrews, 2010). Adapter removal and low quality read filtering was performed using cutadapt v. 1.9.1 (Martin, 2011). The trimmed reads were aligned to *S. cerevisiae* CEN.PK113-7D reference genome (GCA_002571405.2_ASM257140v2) with the Burrows-Wheeler Aligner v. 0.7.12 mem (Li and Durbin, 2009) using default parameters. The alignments were processed (added read groups, sorted, reordered, and indexed) and duplicate reads were marked using Picard Tools v. 1.129 (Poplin et al., 2017; Van der Auwera et al., 2013). SNV and indel variant calling was performed against the parental sample with GATK4 v. 4.1.0.0 (Poplin et al., 2017; Van der Auwera et al., 2013) Mutect2 using *S. cerevisiae* CEN.PK113-7D (GCA_002571405.2_ASM257140v2) as the reference genome and default parameters. The variant calls were filtered using GATK4 v. 4.1.0.0 (Poplin et al., 2017; Van der Auwera et al., 2013) FilterMutectCalls using default thresholds. The same pipeline was run separately for the heterologous genes *FAT2*, *OXA* and *panE2*, with the sequences listed in Supplementary material used as alignment references. Copy number variation (CNV) analysis of native genes was run on the previously processed and aligned BAM files using GATK4 v. 4.1.0.0 (Poplin et al., 2017; Van der Auwera et al., 2013).

### 4.15 Ploidy analysis

The ploidy of parental strains (H5677, H5763), used to initiate ALE, and isolates obtained after ALE (H5971-H5976) was determined using flow cytometry as described in Krogerus et al. (2017), with slight modifications. The isolates were grown overnight in YPD media in +30 °C, 220 rpm. Approximately 1 ∗ 10^7^ of cells were then pelleted and washed with 400 μl of 50 mM sodium citrate (pH 7.2). 950 μl of ice cold 100 % ethanol was added to obtain final ethanol concentration of 70 % and cells were fixed overnight in −20 °C. Cells were pelleted, washed with 800 μl of 50 mM sodium citrate (pH 7.2), pelleted again and resuspended in 500 μl of RNAse A solution containing 0.25 mg/ml RNAse A in 50 mM Sodium citrate (pH 7.2) and incubated overnight in +37 °C. Next, 50 μl of 20 mg/ml proteinase K was added to reach final concentration of 1 mg/ml and the cells were incubated for 1 h in +50 °C. Finally, 500 μl of 50 mM sodium citrate (pH 7.2) with 4 μM Sytox Green (Invitrogen, 5 mM in DMSO) was added and ploidy of the isolates was analyzed using FACSAria Ilu cytometer (Becton-Dickinson, USA). Ploidy of the isolates was determined by comparing fluorescence intensities with fluorescence intensities obtained for *S. cerevisiae* CEN.PK 113-1A haploid strain and CEN.PK2 diploid strain.

### 4.16 Data analysis and visualization

Data analysis and visualization was performed using R v. 4.3.2 and packages ggplot2 v. 3.5.1, wesanderson v. 0.3.7, RColorBrewer v. 1.1–3, cowplot v. 1.1.3, tidyverse v. 2.0.0.

## CRediT authorship contribution statement

**Natalia Kakko von Koch:** Writing – original draft, Visualization, Investigation, Formal analysis. **Tuula Tenkanen:** Writing – original draft, Visualization, Software, Investigation, Formal analysis. **Sandra Castillo:** Writing – review & editing, Software. **Virve Vidgren:** Writing – review & editing, Investigation. **Tino Koponen:** Writing – review & editing, Investigation. **Kristoffer Krogerus:** Writing – review & editing, Investigation. **Merja Penttilä:** Writing – review & editing, Supervision, Funding acquisition. **Paula Jouhten:** Writing – review & editing, Writing – original draft, Supervision, Investigation, Funding acquisition, Formal analysis, Conceptualization.

## Declaration of competing interest

The authors declare the following financial interests/personal relationships which may be considered as potential competing interests: Paula Jouhten reports financial support was provided by Research Council of Finland. Paula Jouhten reports financial support was provided by Novo Nordisk Foundation. Merja Penttila reports financial support was provided by Jenny and Antti Wihuri Foundation. Tuula Tenkanen reports financial support was provided by Jenny and Antti Wihuri Foundation. If there are other authors, they declare that they have no known competing financial interests or personal relationships that could have appeared to influence the work reported in this paper.

## Data Availability

Raw whole-genome sequencing was stored in ENA project: PRJEB49512. Python implementation of EvolveXGA is found in https://github.com/vttresearch/EvolveXGA.
