# Supplementary material for: Model-guided chemical environment and metabolic network design to couple pathways with cell fitness
Source: Metab Eng Commun. 2025 Nov 22;21:e00267. doi: 10.1016/j.mec.2025.e00267 (PMC12686821; doi:10.1016/j.mec.2025.e00267)
Supplement: Multimedia component 1 [file mmc1.docx]

Supplementary material

**Model-guided chemical environment and metabolic network design to couple pathways with cell fitness**

Natalia Kakko von Koch2*, Tuula Tenkanen1*, Sandra Castillo1, Virve Vidgren1, Tino Koponen1, Kristoffer Krogerus1, Merja Penttilä1, Paula Jouhten2!

1 VTT technical Research Centre of Finland Ltd

2 Aalto University, School of Chemical Engineering, Department of Bioproducts and Biosystems

*These authors contributed equally

!Corresponding author

**List of Supplementary Tables**

Supplementary Table 1: Synthetic production pathways for 28 different compounds.

Supplementary Table 2: Reactions removed from possible reaction deletion targets.

Supplementary Table 3: Possible compounds for chemical environment nutrients.

Supplementary Table 4: Darwinian selection strategies for synthetic pathways.

Supplementary Table 5: Darwinian selection strategies for native flux targets.

Supplementary Table 6: Oxalate pathway.

Supplementary Table 7: Strains used and constructed.

Supplementary Table 8: Modifications to flux lower and upper bounds.

Supplementary Table 9: Plasmids used and constructed.

Supplementary Table 10: Heterologous genes used.

Supplementary Table 11: ENA codes for sequencing results.

Supplementary Table 12: Heterologous integration casettes for *FAT2*, *OXA* and *panE2*, used as SNV reference sequence.

**List of Supplementary Figures**

Supplementary Figure 1: Growth dynamics of parental strains H5677 and H5763.

Supplementary Figure 2: Bioscreen growth profiles for all six evolution lineages.

Supplementary Figure 3: Bioscreen growth profiles of end-point populations compared to evolved isolates for all six evolution lineages.

Supplementary Figure 4: Growth of evolved isolates with or without *agx1Δ1* deletion*.*

Supplementary Figure 5: Copy number variation (CNV) in chromosome V.

Supplementary Figure 6: HPLC data for *GLYR1* integrated isolates and H5770 control strain.

**Supplementary Table 1.** Synthetic production pathways for 28 different compounds obtained from Jouhten *et al*., 2016.

| **4-Methylthiobutyl-desulfoglucosinolate** | | | | |
| --- | --- | --- | --- | --- |
| methionine-oxo-acid transaminase | 2-oxoglutarate [cytoplasm] + L-methionine [cytoplasm] |  | <=> | L-glutamate + 4-methylthio-2-oxobutanoic acid [cytoplasm] |
| methylthioalkylmalate synthase 1 | acetyl-CoA + 4-methylthio-2-oxobutanoic acid [cytoplasm] + H2O |  | <=> | coenzyme A + 2-(2-methylthio)ethylmalic acid [cytoplasm] |
| 2-(2-methylthio)ethylmalate hydroxymutase | 2-(2-methylthio)ethylmalic acid [cytoplasm] |  | <=> | 3-(2-methylthio)ethylmalic acid [cytoplasm] |
| 3-(2-methylthio)ethylmalate oxidoreductase (decarboxylating) | 3-(2-methylthio)ethylmalic acid [cytoplasm] |  | <=> | CO2 + 2-Oxo-5-methylthiopentanoic acid [cytoplasm] |
| methylthioalkylmalate synthase like | acetyl-CoA + 2-Oxo-5-methylthiopentanoic acid [cytoplasm] + H2O |  | <=> | coenzyme A + 2-(3-methylthio)propylmalic acid [cytoplasm] |
| 2-(3-methylthio)propylmalate hydroxymutase | 2-(3-methylthio)propylmalic acid [cytoplasm] |  | <=> | 3-(3-methylthio)propylmalic acid [cytoplasm] |
| 3-(3-methylthio)propylmalate oxidoreductase | 3-(3-methylthio)propylmalic acid [cytoplasm] |  | => | CO2 + 2-oxo-6-methylthiohexanoic acid [cytoplasm] |
| 2-oxo-acid aminotransferase | L-glutamate + 2-oxo-6-methylthiohexanoic acid [cytoplasm] |  | <=> | 2-oxoglutarate + dihomomethionine [cytoplasm] |
| dihomomethionine,NADPH:oxygen oxidoreductase  (N-hydroxylating, decarboxylating) | 2 H(+) + 2 NADPH + 2 oxygen + dihomomethionine [cytoplasm] |  | => | CO2 + 2 NADP(+) + 5-methylthiopentanaldoxime [cytoplasm] + 3 H2O |
| 5-methylthiopentanaldoxime,NADPH:oxygen oxidoreductase | H(+) + L-cysteine + NADPH + oxygen + 5-methylthiopentanaldoxime [cytoplasm] |  | => | NADP(+) + S-(5-Methylthiopentylthiohydroximoyl)-L-cysteine [cytoplasm] + 2 H2O |
| S-(5-methylthiopentylthiohydroximoyl)-L-cysteine aminolyase | S-(5-Methylthiopentylthiohydroximoyl)-L-cysteine [cytoplasm] + H2O |  | => | NH3 + pyruvate + 5-methylthiopentylthiohydroximate [cytoplasm] |
| N-hydroxythioamide S-beta-glucosyltransferase | UDP-glucose + 5-methylthiopentylthiohydroximate [cytoplasm] |  | => | UDP + 4-methylthiobutyl-desulfoglucosinolate [cytoplasm] |
| 4-methylthiobutyl-desulfoglucosinolate transport | 4-methylthiobutyl-desulfoglucosinolate [cytoplasm] |  | => | 4-methylthiobutyl-desulfoglucosinolate [extracellular] |
| **6-Methylsalicylate** | | | | |
| 6-methylsalicylate synthase | acetyl-CoA + H(+) + 3 malonyl-CoA [cytoplasm] + NADPH |  | => | 3 CO2 + 4 coenzyme A + NADP(+) + 6-methylsalicylate [cytoplasm] + H2O |
| 6-methylsalicylate transport | 6-methylsalicylate [cytoplasm] |  | => | 6-methylsalicylate [extracellular] |
| 6-methylsalicylate exchange | 6-methylsalicylate [extracellular] |  | => |  |
| **8-Epi-cedrol** | | | | |
| epi-cedrol synthase | 2-trans,6-trans-farnesyl diphosphate [cytoplasm] |  | <=> | diphosphate + 8-epi-cedrol [cytoplasm] |
| 8-epi-cedrol transport | 8-epi-cedrol [cytoplasm] |  | => | 8-epi-cedrol [extracellular] |
| 8-epi-cedrol exchange | 8-epi-cedrol [extracellular] |  | => |  |
| **Amorpha-4,11-diene** | | | | |
| amorphadiene synthase | 2-trans,6-trans-farnesyl diphosphate [cytoplasm] |  | <=> | diphosphate + amorpha-4,11-diene [cytoplasm] |
| amorpha-4,11-diene transport | amorpha-4,11-diene [cytoplasm] |  | => | amorpha-4,11-diene [extracellular] |
| amorpha-4,11-diene exchange | amorpha-4,11-diene [extracellular] |  | => |  |
| **Apigenin** | | | | |
| tyrosine ammonia lyase | L-tyrosine [cytoplasm] |  | <=> | NH3 + p-hydroxycinnamic acid [cytoplasm] |
| 4-coumaroyl:CoA ligase | ATP + coenzyme A + p-hydroxycinnamic acid [cytoplasm] |  | <=> | AMP + diphosphate + 4-coumaroyl-CoA [cytoplasm] |
| naringenin chalcone synthase | 3 malonyl-CoA + 4-coumaroyl-CoA [cytoplasm] |  | <=> | 3 CO2 + 4 coenzyme A + naringenin chalcone [cytoplasm] |
| naringenin chalcone isomerase | naringenin chalcone [cytoplasm] |  | <=> | naringenin [cytoplasm] |
| flavone synthase, apigenin | 2-oxoglutarate + oxygen + naringenin [cytoplasm] |  | <=> | CO2 + succinate + apigenin [cytoplasm] + H2O |
| apigenin transport | apigenin [cytoplasm] |  | => | apigenin [extracellular] |
| apigenin exchange | apigenin [extracellular] |  | => |  |

| **Artemisinic acid** | | | | |
| --- | --- | --- | --- | --- |
| amorphadiene synthase | 2-trans,6-trans-farnesyl diphosphate [cytoplasm] |  | <=> | diphosphate + amorpha-4,11-diene [cytoplasm] |
| amorpha-4,11-diene monooxygenase1 | H(+) + NADPH + oxygen + amorpha-4,11-diene [cytoplasm] |  | => | NADP(+) + artemisinic alcohol [cytoplasm] + H2O |
| amorpha-4,11-diene monooxygenase2 | H(+) + NADPH + oxygen + artemisinic alcohol [cytoplasm] |  | => | NADP(+) + artemisinic aldehyde [cytoplasm] + 2 H20 |
| amorpha-4,11-diene monooxygenase3 | H(+) + NADPH + oxygen + artemisinic aldehyde [cytoplasm] |  | => | NADP(+) + artemisinic acid [cytoplasm] + H20 |
| artemisinic acid transport | artemisinic acid [cytoplasm] |  | => | artemisinic acid [extracellular] |
| artemisinic acid exchange | artemisinic acid [extracellular] |  | => |  |
| **Beta-carotene** | | | | |
| geranylgeranyl diphosphate synthase | isopentenyl diphosphate [cytoplasm] +  2-trans,6-trans-farnesyl diphosphate [cytoplasm] |  | => | diphosphate + geranylgeranyl diphosphate [cytoplasm] |
| all-trans-phytoene synthase | 2 geranylgeranyl diphosphate [cytoplasm] |  | => | 2 diphosphate + all-trans-phytoene [cytoplasm] |
| all-trans-phytoene monooxygenase | FAD + all-trans-phytoene [cytoplasm] |  | => | FADH2 + all-transphytofluene [cytoplasm] |
| all-trans-phytofluene monooxygenase | FAD + all-transphytofluene [cytoplasm] |  | => | FADH2 + zeta-carotene [cytoplasm] |
| zeta-carotene monooxygenase | FAD + zeta-carotene [cytoplasm] |  | => | FADH2 + neurosporene [cytoplasm] |
| neurosporene monooxygenase | FAD + neurosporene [cytoplasm] |  | => | FADH2 + lycopene [cytoplasm] |
| beta-carotene synthase | lycopene [cytoplasm] |  | => | beta-carotene [cytoplasm] |
| beta-carotene transport | beta-carotene [cytoplasm] |  | => | beta-carotene [extracellular] |
| beta-carotene exchange | beta-carotene [extracellular] |  | => |  |
| **Butanol** | | | | |
| (R)-3-Hydroxybutanoyl-CoA:NAD+ oxidoreductase | acetoacetyl-CoA [cytoplasm] + H(+) + NADH |  | <=> | NAD(+) + (R)-3-hydroxybutanoyl-CoA [cytoplasm] |
| 3-hydroxybutyryl-CoA dehydratase | (R)-3-hydroxybutanoyl-CoA [cytoplasm] |  | <=> | crotonoyl-CoA [cytoplasm] + H2O |
| trans-2-enoyl-CoA reductase | H(+) + NADH + crotonoyl-CoA [cytoplasm] |  | <=> | NAD(+) + butanoyl-CoA [cytoplasm] |
| butanal dehydrogenase | H(+) + NADH + butanoyl-CoA [cytoplasm] |  | <=> | coenzyme A + NAD(+) + butanal [cytoplasm] |
| butanol dehydrogenase | H(+) + NADH + butanal [cytoplasm] |  | <=> | NAD(+) + butanol [cytoplasm] |
| butanol transport | butanol [cytoplasm] |  | => | butanol [extracellular] |
| butanol exchange | butanol [extracellular] |  | => |  |
| **Chrysin** | | | | |
| tyrosine ammonia lyase | L-tyrosine [cytoplasm] |  | <=> | NH3 + p-hydroxycinnamic acid [cytoplasm] |
| 4-coumaroyl:CoA ligase | ATP + coenzyme A + p-hydroxycinnamic acid [cytoplasm] |  | <=> | AMP + diphosphate + 4-coumaroyl-CoA [cytoplasm] |
| trans-cinnamate | NADP(+) + 4-coumaroyl-CoA [cytoplasm] + H2O |  | <=> | H(+) + NADPH + oxygen + cinnamoyl-CoA [cytoplasm] |
| pinocembrin chalcone synthase | 3 malonyl-CoA + cinnamoyl-CoA [cytoplasm] |  | <=> | 3 CO2 + 4 coenzyme A + pinocembrin chalcone [cytoplasm] |
| pinocembrin chalcone isomerase | pinocembrin chalcone [cytoplasm] |  | <=> | pinocembrin [cytoplasm] |
| flavone synthase, chrysin | 2-oxoglutarate + oxygen + pinocembrin [cytoplasm] |  | <=> | CO2 + succinate + chrysin [cytoplasm] + H2O |
| chrysin transport | chrysin [cytoplasm] |  | => | chrysin [extracellular] |
| chrysin exchange | chrysin [extracellular] |  | => |  |
| **Cubebol** | | | | |
| cubebol synthase | 2-trans,6-trans-farnesyl diphosphate [cytoplasm] + H2O |  | <=> | diphosphate + cubebol [cytoplasm] |
| cubebol transport | cubebol [cytoplasm] |  | => | cubebol [extracellular] |
| cubebol exchange | cubebol [extracellular] |  | => |  |

| **Eriodictyol** | | | | |
| --- | --- | --- | --- | --- |
| tyrosine ammonia lyase | L-tyrosine [cytoplasm] |  | <=> | NH3 + p-hydroxycinnamic acid [cytoplasm] |
| cinnamate 4-hydroxylase | p-hydroxycinnamic acid [cytoplasm] |  | <=> | caffeic acid [cytoplasm] |
| 4-coumaroyl:CoA ligase, caffeic acid | ATP + coenzyme A + caffeic acid [cytoplasm] |  | <=> | AMP + diphosphate + caffeoyl-CoA [cytoplasm] |
| eriodictyol chalcone synthase | 3 malonyl-CoA + caffeoyl-CoA [cytoplasm] |  | <=> | 3 CO2 + 4 coenzyme A + eriodictyol chalcone [cytoplasm] |
| eriodictyol chalcone isomerase | eriodictyol chalcone [cytoplasm] |  | <=> | eriodictyol [cytoplasm] |
| eriodictyol transport | eriodictyol [cytoplasm] |  | => | eriodictyol [extracellular] |
| eriodictyol exchange | eriodictyol [extracellular] |  | => |  |
| **Geraniol** | | | | |
| geraniol synthase | geranyl diphosphate [cytoplasm] + H2O |  | <=> | diphosphate + geraniol [cytoplasm] |
| geraniol transport | geraniol [cytoplasm] |  | => | geraniol [extracellular] |
| geraniol exchange | geraniol [extracellular] |  | => |  |
| **Homoeriodictyol** | | | | |
| tyrosine ammonia lyase | L-tyrosine [cytoplasm] |  | => | NH3 + p-hydroxycinnamic acid [cytoplasm] |
| cinnamate 4-hydroxylase | p-hydroxycinnamic acid [cytoplasm] + H2O |  | => | caffeic acid [cytoplasm] |
| S-Adenosyl-L-methionine: 3,4-dihydroxy-trans-cinnamate 3-O-methyltransferase | S-adenosyl-L-methionine + caffeic acid [cytoplasm] |  | <=> | S-adenosyl-L-homocysteine + ferulic acid [cytoplasm] |
| Ferulate:CoA ligase (AMP-forming) | ATP + coenzyme A + ferulic acid [cytoplasm] |  | <=> | AMP + diphosphate + feruloyl-CoA [cytoplasm] |
| malonyl-CoA:feruloyl-CoA malonyltransferase (cyclizing) | 3 malonyl-CoA + feruloyl-CoA [cytoplasm] |  | <=> | 3 CO2 + 4 coenzyme A + homoeriodictyol chalcone [cytoplasm] |
| homoeriodictyol chalcone isomerase | homoeriodictyol chalcone [cytoplasm] |  | <=> | homoeriodictyol [cytoplasm] |
| homoeriodictyol transport | homoeriodictyol [cytoplasm] |  | => | homoeriodictyol [extracellular] |
| homoeriodictyol exchange | homoeriodictyol [extracellular] |  | => |  |
| **Hydrocortisone** | | | | |
| corticosterone synthesis 1a | 5,7,24(28)-ergostatrienol [cytoplasm] + H(+) + NADPH |  | <=> | NADP(+) + 5,7-ergostadienol [cytoplasm] |
| corticosterone synthesis 1b | H(+) + NADPH + 5,7-ergostadienol [cytoplasm] |  | <=> | NADP (+) + ergosta-5-enol [cytoplasm] |
| corticosterone synthesis 2 a | H(+) + NADPH + ergosta-5,7,22,24(28)-tetraen-3beta-ol [cytoplasm] |  | <=> | NADP(+) + ergosta-5-7-22-trien-3-ol [cytoplasm] |
| corticosterone synthesis 2 b | H(+) + NADPH + ergosta-5-7-22-trien-3-ol [cytoplasm] |  | <=> | NADP(+) + ergosta-5,22-diene-3-ol [cytoplasm] |
| corticosterone synthesis 3 | 3 H(+) + 3 NADPH + 3 oxygen + ergosta-5-enol [cytoplasm] +  6 reduced adrenodoxin [cytoplasm] |  | => | 3 NADP(+) + pregnenolone [cytoplasm] + 3,4-dimethylpentanal [cytoplasm] +  6 oxidized adrenodoxin [cytoplasm] + 4 H2O |
| corticosterone synthesis 4 | 3 H(+) + 3 NADPH + 3 oxygen + ergosta-5,22-diene-3-ol [cytoplasm] +  6 reduced adrenodoxin [cytoplasm] |  | => | 3 NADP(+) + pregnenolone [cytoplasm] + 3,4-dimethylpentanal [cytoplasm] +  6 oxidized adrenodoxin [cytoplasm] + 4H2O |
| 3,4-dimethylpentanal transport | 3,4-dimethylpentanal [cytoplasm] |  | => | 3,4-dimethylpentanal [extracellular] |
| 3,4-dimethylpentanal exchange | 3,4-dimethylpentanal [extracellular] |  | => |  |
| corticosterone synthesis 5 | H(+) + NADPH + oxygen + pregnenolone [cytoplasm] |  | => | NADP(+) + 17-hydroxypregnenolone [cytoplasm] + H2O |
| corticosterone synthesis 6 | H(+) + NADPH + oxygen + progesterone [cytoplasm] |  | => | NADP(+) + 17-hydroxyprogesterone [cytoplasm] + H2O |
| corticosterone synthesis 7 | NADP(+) + pregnenolone [cytoplasm] |  | <=> | H(+) + NADPH + progesterone [cytoplasm] |
| corticosterone synthesis 8 | NADP(+) + 17-hydroxypregnenolone [cytoplasm] |  | <=> | H(+) + NADPH + 17-hydroxyprogesterone [cytoplasm] |
| corticosterone synthesis 9 | H(+) + NADPH + oxygen + 17-hydroxyprogesterone [cytoplasm] |  | => | NADP(+) + 11-deoxycortisol [cytoplasm] + H2O |
| corticosterone synthesis 10 | H(+) + NADPH + oxygen + progesterone [cytoplasm] |  | => | NADP(+) + deoxycorticosterone [cytoplasm] + H2O |
| corticosterone synthesis 11 | H(+) + NADPH + oxygen + reduced adrenodoxin [cytoplasm] +  11-deoxycortisol [cytoplasm] |  | => | NADP(+) + oxidized adrenodoxin [cytoplasm] + hydrocortisone [cytoplasm] + H2O |
| corticosterone synthesis 12 | H(+) + NADPH + oxygen + reduced adrenodoxin [cytoplasm] +  deoxycorticosterone [cytoplasm] |  | => | NADP(+) + oxidized adrenodoxin [cytoplasm] + corticosterone [cytoplasm] + H2O |
| adrenodoxin oxidoreductase | NADPH + 2 oxidized adrenodoxin [cytoplasm] |  | <=> | H(+) + NADP(+) + 2 reduced adrenodoxin [cytoplasm] |
| corticosterone transport | corticosterone [cytoplasm] |  | => | corticosterone [extracellular] |
| hydrocortisone transport | hydrocortisone [cytoplasm] |  | => | hydrocortisone [extracellular] |
| corticosterone exchange | corticosterone [extracellular] |  | => |  |
| hydrocortisone exchange | hydrocortisone [extracellular] |  | => |  |
| **Lactate** | | | | |
| lactate dehydrogenase | NADH + pyruvate [cytoplasm] |  | <=> | NAD(+) + lactate [cytoplasm] |
| lactate transport | lactate [cytoplasm] |  | => | lactate [extracellular] |
| lactate exchange | lactate [extracellular] |  | => |  |
| **Luteolin** | | | | |
| tyrosine ammonia lyase | L-tyrosine [cytoplam] |  | <=> | NH3 + p-hydroxycinnamic acid [cytoplasm] |
| cinnamate 4-hydroxylase | p-hydroxycinnamic acid [cytoplasm] |  | <=> | caffeic acid [cytoplasm] |
| 4-coumaroyl:CoA ligase, caffeic acid | ATP + coenzyme A + caffeic acid [cytoplasm] |  | <=> | AMP + diphosphate + caffeoyl-CoA [cytoplasm] |
| eriodictyol chalcone synthase | 3 malonyl-CoA + caffeoyl-CoA [cytoplasm] |  | <=> | 3 CO2 + 4 coenzyme A + eriodictyol chalcone [cytoplasm] |
| eriodictyol chalcone isomerase | eriodictyol chalcone [cytoplasm] |  | <=> | eriodictyol [cytoplasm] |
| flavone synthase, luteolin | 2-oxoglutarate [cytoplasm] + oxygen + eriodictyol [cytoplasm] |  | => | CO2 + succinate [cytoplasm] + luteolin [cytoplasm] |
| luteolin transport | luteolin [cytoplasm] |  | => | luteolin [extracellular] |
| luteolin exchange | luteolin [extracellular] |  | => |  |
| **Naringenin** | | | | |
| tyrosine ammonia lyase | L-tyrosine [cytoplasm] |  | <=> | NH3 + p-hydroxycinnamic acid [cytoplasm] |
| 4-coumaroyl:CoA ligase | ATP + coenzyme A + p-hydroxycinnamic acid [cytoplasm] |  | <=> | AMP + diphosphate + 4-coumaroyl-CoA [cytoplasm] |
| naringenin chalcone synthase | 3 malonyl-CoA [cytoplasm] + 4-coumaroyl-CoA [cytoplasm] |  | <=> | 3 CO2 + 4 coenzyme A + naringenin chalcone [cytoplasm] |
| naringenin chalcone isomerase | naringenin chalcone [cytoplasm] |  | <=> | naringenin [cytoplasm] |
| naringenin transport | naringenin [cytoplasm] |  | => | naringenin [extracellular] |
| naringenin exchange | naringenin [extracellular] |  | => |  |
| **Nicotianamine** | | | | |
| nicotianamine synthase | S-adenosyl-L-methionine [cytoplasm] |  | <=> | 3 5-methylthioadenosine [cytoplasm] + nicotianamine [cytoplasm] |
| nicotianamine transport | nicotianamine [cytoplasm] |  | => | nicotianamine [extracellular] |
| nicotianamine exchange | nicotianamine [extracellular] |  | => |  |
| 5-methylthioadenosine transport | 5-methylthioadenosine [cytoplasm] |  | => | 5-methylthioadenosine [extracellular] |
| 5-methylthioadenosine exchange | 5-methylthioadenosine [extracellular] |  | => |  |
| **p-Hydroxycinnamic acid** | | | | |
| tyrosine ammonia lyase | L-tyrosine [cytoplasm] |  | <=> | NH3 + p-hydroxycinnamic acid [cytoplasm] |
| p-hydroxycinnamic acid transport | p-hydroxycinnamic acid [cytoplasm] |  | => | p-hydroxycinnamic acid [extracellular] |
| p-hydroxycinnamic acid exchange | p-hydroxycinnamic acid [extracellular] |  | => |  |
| **Patchoulol** | | | | |
| patchoulol synthase | 2-trans,6-trans-farnesyl diphosphate [cytoplasm] + H2O |  | <=> | diphosphate + patchoulol [cytoplasm] |
| patchoulol transport | patchoulol [cytoplasm] |  | => | patchoulol [extracellular] |
| patchoulol exchange | patchoulol [extracellular] |  | => |  |

| **Pinocembrin** | | | | |
| --- | --- | --- | --- | --- |
| tyrosine ammonia lyase | L-tyrosine [cytoplasm] |  | <=> | NH3 + p-hydroxycinnamic acid [cytoplasm] |
| 4-coumaroyl:CoA ligase | ATP + coenzyme A + p-hydroxycinnamic acid [cytoplasm] |  | <=> | AMP + diphosphate + 4-coumaroyl-CoA [cytoplasm] |
| trans-cinnamate | NADP(+) + 4-coumaroyl-CoA [cytoplasm] |  | <=> | H(+) + NADPH + oxygen + cinnamoyl-CoA [cytoplasm] |
| pinocembrin chalcone synthase | 3 malonyl-CoA + 4-cinnamoyl-CoA [cytoplasm] |  | => | 3 CO2 + 4 coenzyme A + pinocembrin chalcone [cytoplasm] |
| pinocembrin chalcone isomerase | pinocembrin chalcone [cytoplasm] |  | => | pinocembrin [cytoplasm] |
| pinocembrin transport | pinocembrin [cytoplasm] |  | => | pinocembrin [extracellular] |
| pinocembrin exchange | pinocembrin [extracellular] |  | => |  |
| **Poly-beta-hydroxybutyrate** | | | | |
| (R)-3-Hydroxybutanoyl-CoA:NADP+ oxidoreductase | acetoacetyl-CoA [cytoplasm] + H(+) + NADPH |  | <=> | NADP(+) + (R)-3-hydroxybutanoyl-CoA [cytoplasm] |
| poly-beta-hydroxybutyrate polymerase | (R)-3-hydroxybutanoyl-CoA [cytoplasm] |  | <=> | coenzyme A + poly-beta-hydroxybutyrate [cytoplasm] |
| poly-beta-hydroxybutyrate transport | poly-beta-hydroxybutyrate [cytoplasm] |  | => | poly-beta-hydroxybutyrate [extracellular] |
| poly-beta-hydroxybutyrate exchange | poly-beta-hydroxybutyrate [extracellular] |  | => |  |
| **Propane-1,2-diol** | | | | |
| methylglyoxal synthase | glycerone phosphate [cytoplasm] |  | => | phosphate + methylglyoxal [cytoplasm] |
| methylglyoxal reductase | H(+) + NADPH + methylglyoxal [cytoplasm] |  | <=> | NADP(+) + acetol [cytoplasm] |
| L-1,2-propanediol dehydrogenase | H(+) + NADH + acetol [cytoplasm] |  | <=> | NAD(+) + 1,2-propanediol [cytoplasm] |
| 1,2-propanediol transport | 1,2-propanediol [cytoplasm] |  | => | 1,2-propanediol [extracellular] |
| 1,2-propanediol exchange | 1,2-propanediol [extracellular] |  | => |  |
| **Propane-1,3-diol** | | | | |
| glycerol dehydratase | glycerol [cytoplasm] |  | => | 3-hydroxypropionaldehyde [cytoplasm] + H2O |
| 1,3-propanediol dehydrogenase | H(+) + NADH + 3-hydroxypropionaldehyde [cytoplasm] |  | <=> | NAD(+) + 1,3-propanediol [cytoplasm] |
| 1,3-propanediol transport | 1,3-propanediol [cytoplasm] |  | => | 1,3-propanediol [extracellular] |
| 1,3-propanediol exchange | 1,3-propanediol [extracellular] |  | => |  |
| **Resveratrol** | | | | |
| tyrosine ammonia lyase | L-tyrosine [cytoplasm] |  | <=> | NH3 + p-hydroxycinnamic acid [cytoplasm] |
| 4-coumaroyl:CoA ligase | ATP + coenzyme A + p-hydroxycinnamic acid [cytoplasm] |  | <=> | AMP + diphosphate + 4-coumaroyl-CoA [cytoplasm] |
| resveratrol synthase | 3 malonyl-CoA + 4-coumaroyl-CoA [cytoplasm] |  | <=> | 4 CO2 + 4 coenzyme A + resveratrol [cytoplasm] |
| resveratrol transport | resveratrol [cytoplasm] |  | => | resveratrol [extracellular] |
| resveratrol exchange | resveratrol [extracellular] |  | => |  |
| **Taxa-4(20),11(12)-dien-5alpha-acetoxy-10beta-ol** | | | | |
| geranylgeranyl diphosphate synthase | isopentenyl diphosphate [cytoplasm] +  2-trans,6-trans-farnesyl diphosphate [cytoplasm] |  | => | diphosphate + geranylgeranyl diphosphate [cytoplasm] |
| taxa-4(5),11(12)-diene synthase | geranylgeranyl diphosphate [cytoplasm] |  | => | diphosphate + taxa-4(5),11(12)-diene [cytoplasm] |
| taxa-4(5),11(12)-diene monooxygenase | H(+) + NADPH + oxygen + taxa-4(5),11(12)-diene [cytoplasm] |  | => | NADP(+) + taxa-4(20),11(12)-dien-5alpha-ol [cytoplasm] + H2O |
| taxa-4(20),11(12)-dien-5alpha-ol acetylase | acetyl-CoA + taxa-4(20),11(12)-dien-5alpha-ol [cytoplasm] |  | => | coenzyme A + taxa-4(20),11(12)-dien-5alpha-yl acetate [cytoplasm] |
| taxa-4(20),11(12)-dien-5alpha-yl acetate monooxygenase | H(+) + NADPH + oxygen + taxa-4(20),11(12)-dien-5alpha-yl acetate [cytoplasm] |  | => | NADP(+) + taxa-4(20),11(12)-dien-5alphaacetoxy-10beta-ol [cytoplasm] + H2O |
| taxa-4(20),11(12)-dien-5alphaacetoxy-10beta-ol transport | taxa-4(20),11(12)-dien-5alphaacetoxy-10beta-ol [cytoplasm] |  | => | taxa-4(20),11(12)-dien-5alphaacetoxy-10beta-ol [extracellular] |
| taxa-4(20),11(12)-dien-5alphaacetoxy-10beta-ol exchange | taxa-4(20),11(12)-dien-5alphaacetoxy-10beta-ol [extracellular] |  | => |  |

| **Valencene** | | | | |
| --- | --- | --- | --- | --- |
| trans,trans-farnesyl-diphosphate diphosphate-lyase [cyclizing,  (+)-Valencene-forming] | 2-trans,6-trans-farnesyl diphosphate [cytoplasm] |  | <=> | diphosphate + valencene [cytoplasm] |
| valencene transport | valencene [cytoplasm] |  | => | valencene [extracellular] |
| valencene exchange | valencene [extracellular] |  | => |  |
| **Vanillin** | | | | |
| 3-Dehydroshikimate dehydratase | 3-dehydroshikimate [cytoplasm] |  | => | protocatechuic acid [cytoplasm] + H2O |
| aromatic carboxylic acid reductase | ATP + NADPH + protocatechuic acid [cytoplasm] |  | => | AMP + NADP(+) + diphosphate + protocatechuic aldehyde [cytoplasm] |
| O-methyltransferase | S-adenosyl-L-methionine + protocatechuic aldehyde [cytoplasm] |  | => | S-adenosyl-L-homocysteine + vanillin [cytoplasm] |
| vanillin glucosyltransferase | UDP-glucose + vanillin [cytoplasm] |  | => | UDP + vanillin glucoside [cytoplasm] |
| vanillin glucoside transport | vanillin glucoside [cytoplasm] => vanillin glucoside [extracellular] |  |  |  |
| vanillin glucoside exchange | vanillin glucoside [extracellular] => |  |  |  |

Jouhten, P., Boruta, T., Andrejev, S., Pereira, F., Rocha, I., Patil, K. R., 2016. Yeast metabolic chassis designs for diverse biotechnological products. Scientific Reports. 6**,** 29694.

**Supplementary Table 2**. Reactions removed from possible reaction deletion targets.

| r_0120 | r_2204 | r_2297 | r_2444 | r_2548 | r_2628 | r_2708 | r_2788 | r_2940 | r_3030 | r_3110 | r_3190 |
| --- | --- | --- | --- | --- | --- | --- | --- | --- | --- | --- | --- |
| r_0121 | r_2205 | r_2298 | r_2445 | r_2549 | r_2629 | r_2709 | r_2789 | r_2941 | r_3031 | r_3111 | r_3191 |
| r_0122 | r_2206 | r_2299 | r_2446 | r_2550 | r_2630 | r_2710 | r_2790 | r_2942 | r_3032 | r_3112 | r_3192 |
| r_0123 | r_2207 | r_2300 | r_2447 | r_2551 | r_2631 | r_2711 | r_2791 | r_2943 | r_3033 | r_3113 | r_3193 |
| r_0124 | r_2208 | r_2368 | r_2448 | r_2552 | r_2632 | r_2712 | r_2792 | r_2944 | r_3034 | r_3114 | r_3194 |
| r_0125 | r_2209 | r_2369 | r_2449 | r_2553 | r_2633 | r_2713 | r_2793 | r_2945 | r_3035 | r_3115 | r_3195 |
| r_0281 | r_2210 | r_2370 | r_2450 | r_2554 | r_2634 | r_2714 | r_2794 | r_2946 | r_3036 | r_3116 | r_3196 |
| r_0282 | r_2211 | r_2371 | r_2451 | r_2555 | r_2635 | r_2715 | r_2795 | r_2947 | r_3037 | r_3117 | r_3197 |
| r_0283 | r_2212 | r_2372 | r_2452 | r_2556 | r_2636 | r_2716 | r_2796 | r_2948 | r_3038 | r_3118 | r_3198 |
| r_0284 | r_2213 | r_2373 | r_2453 | r_2557 | r_2637 | r_2717 | r_2797 | r_2949 | r_3039 | r_3119 | r_3199 |
| r_0285 | r_2214 | r_2374 | r_2454 | r_2558 | r_2638 | r_2718 | r_2798 | r_2950 | r_3040 | r_3120 | r_3200 |
| r_0286 | r_2215 | r_2375 | r_2455 | r_2559 | r_2639 | r_2719 | r_2799 | r_2951 | r_3041 | r_3121 | r_3201 |
| r_0287 | r_2216 | r_2376 | r_2456 | r_2560 | r_2640 | r_2720 | r_2800 | r_2952 | r_3042 | r_3122 | r_3202 |
| r_0288 | r_2217 | r_2377 | r_2457 | r_2561 | r_2641 | r_2721 | r_2801 | r_2953 | r_3043 | r_3123 | r_3203 |
| r_0289 | r_2218 | r_2378 | r_2458 | r_2562 | r_2642 | r_2722 | r_2802 | r_2954 | r_3044 | r_3124 | r_3204 |
| r_0290 | r_2232 | r_2379 | r_2459 | r_2563 | r_2643 | r_2723 | r_2803 | r_2955 | r_3045 | r_3125 | r_3205 |
| r_0291 | r_2233 | r_2380 | r_2460 | r_2564 | r_2644 | r_2724 | r_2804 | r_2956 | r_3046 | r_3126 | r_3206 |
| r_0292 | r_2234 | r_2381 | r_2461 | r_2565 | r_2645 | r_2725 | r_2805 | r_2957 | r_3047 | r_3127 | r_3207 |
| r_0293 | r_2235 | r_2382 | r_2462 | r_2566 | r_2646 | r_2726 | r_2806 | r_2958 | r_3048 | r_3128 | r_3208 |
| r_0294 | r_2236 | r_2383 | r_2463 | r_2567 | r_2647 | r_2727 | r_2807 | r_2959 | r_3049 | r_3129 | r_3209 |
| r_0295 | r_2237 | r_2384 | r_2464 | r_2568 | r_2648 | r_2728 | r_2808 | r_2960 | r_3050 | r_3130 | r_3210 |
| r_0296 | r_2238 | r_2385 | r_2465 | r_2569 | r_2649 | r_2729 | r_2809 | r_2961 | r_3051 | r_3131 | r_3211 |
| r_0297 | r_2239 | r_2386 | r_2466 | r_2570 | r_2650 | r_2730 | r_2810 | r_2962 | r_3052 | r_3132 | r_3212 |
| r_0298 | r_2240 | r_2387 | r_2467 | r_2571 | r_2651 | r_2731 | r_2811 | r_2963 | r_3053 | r_3133 | r_3213 |
| r_0299 | r_2241 | r_2388 | r_2468 | r_2572 | r_2652 | r_2732 | r_2884 | r_2964 | r_3054 | r_3134 | r_3214 |
| r_2131 | r_2242 | r_2389 | r_2469 | r_2573 | r_2653 | r_2733 | r_2885 | r_2965 | r_3055 | r_3135 | r_3215 |
| r_2140 | r_2243 | r_2390 | r_2470 | r_2574 | r_2654 | r_2734 | r_2886 | r_2966 | r_3056 | r_3136 | r_3216 |
| r_2141 | r_2244 | r_2391 | r_2471 | r_2575 | r_2655 | r_2735 | r_2887 | r_2967 | r_3057 | r_3137 | r_3217 |
| r_2142 | r_2245 | r_2392 | r_2472 | r_2576 | r_2656 | r_2736 | r_2888 | r_2968 | r_3058 | r_3138 | r_3218 |
| r_2143 | r_2246 | r_2393 | r_2473 | r_2577 | r_2657 | r_2737 | r_2889 | r_2969 | r_3059 | r_3139 | r_3219 |
| r_2144 | r_2247 | r_2394 | r_2474 | r_2578 | r_2658 | r_2738 | r_2890 | r_2970 | r_3060 | r_3140 | r_3220 |
| r_2145 | r_2248 | r_2395 | r_2475 | r_2579 | r_2659 | r_2739 | r_2891 | r_2971 | r_3061 | r_3141 | r_3221 |
| r_2146 | r_2249 | r_2396 | r_2476 | r_2580 | r_2660 | r_2740 | r_2892 | r_2972 | r_3062 | r_3142 | r_3222 |
| r_2147 | r_2250 | r_2397 | r_2477 | r_2581 | r_2661 | r_2741 | r_2893 | r_2973 | r_3063 | r_3143 | r_3223 |
| r_2148 | r_2251 | r_2398 | r_2478 | r_2582 | r_2662 | r_2742 | r_2894 | r_2974 | r_3064 | r_3144 | r_3264 |
| r_2149 | r_2252 | r_2399 | r_2479 | r_2583 | r_2663 | r_2743 | r_2895 | r_2975 | r_3065 | r_3145 | r_3265 |
| r_2150 | r_2253 | r_2400 | r_2480 | r_2584 | r_2664 | r_2744 | r_2896 | r_2976 | r_3066 | r_3146 | r_3266 |
| r_2151 | r_2254 | r_2401 | r_2481 | r_2585 | r_2665 | r_2745 | r_2897 | r_2977 | r_3067 | r_3147 | r_3267 |
| r_2152 | r_2255 | r_2402 | r_2482 | r_2586 | r_2666 | r_2746 | r_2898 | r_2978 | r_3068 | r_3148 | r_3268 |
| r_2153 | r_2256 | r_2403 | r_2483 | r_2587 | r_2667 | r_2747 | r_2899 | r_2979 | r_3069 | r_3149 | r_3269 |
| r_2154 | r_2257 | r_2404 | r_2484 | r_2588 | r_2668 | r_2748 | r_2900 | r_2980 | r_3070 | r_3150 | r_3270 |
| r_2155 | r_2258 | r_2405 | r_2485 | r_2589 | r_2669 | r_2749 | r_2901 | r_2981 | r_3071 | r_3151 | r_3271 |
| r_2156 | r_2259 | r_2406 | r_2486 | r_2590 | r_2670 | r_2750 | r_2902 | r_2982 | r_3072 | r_3152 | r_3272 |
| r_2157 | r_2260 | r_2407 | r_2487 | r_2591 | r_2671 | r_2751 | r_2903 | r_2983 | r_3073 | r_3153 | r_3273 |
| r_2158 | r_2261 | r_2408 | r_2512 | r_2592 | r_2672 | r_2752 | r_2904 | r_2984 | r_3074 | r_3154 | r_3274 |
| r_2159 | r_2262 | r_2409 | r_2513 | r_2593 | r_2673 | r_2753 | r_2905 | r_2985 | r_3075 | r_3155 | r_3275 |
| r_2160 | r_2263 | r_2410 | r_2514 | r_2594 | r_2674 | r_2754 | r_2906 | r_2986 | r_3076 | r_3156 | r_3276 |
| r_2161 | r_2264 | r_2411 | r_2515 | r_2595 | r_2675 | r_2755 | r_2907 | r_2987 | r_3077 | r_3157 | r_3277 |
| r_2162 | r_2265 | r_2412 | r_2516 | r_2596 | r_2676 | r_2756 | r_2908 | r_2988 | r_3078 | r_3158 | r_3278 |
| r_2163 | r_2266 | r_2413 | r_2517 | r_2597 | r_2677 | r_2757 | r_2909 | r_2989 | r_3079 | r_3159 | r_3279 |
| r_2164 | r_2267 | r_2414 | r_2518 | r_2598 | r_2678 | r_2758 | r_2910 | r_2990 | r_3080 | r_3160 | r_3280 |
| r_2165 | r_2268 | r_2415 | r_2519 | r_2599 | r_2679 | r_2759 | r_2911 | r_2991 | r_3081 | r_3161 | r_3281 |
| r_2166 | r_2269 | r_2416 | r_2520 | r_2600 | r_2680 | r_2760 | r_2912 | r_2992 | r_3082 | r_3162 | r_3282 |
| r_2167 | r_2270 | r_2417 | r_2521 | r_2601 | r_2681 | r_2761 | r_2913 | r_2993 | r_3083 | r_3163 | r_3283 |
| r_2168 | r_2271 | r_2418 | r_2522 | r_2602 | r_2682 | r_2762 | r_2914 | r_2994 | r_3084 | r_3164 | r_3284 |
| r_2169 | r_2272 | r_2419 | r_2523 | r_2603 | r_2683 | r_2763 | r_2915 | r_2995 | r_3085 | r_3165 | r_3285 |
| r_2170 | r_2273 | r_2420 | r_2524 | r_2604 | r_2684 | r_2764 | r_2916 | r_2996 | r_3086 | r_3166 | r_3286 |
| r_2171 | r_2274 | r_2421 | r_2525 | r_2605 | r_2685 | r_2765 | r_2917 | r_2997 | r_3087 | r_3167 | r_3287 |
| r_2172 | r_2275 | r_2422 | r_2526 | r_2606 | r_2686 | r_2766 | r_2918 | r_2998 | r_3088 | r_3168 | r_3288 |
| r_2173 | r_2276 | r_2423 | r_2527 | r_2607 | r_2687 | r_2767 | r_2919 | r_2999 | r_3089 | r_3169 | r_3289 |
| r_2174 | r_2277 | r_2424 | r_2528 | r_2608 | r_2688 | r_2768 | r_2920 | r_3000 | r_3090 | r_3170 | r_3290 |
| r_2175 | r_2278 | r_2425 | r_2529 | r_2609 | r_2689 | r_2769 | r_2921 | r_3001 | r_3091 | r_3171 | r_3291 |
| r_2176 | r_2279 | r_2426 | r_2530 | r_2610 | r_2690 | r_2770 | r_2922 | r_3002 | r_3092 | r_3172 | r_3292 |
| r_2177 | r_2280 | r_2427 | r_2531 | r_2611 | r_2691 | r_2771 | r_2923 | r_3003 | r_3093 | r_3173 | r_3293 |
| r_2178 | r_2281 | r_2428 | r_2532 | r_2612 | r_2692 | r_2772 | r_2924 | r_3004 | r_3094 | r_3174 | r_3294 |
| r_2179 | r_2282 | r_2429 | r_2533 | r_2613 | r_2693 | r_2773 | r_2925 | r_3005 | r_3095 | r_3175 | r_3295 |
| r_2180 | r_2283 | r_2430 | r_2534 | r_2614 | r_2694 | r_2774 | r_2926 | r_3006 | r_3096 | r_3176 | r_3296 |
| r_2181 | r_2284 | r_2431 | r_2535 | r_2615 | r_2695 | r_2775 | r_2927 | r_3007 | r_3097 | r_3177 | r_3297 |
| r_2182 | r_2285 | r_2432 | r_2536 | r_2616 | r_2696 | r_2776 | r_2928 | r_3008 | r_3098 | r_3178 | r_3298 |
| r_2183 | r_2286 | r_2433 | r_2537 | r_2617 | r_2697 | r_2777 | r_2929 | r_3009 | r_3099 | r_3179 | r_3299 |
| r_2194 | r_2287 | r_2434 | r_2538 | r_2618 | r_2698 | r_2778 | r_2930 | r_3010 | r_3100 | r_3180 | r_3300 |
| r_2195 | r_2288 | r_2435 | r_2539 | r_2619 | r_2699 | r_2779 | r_2931 | r_3011 | r_3101 | r_3181 | r_3301 |
| r_2196 | r_2289 | r_2436 | r_2540 | r_2620 | r_2700 | r_2780 | r_2932 | r_3022 | r_3102 | r_3182 | r_3302 |
| r_2197 | r_2290 | r_2437 | r_2541 | r_2621 | r_2701 | r_2781 | r_2933 | r_3023 | r_3103 | r_3183 | r_3303 |
| r_2198 | r_2291 | r_2438 | r_2542 | r_2622 | r_2702 | r_2782 | r_2934 | r_3024 | r_3104 | r_3184 | r_3304 |
| r_2199 | r_2292 | r_2439 | r_2543 | r_2623 | r_2703 | r_2783 | r_2935 | r_3025 | r_3105 | r_3185 | r_3305 |
| r_2200 | r_2293 | r_2440 | r_2544 | r_2624 | r_2704 | r_2784 | r_2936 | r_3026 | r_3106 | r_3186 | r_3306 |
| r_2201 | r_2294 | r_2441 | r_2545 | r_2625 | r_2705 | r_2785 | r_2937 | r_3027 | r_3107 | r_3187 | r_3307 |
| r_2202 | r_2295 | r_2442 | r_2546 | r_2626 | r_2706 | r_2786 | r_2938 | r_3028 | r_3108 | r_3188 | r_3308 |
| r_2203 | r_2296 | r_2443 | r_2547 | r_2627 | r_2707 | r_2787 | r_2939 | r_3029 | r_3109 | r_3189 | r_3309 |

| r_3310 | r_3410 | r_3490 | r_4006 | r_3695 | r_3789 | r_3869 | r_4066 | r_0263 | r_0651 | r_2845 |
| --- | --- | --- | --- | --- | --- | --- | --- | --- | --- | --- |
| r_3311 | r_3411 | r_3491 | r_4007 | r_3696 | r_3790 | r_3870 | r_4067 | r_0264 | r_0652 | r_2846 |
| r_3316 | r_3412 | r_3492 | r_4008 | r_3697 | r_3791 | r_3871 | r_4068 | r_0265 | r_0653 | r_2847 |
| r_3317 | r_3413 | r_3493 | r_4009 | r_3698 | r_3792 | r_3872 | r_4069 | r_0266 | r_0654 | r_2848 |
| r_3318 | r_3414 | r_3494 | r_4010 | r_3699 | r_3793 | r_3873 | r_4070 | r_0340 | r_0655 | r_2849 |
| r_3319 | r_3415 | r_3495 | r_4011 | r_3700 | r_3794 | r_3874 | r_4071 | r_0341 | r_0844 | r_2850 |
| r_3320 | r_3416 | r_3496 | r_4012 | r_3701 | r_3795 | r_3875 | r_4072 | r_0342 | r_0845 | r_2851 |
| r_3321 | r_3417 | r_3497 | r_4013 | r_3702 | r_3796 | r_3876 | r_4073 | r_0343 | r_0847 | r_2852 |
| r_3322 | r_3418 | r_3498 | r_4014 | r_3703 | r_3797 | r_3877 | r_4074 | r_0919 | r_0848 | r_2853 |
| r_3323 | r_3419 | r_3499 | r_4015 | r_3704 | r_3798 | r_3878 | r_4075 | r_0920 | r_0849 | r_2854 |
| r_3324 | r_3420 | r_3500 | r_4016 | r_3705 | r_3799 | r_3879 | r_4080 | r_0082 | r_0850 | r_2855 |
| r_3325 | r_3421 | r_3501 | r_4017 | r_3706 | r_3800 | r_3880 | r_4081 | r_0083 | r_1004 | r_2856 |
| r_3326 | r_3422 | r_3502 | r_4018 | r_3707 | r_3801 | r_3881 | r_4082 | r_0357 | r_1005 | r_2857 |
| r_3327 | r_3423 | r_3503 | r_4019 | r_3708 | r_3802 | r_3882 | r_4083 | r_0358 | r_1006 | r_2858 |
| r_3328 | r_3424 | r_3504 | r_4020 | r_3709 | r_3803 | r_3883 | r_4084 | r_0571 | r_1007 | r_2859 |
| r_3329 | r_3425 | r_3505 | r_4021 | r_3710 | r_3804 | r_3884 | r_4085 | r_0100 | r_2324 | r_2860 |
| r_3330 | r_3426 | r_3506 | r_4022 | r_3711 | r_3805 | r_3885 | r_4086 | r_0101 | r_2325 | r_2861 |
| r_3331 | r_3427 | r_3507 | r_4023 | r_3712 | r_3806 | r_3886 | r_4087 | r_0102 | r_2326 | r_2862 |
| r_3348 | r_3428 | r_1449 | r_4024 | r_3713 | r_3807 | r_3887 | r_4088 | r_0103 | r_2327 | r_2863 |
| r_3349 | r_3429 | r_1450 | r_4025 | r_3714 | r_3808 | r_3888 | r_4089 | r_0104 | r_2328 | r_2864 |
| r_3350 | r_3430 | r_1451 | r_4026 | r_3715 | r_3809 | r_3889 | r_4090 | r_0105 | r_2329 | r_2865 |
| r_3351 | r_3431 | r_1452 | r_4027 | r_3716 | r_3810 | r_3890 | r_4091 | r_0106 | r_2330 | r_2866 |
| r_3352 | r_3432 | r_1453 | r_4028 | r_3731 | r_3811 | r_3891 | r_4092 | r_0107 | r_2331 | r_2867 |
| r_3353 | r_3433 | r_1454 | r_4029 | r_3732 | r_3812 | r_3892 | r_4093 | r_0126 | r_2344 | r_2868 |
| r_3354 | r_3434 | r_1455 | r_4030 | r_3733 | r_3813 | r_3893 | r_4094 | r_0127 | r_2345 | r_2869 |
| r_3355 | r_3435 | r_1456 | r_4031 | r_3734 | r_3814 | r_3894 | r_4095 | r_0128 | r_2346 | r_2870 |
| r_3356 | r_3436 | r_1457 | r_4032 | r_3735 | r_3815 | r_3895 | r_4098 | r_0129 | r_2347 | r_2871 |
| r_3357 | r_3437 | r_1458 | r_4033 | r_3736 | r_3816 | r_3896 | r_4099 | r_0130 | r_2348 | r_2872 |
| r_3358 | r_3438 | r_1479 | r_4034 | r_3737 | r_3817 | r_3897 | r_4100 | r_0131 | r_2349 | r_2873 |
| r_3359 | r_3439 | r_1480 | r_4035 | r_3738 | r_3818 | r_3898 | r_4101 | r_0132 | r_2350 | r_2874 |
| r_3360 | r_3440 | r_1481 | r_4036 | r_3739 | r_3819 | r_3899 | r_4102 | r_0133 | r_2351 | r_2875 |
| r_3361 | r_3441 | r_1482 | r_4037 | r_3740 | r_3820 | r_3900 | r_4103 | r_0134 | r_2360 | r_2876 |
| r_3362 | r_3442 | r_1483 | r_3616 | r_3741 | r_3821 | r_3903 | r_4104 | r_0135 | r_2361 | r_2877 |
| r_3363 | r_3443 | r_1484 | r_3617 | r_3742 | r_3822 | r_3904 | r_4105 | r_2308 | r_2362 | r_2878 |
| r_3364 | r_3444 | r_1485 | r_3618 | r_3743 | r_3823 | r_3905 | r_4106 | r_2309 | r_2363 | r_2879 |
| r_3365 | r_3445 | r_1486 | r_3619 | r_3744 | r_3824 | r_3906 | r_4107 | r_2310 | r_2364 | r_2880 |
| r_3366 | r_3446 | r_1487 | r_3620 | r_3745 | r_3825 | r_3907 | r_4108 | r_2311 | r_2365 | r_2881 |
| r_3367 | r_3447 | r_1488 | r_3621 | r_3746 | r_3826 | r_3908 | r_4109 | r_2312 | r_2366 | r_2882 |
| r_3368 | r_3448 | r_1509 | r_3622 | r_3747 | r_3827 | r_3909 | r_4110 | r_2313 | r_2367 | r_2883 |
| r_3369 | r_3449 | r_1510 | r_3623 | r_3748 | r_3828 | r_3910 | r_4111 | r_2314 | r_2488 | r_3224 |
| r_3370 | r_3450 | r_1511 | r_3624 | r_3749 | r_3829 | r_3911 | r_4112 | r_2315 | r_2489 | r_3225 |
| r_3371 | r_3451 | r_1512 | r_3625 | r_3750 | r_3830 | r_3912 | r_4113 | r_2316 | r_2490 | r_3226 |
| r_3372 | r_3452 | r_1513 | r_3626 | r_3751 | r_3831 | r_3913 | r_4114 | r_2317 | r_2491 | r_3227 |
| r_3373 | r_3453 | r_1514 | r_3627 | r_3752 | r_3832 | r_3914 | r_4115 | r_2318 | r_2492 | r_3228 |
| r_3374 | r_3454 | r_1515 | r_3628 | r_3753 | r_3833 | r_3915 | r_4116 | r_2319 | r_2493 | r_3229 |
| r_3375 | r_3455 | r_1516 | r_3629 | r_3754 | r_3834 | r_3916 | r_4117 | r_2320 | r_2494 | r_3230 |
| r_3376 | r_3456 | r_1517 | r_3630 | r_3755 | r_3835 | r_3917 | r_4118 | r_2321 | r_2495 | r_3231 |
| r_3377 | r_3457 | r_1518 | r_3631 | r_3756 | r_3836 | r_3918 | r_4119 | r_2322 | r_2496 | r_3232 |
| r_3378 | r_3458 | r_3963 | r_3632 | r_3757 | r_3837 | r_3919 | r_4120 | r_2323 | r_2497 | r_3233 |
| r_3379 | r_3459 | r_3964 | r_3633 | r_3758 | r_3838 | r_3920 | r_4121 | r_2332 | r_2498 | r_3234 |
| r_3380 | r_3460 | r_3965 | r_3634 | r_3759 | r_3839 | r_3921 | r_4122 | r_2333 | r_2499 | r_3235 |
| r_3381 | r_3461 | r_3966 | r_3635 | r_3760 | r_3840 | r_3922 | r_4123 | r_2334 | r_2500 | r_3236 |
| r_3382 | r_3462 | r_3967 | r_3636 | r_3761 | r_3841 | r_3923 | r_4124 | r_2335 | r_2501 | r_3237 |
| r_3383 | r_3463 | r_3968 | r_3637 | r_3762 | r_3842 | r_3924 | r_4125 | r_2336 | r_2502 | r_3238 |
| r_3384 | r_3464 | r_3969 | r_3638 | r_3763 | r_3843 | r_3925 | r_4126 | r_2337 | r_2503 | r_3239 |
| r_3385 | r_3465 | r_3970 | r_3639 | r_3764 | r_3844 | r_3926 | r_4127 | r_2338 | r_2504 | r_4156 |
| r_3386 | r_3466 | r_3979 | r_3640 | r_3765 | r_3845 | r_3929 | r_4128 | r_2339 | r_2505 | r_4203 |
| r_3387 | r_3467 | r_3980 | r_3641 | r_3766 | r_3846 | r_3930 | r_4129 | r_2340 | r_2506 | r_4204 |
| r_3388 | r_3468 | r_3981 | r_3642 | r_3767 | r_3847 | r_3931 | r_4130 | r_2341 | r_2507 | r_3252 |
| r_3389 | r_3469 | r_3982 | r_3643 | r_3768 | r_3848 | r_3932 | r_4131 | r_2342 | r_2508 | r_3253 |
| r_3390 | r_3470 | r_3983 | r_3644 | r_3769 | r_3849 | r_3933 | r_4132 | r_2343 | r_2509 | r_3254 |
| r_3391 | r_3471 | r_3984 | r_3645 | r_3770 | r_3850 | r_3934 | r_4133 | r_3312 | r_2510 | r_3255 |
| r_3392 | r_3472 | r_3985 | r_3646 | r_3771 | r_3851 | r_3935 | r_4134 | r_3313 | r_2511 | r_3256 |
| r_3393 | r_3473 | r_3986 | r_3647 | r_3772 | r_3852 | r_3936 | r_4135 | r_3314 | r_2820 | r_3257 |
| r_3394 | r_3474 | r_3988 | r_3672 | r_3773 | r_3853 | r_3941 | r_4136 | r_3315 | r_2821 | r_3258 |
| r_3395 | r_3475 | r_3989 | r_3673 | r_3774 | r_3854 | r_3942 | r_4137 | r_0119 | r_2822 | r_3259 |
| r_3396 | r_3476 | r_3990 | r_3674 | r_3775 | r_3855 | r_3943 | r_4138 | r_4280 | r_2823 | r_3260 |
| r_3397 | r_3477 | r_3991 | r_3675 | r_3776 | r_3856 | r_3944 | r_4139 | r_4281 | r_2824 | r_3261 |
| r_3398 | r_3478 | r_3992 | r_3676 | r_3777 | r_3857 | r_3945 | r_4140 | r_4323 | r_2825 | r_3262 |
| r_3399 | r_3479 | r_3993 | r_3677 | r_3778 | r_3858 | r_3946 | r_4141 | r_4332 | r_2826 | r_3263 |
| r_3400 | r_3480 | r_3994 | r_3685 | r_3779 | r_3859 | r_3947 | r_4142 | r_0399 | r_2827 |  |
| r_3401 | r_3481 | r_3995 | r_3686 | r_3780 | r_3860 | r_3948 | r_4143 | r_0400 | r_2836 |  |
| r_3402 | r_3482 | r_3997 | r_3687 | r_3781 | r_3861 | r_3949 | r_4144 | r_0402 | r_2837 |  |
| r_3403 | r_3483 | r_3998 | r_3688 | r_3782 | r_3862 | r_3950 | r_4145 | r_0410 | r_2838 |  |
| r_3404 | r_3484 | r_3999 | r_3689 | r_3783 | r_3863 | r_3951 | r_4146 | r_0412 | r_2839 |  |
| r_3405 | r_3485 | r_4000 | r_3690 | r_3784 | r_3864 | r_3952 | r_4147 | r_0646 | r_2840 |  |
| r_3406 | r_3486 | r_4001 | r_3691 | r_3785 | r_3865 | r_3953 | r_4148 | r_0647 | r_2841 |  |
| r_3407 | r_3487 | r_4002 | r_3692 | r_3786 | r_3866 | r_3954 | r_4149 | r_0648 | r_2842 |  |
| r_3408 | r_3488 | r_4003 | r_3693 | r_3787 | r_3867 | r_3955 | r_4150 | r_0649 | r_2843 |  |
| r_3409 | r_3489 | r_4004 | r_3694 | r_3788 | r_3868 | r_3956 | r_4151 | r_0650 | r_2844 |  |

**Supplementary Table 3.** Possible compounds for chemical environment nutrients.

| compound name | Id for its uptake reaction |
| --- | --- |
| 2-oxoglutarate | r_1586 |
| acetate | r_1634 |
| ammonium | r_1654 |
| choline | r_1683 |
| D-galactose | r_1710 |
| D-glucose | r_1714 |
| ethanol | r_1761 |
| fumarate | r_1798 |
| gamma-aminobutyrate | r_1800 |
| glycerol | r_1808 |
| L-alanine | r_1873 |
| L-arginine | r_1879 |
| L-asparagine | r_1880 |
| L-aspartate | r_1881 |
| L-aspartate | r_1883 |
| L-glucitol | r_1886 |
| L-glutamate | r_1889 |
| L-glutamine | r_1891 |
| L-glycine | r_1810 |
| L-histidine | r_1893 |
| L-isoleucine | r_1897 |
| L-leucine | r_1899 |
| L-lysine | r_1900 |
| L-methionine | r_1902 |
| L-phenylalanine | r_1903 |
| L-proline | r_1904 |
| L-serine | r_1906 |
| L-threonine | r_1911 |
| L-tryptophan | r_1912 |
| L-tyrosine | r_1913 |
| L-valine | r_1914 |
| ornithine | r_1987 |
| palmitate | r_1993 |
| pyruvate | r_2033 |
| spermidine | r_2051 |
| spermine | r_2052 |
| succinate | r_2056 |

**Supplementary Table 4**. Darwinian selection strategies for synthetic pathways.

| **4-Methyltiobutyl-desulfoglucosinolate pathway** | | | |
| --- | --- | --- | --- |
| **EvolveX score** | 131,52 mmol/g CDW |  | |
|  | **compound name** |  | |
| **Evolution environment** | ethanol |  |  |
|  | methionine |  |  |
|  | palmitate |  |  |
|  | **reaction name** | **reaction** | **gene(s) encoding  for the enzyme(s)  catalyzing the reaction** |
| **Deleted reactions** | 3-deoxy-D-arabino-heptulosonate 7-phosphate synthetase | D-erythrose 4-phosphate + H2O + phosphoenolpyruvate -->  7-phospho-2-dehydro-3-deoxy-D-arabino-heptonic acid + phosphate | *ARO3*, ARO*4* |
|  | acetyl-CoA synthetase | acetate + ATP + coenzyme A --> acetyl-CoA + AMP + diphosphate | *ACS1*, *ACS2* |
|  | ATP:D-glucose 6-phosphotransferase | ATP + D-glucose --> ADP + D-glucose 6-phosphate | *EMI2* |
|  | O4-succinyl-L-homoserine hydrogen sulfide S-(3-amino-3-carboxypropyl)transferase  O-succinyl-L-homoserine succinate-lyase | hydrogen sulfide + O-succinyl-L-homoserine <=> L-homocysteine + succinate | *STR2*, *YLL058W*, *YML082W* |
| **Apigenin pathway** | | | |
| **EvolveX score** | 46,1 mmol/g CDW |  | |
|  | **compound name** |  | |
| **Evolution environment** | glycerol |  |  |
|  | tyrosine |  |  |
|  | palmitate |  |  |
|  | **reaction name** | **reaction** | **gene(s) encoding  for the enzyme(s)  catalyzing the reaction** |
| **Deleted reactions** | 3',5'-bisphosphate nucleotidase | adenosine 3',5'-bismonophosphate + H2O --> AMP + phosphate | *MET22* |
|  | enolase | 2-phospho-D-glyceric acid <=> H2O + phosphoenolpyruvate | *ERR1*, *ERR2*, *ERR3*, *ENO1*, *ENO2* |
|  | formate-tetrahydrofolate ligase | ATP + formate + THF <=> 10-formyl-THF + ADP + phosphate | *ADE3* |
|  | 5-formethyltetrahydrofolate cyclo-ligase | 5-formyltetrahydrofolic acid + ATP --> 5,10-methenyl-THF + ADP + phosphate | *FAU1* |
| **Chrysin pathway** | | | |
| **EvolveX score** | 53,92 mmol/g CDW |  | |
|  | **compound name** |  | |
| **Evolution environment** | glycerol |  |  |
|  | tyrosine |  |  |
|  | palmitate |  |  |
|  | **reaction name** | **reaction** | **gene(s) encoding  for the enzyme(s)  catalyzing the reaction** |
| **Deleted reactions** | 3',5'-bisphosphate nucleotidase | adenosine 3',5'-bismonophosphate + H2O --> AMP [cytoplasm] + phosphate [cytoplasm] | *MET22* |
|  | enolase | 2-phospho-D-glyceric acid [cytoplasm] <=> H2O + phosphoenolpyruvate | *ERR1*, *ERR2*, *ERR3*, *ENO1*, *ENO2* |
|  | phenylalanine transaminase | 2-oxoglutarate + L-phenylalanine <=> keto-phenylpyruvate + L-glutamate | *ARO8* |
|  | phenylalanine transaminase | L-phenylalanine + pyruvate <=> keto-phenylpyruvate + L-alanine | *ARO9* |

| **Eriodictyol pathway** | | | |
| --- | --- | --- | --- |
| **EvolveX score** | 46,1 mmol/g CDW |  | |
|  | **compound name** |  | |
| **Evolution environment** | glycerol |  |  |
|  | tyrosine |  |  |
|  | palmitate |  |  |
|  | **reaction name** | **reaction** | **gene(s) encoding  for the enzyme(s)  catalyzing the reaction** |
| **Deleted reactions** | 3',5'-bisphosphate nucleotidase | adenosine 3',5'-bismonophosphate + H2O --> AMP + phosphate | *MET22* |
|  | glutamate dehydrogenase (NADP) | 2-oxoglutarate + ammonium + H+ + NADPH --> H2O + L-glutamate + NADP (+) | *GDH1*, GDH*3* |
|  | phosphoglycerate mutase | 3-phosphonato-D-glycerate (3-) <=> 2-phospho-D-glyceric acid | *GPM1*, *YOR283W* |
|  | UTP-glucose-1-phosphate uridylyltransferase | D-glucose 1-phosphate + H+ + UTP <=> diphosphate + UDP-D-glucose | *UGP1*, *YHL012W* |
| **Homoeriodictyol pathway** | | | |
| **EvolveX score** | 52,69 mmol/g CDW |  | |
|  | **compound name** |  | |
| **Evolution environment** | glycerol |  |  |
|  | tyrosine |  |  |
|  | palmitate |  |  |
|  | **reaction name** | **reaction** | **gene(s) encoding  for the enzyme(s)  catalyzing the reaction** |
| **Deleted reactions** | 3',5'-bisphosphate nucleotidase | adenosine 3',5'-bismonophosphate + H2O --> AMP + phosphate | *MET22* |
|  | indole-3-pyruvate decarboxylase | H+ + indole-3-pyruvate --> carbon dioxide + indol-3-ylacetaldehyde | *PDC1*, *PDC5*, *PDC6* |
|  | ornithine transaminase | 2-oxoglutarate + ornithine --> L-glutamate + L-glutamic 5-semialdehyde | *CAR2* |
|  | phosphoglycerate kinase | 1,3-bisphospho-D-glycerate + ADP <=> 3-phosphonato-D-glycerate (3-) + ATP | *PGK1* |
| **Lactate pathway** | | | |
| **EvolveX score** | 48,66 mmol/g CDW |  | |
|  | **compound name** |  | |
| **Evolution environment** | choline |  |  |
|  | D-glucose |  |  |
|  | methionine |  |  |
|  | **reaction name** | **reaction** | **gene(s) encoding  for the enzyme(s)  catalyzing the reaction** |
| **Deleted reactions** | fumarase | fumarate + H2O <=> (S)-malate | *FUM1* |
|  | fumarase, cytoplasmic | fumarate + H2O <=> (S)-malate | *FUM1* |
|  | glucose-6-phosphate isomerase | D-glucose 6-phosphate <=> D-fructose 6-phosphate | *PGI1* |
|  | glyceraldehyde-3-phosphate dehydrogenase | glyceraldehyde 3-phosphate + NAD + phosphate <=> 1,3-bisphospho-D-glycerate + H+ + NADH | *TDH1*, *TDH2*, *TDH3* |

| **Luteolin pathway** | | | |
| --- | --- | --- | --- |
| **EvolveX score** | 52,69 mmol/g CDW |  | |
|  | **compound name** |  | |
| **Evolution environment** | glycerol |  |  |
|  | tyrosine |  |  |
|  | palmitate |  |  |
|  | **reaction name** | **reaction** | **gene(s) encoding  for the enzyme(s)  catalyzing the reaction** |
| **Deleted reactions** | 3',5'-bisphosphate nucleotidase | adenosine 3',5'-bismonophosphate + H2O --> AMP + phosphate | *MET22* |
|  | dihydroneopterin monophosphate dephosphorylase | 7,8-dihydroneopterin 3'-phosphate + H2O --> 7,8-dihydroneopterin + phosphate | *GET3* |
|  | glycerol-3-phosphate dehydrogenase (NAD) | dihydroxyacetone phosphate + H+ + NADH --> glycerol 3-phosphate + NAD | *GPD1*, *GPD2* |
|  | phosphoglycerate mutase | 3-phosphonato-D-glycerate (3-) <=> 2-phospho-D-glyceric acid | *YOR283W*, *GPM1* |
| **Naringenin pathway** | | | |
| **EvolveX score** | 39,52 mmol/g CDW |  | |
|  | **compound name** |  | |
| **Evolution environment** | glycerol |  |  |
|  | tyrosine |  |  |
|  | palmitate |  |  |
|  | **reaction name** | **reaction** | **gene(s) encoding  for the enzyme(s)  catalyzing the reaction** |
| **Deleted reactions** | 3',5'-bisphosphate nucleotidase | adenosine 3',5'-bismonophosphate + H2O --> AMP + phosphate | *MET22* |
|  | alcohol acetyltransferase (phenylethanol alcohol) | 2-phenylethanol + acetyl-CoA --> coenzyme A + phenethyl acetate | *ATF1*, *ATF2* |
|  | ornithine transaminase | 2-oxoglutarate + ornithine --> L-glutamate + L-glutamic 5-semialdehyde | *CAR2* |
|  | phosphoenolpyruvate carboxykinase | ATP + oxaloacetate --> ADP + carbon dioxide + phosphoenolpyruvate | *PCK1* |
| **p-Hydroxycinnamic acid pathway** | | | |
| **EvolveX score** | 19,76 mmol/g CDW |  | |
|  | **compound name** |  | |
| **Evolution environment** | fumarate |  |  |
|  | tyrosine |  |  |
|  | palmitate |  |  |
|  | **reaction name** | **reaction** | **gene(s) encoding  for the enzyme(s)  catalyzing the reaction** |
| **Deleted reactions** | 3',5'-bisphosphate nucleotidase | adenosine 3',5'-bismonophosphate + H2O --> AMP + phosphate | *MET22* |
|  | glycerol-3-phosphate dehydrogenase (fad) | FAD + glycerol 3-phosphate --> dihydroxyacetone phosphate + FADH2 | *GUT2* |
|  | malate dehydrogenase, peroxisomal | (S)-malate + NAD <=> H+ + NADH + oxaloacetate | *MDH3* |

| **Pinocembrin pathway** | | | |
| --- | --- | --- | --- |
| **EvolveX score** | 46,1 mmol/g CDW |  | |
|  | **compound name** |  | |
| **Evolution environment** | glycerol |  |  |
|  | tyrosine |  |  |
|  | palmitate |  |  |
|  | **reaction name** | **reaction** | **gene(s) encoding  for the enzyme(s)  catalyzing the reaction** |
| **Deleted reactions** | 3',5'-bisphosphate nucleotidase | adenosine 3',5'-bismonophosphate + H2O --> AMP + phosphate | *MET22* |
|  | dTMP kinase | ATP + dTMP --> ADP + dTDP | *CDC8* |
|  | mevalonate kinase (atp) | (R)-mevalonate + ATP --> (R)-5-phosphomevalonic acid + ADP + H+ | *ERG12* |
|  | phosphoglycerate kinase | 1,3-bisphospho-D-glycerate + ADP <=> 3-phosphonato-D-glycerate (3-) + ATP | *PGK1* |
| **Resveratrol pathway** | | | |
| **EvolveX score** | 32,93 mmol/g CDW |  | |
|  | **compound name** |  | |
| **Evolution environment** | glycerol |  |  |
|  | tyrosine |  |  |
|  | palmitate |  |  |
|  | **reaction name** | **reaction** | **gene(s) encoding  for the enzyme(s)  catalyzing the reaction** |
| **Deleted reactions** | 3',5'-bisphosphate nucleotidase | adenosine 3',5'-bismonophosphate + H2O --> AMP + phosphate | *MET22* |
|  | mevalonate kinase (atp) | (R)-mevalonate + ATP --> (R)-5-phosphomevalonic acid + ADP + H+ | *ERG12* |
|  | mevalonate kinase (ctp) | (R)-mevalonate + CTP --> (R)-5-phosphomevalonic acid + CDP + H+ | *ERG12* |
|  | phosphoglycerate mutase | 3-phosphonato-D-glycerate (3-) <=> 2-phospho-D-glyceric acid | *YOR283W*, *GPM1* |
| **Vanillin pathway** | | | |
| **EvolveX score** | 20,66 mmol/g CDW |  | |
|  | **compound name** |  | |
| **Evolution environment** | D-galactose |  |  |
|  | methionine |  |  |
|  | tyrosine |  |  |
|  | **reaction name** | **reaction** | **gene(s) encoding  for the enzyme(s)  catalyzing the reaction** |
| **Deleted reactions** | acetyl-CoA synthetase | acetate + ATP + coenzyme A --> acetyl-CoA + AMP + diphosphate | *ACS1*, *ACS2* |
|  | phosphogluconate dehydrogenase | 6-phospho-D-gluconate + NADP (+) --> carbon dioxide + D-ribulose 5-phosphate + NADPH | *GND1*, *GND2* |
|  | pyruvate carboxylase | ATP + bicarbonate + pyruvate --> ADP + H+ + oxaloacetate + phosphate | *PYC1*, *PYC2* |
|  | transketolase 1 | D-xylulose 5-phosphate + ribose-5-phosphate <=> glyceraldehyde 3-phosphate + sedoheptulose 7-phosphate | *TKL1*, *TKL2* |

**Supplementary Table 5**. Darwinian selection strategies for native flux targets.

| **6-Methylsalicylate pathway** | | | |
| --- | --- | --- | --- |
| **EvolveX score** | 948.71 mmol/g CDW |  | |
|  | **compound name** |  | |
| **Evolution environment** | 2-oxoglutarate |  |  |
|  | acetate |  |  |
|  | methionine |  |  |
|  | **reaction name** | **reaction** | **gene(s) encoding for the enzyme(s) catalyzing the reaction** |
| **Deleted reactions** | 6-phosphogluconolactonase | 6-O-phosphono-D-glucono-1,5-lactone + H2O --> 6-phospho-D-gluconate + H+ | *SOL3*, *SOL4* |
|  | glutamate decarboxylase | H+ + L-glutamate --> carbon dioxide + gamma-aminobutyrate | *GAD1* |
|  | malate synthase | acetyl-CoA + glyoxylate + H2O --> (S)-malate + coenzyme A + H+ | *DAL7*, *MLS1* |
|  | oxoglutarate dehydrogenase (lipoamide) | 2-oxoglutarate + H+ + lipoamide --> carbon dioxide + S(8)-suc+cinyldihydrolipoamide | *KGD1*, *KGD2*, *LPD1* |
| **8-Epi-cedrol pathway** | | | |
| **EvolveX score** | 1035.86 mmol/g CDW |  | |
|  | **compound name** |  | |
| **Evolution environment** | 2-oxoglutarate |  |  |
|  | choline |  |  |
|  | methionine |  |  |
|  | **reaction name** | **reaction** | **gene(s) encoding for the enzyme(s) catalyzing the reaction** |
| **Deleted reactions** | glutamate decarboxylase | H+ + L-glutamate --> carbon dioxide + gamma-aminobutyrate | *GAD1* |
|  | malate synthase | acetyl-CoA + glyoxylate + H2O --> (S)-malate + coenzyme A + H+ | *DAL7*, *MLS1* |
|  | oxoglutarate dehydrogenase  (dihydrolipoamide S-succinyltransferase) | coenzyme A + S(8)-succinyldihydrolipoamide --> dihydrolipoamide + succinyl-CoA | *KGD1, KGD2*, *LPD1* |
|  | phosphogluconate dehydrogenase | 6-phospho-D-gluconate + NADP (+) --> carbon dioxide + D-ribulose 5-phosphate + NADPH | *GND1*, *GND2* |
| **Amorpha-4-11-diene pathway** | | | |
| **EvolveX score** | 1035.86 mmol/g CDW |  | |
|  | **compound name** |  | |
| **Evolution environment** | 2-oxoglutarate |  |  |
|  | methionine |  |  |
|  | pyruvate |  |  |
|  | **reaction name** | **reaction** | **gene(s) encoding for the enzyme(s) catalyzing the reaction** |
| **Deleted reactions** | glutamate decarboxylase | H+ + L-glutamate --> carbon dioxide + gamma-aminobutyrate | *GAD1* |
|  | malate synthase | acetyl-CoA + glyoxylate + H2O --> (S)-malate + coenzyme A + H+ | *DAL7*, *MLS1* |
|  | oxoglutarate dehydrogenase (lipoamide) | 2-oxoglutarate + H+ + lipoamide --> carbon dioxide + S(8)-succinyldihydrolipoamide | *KGD1*, *KGD2*, *LPD1* |
|  | phosphogluconate dehydrogenase | 6-phospho-D-gluconate + NADP (+) --> carbon dioxide + D-ribulose 5-phosphate + NADPH | *GND1*, *GND2* |

| **Artemisinic acid pathway** | | | |
| --- | --- | --- | --- |
| **EvolveX score** | 1034.31 mmol/g CDW |  | |
|  | **compound name** |  | |
| **Evolution environment** | 2-oxoglutarate |  |  |
|  | acetate |  |  |
|  | methionine |  |  |
|  | **reaction name** | **reaction** | **gene(s) encoding for the enzyme(s) catalyzing the reaction** |
| **Deleted reactions** | glutamate decarboxylase | H+ + L-glutamate --> carbon dioxide + gamma-aminobutyrate | *GAD1* |
|  | malate synthase | acetyl-CoA + glyoxylate + H2O --> (S)-malate + coenzyme A + H+ | *DAL7*, *MLS1* |
|  | oxoglutarate dehydrogenase (lipoamide) | 2-oxoglutarate + H+ + lipoamide --> carbon dioxide + S(8)-succinyldihydrolipoamide | *KGD1*, *KGD2*, *LPD1* |
|  | phosphogluconate dehydrogenase | 6-phospho-D-gluconate + NADP(+) --> carbon dioxide + D-ribulose 5-phosphate + NADPH | *GND1*, *GND2* |
| **Beta carotene** | | | |
| **EvolveX score** | 846.54 mmol/g CDW |  | |
|  | **compound name** |  | |
| **Evolution environment** | methionine |  |  |
|  | palmitate |  |  |
|  | spermidine |  |  |
|  | **reaction name** | **reaction** | **gene(s) encoding for the enzyme(s) catalyzing the reaction** |
| **Deleted reactions** | catalase | 2.0 hydrogen peroxide --> 2.0 H2O + oxygen | *CTA1* |
|  | glucose 6-phosphate dehydrogenase | D-glucose 6-phosphate + NADP(+) --> 6-O-phosphono-D-glucono-1,5-lactone + H+ + NADPH | *ZWF1* |
|  | glutamate decarboxylase | H+ + L-glutamate --> carbon dioxide + gamma-aminobutyrate | *GAD1* |
|  | isocitrate dehydrogenase (NADP) | isocitrate + NADP(+) <=> 2-oxoglutarate + carbon dioxide + NADPH | *IDP2* |
| **Butanol pathway** | | | |
| **EvolveX score** | 401.73 mmol/g CDW |  | |
|  | **compound name** |  | |
| **Evolution environment** | choline |  |  |
|  | ethanol |  |  |
|  | methionine |  |  |
|  | **reaction name** | **reaction** | **gene(s) encoding for the enzyme(s) catalyzing the reaction** |
| **Deleted reactions** | isocitrate dehydrogenase (NADP) | isocitrate + NADP(+) <=> 2-oxoglutarate + carbon dioxide + NADPH | *IDP2* |
|  | malate dehydrogenase | (S)-malate + NAD <=> H+ + NADH + oxaloacetate | *MDH1* |
|  | nucleoside diphosphate kinase | ATP + CDP --> ADP + CTP | *YNK1* |
|  | sulfate adenylyltransferase (ADP) | ADP + H+ + sulphate --> 5'-adenylyl sulfate + phosphate | *APA1* |

| **Cubebol pathway** | | | |
| --- | --- | --- | --- |
| **EvolveX score** | 1035.86 mmol/g CDW |  | |
|  | **compound name** |  | |
| **Evolution environment** | 2-oxoglutarate |  |  |
|  | choline |  |  |
|  | methionine |  |  |
|  | **reaction name** | **reaction** | **gene(s) encoding for the enzyme(s) catalyzing the reaction** |
| **Deleted reactions** | glutamate decarboxylase | H+ + L-glutamate --> carbon dioxide + gamma-aminobutyrate | *GAD1* |
|  | malate synthase | acetyl-CoA + glyoxylate + H2O --> (S)-malate + coenzyme A + H+ | *DAL7*, *MLS1* |
|  | oxoglutarate dehydrogenase (lipoamide) | 2-oxoglutarate + H+ + lipoamide --> carbon dioxide + S(8)-succinyldihydrolipoamide | *KGD1*, *KGD2*, *LPD1* |
|  | phosphogluconate dehydrogenase | 6-phospho-D-gluconate + NADP (+) --> carbon dioxide + D-ribulose 5-phosphate + NADPH | *GND1*, *GND2* |
| **Geraniol pathway** | | | |
| **EvolveX score** | 1037.33 mmol/g CDW |  | |
|  | **compound name** |  | |
| **Evolution environment** | 2-oxoglutarate |  |  |
|  | acetate |  |  |
|  | methionine |  |  |
|  | **reaction name** | **reaction** | **gene(s) encoding for the enzyme(s) catalyzing the reaction** |
| **Deleted reactions** | glutamate decarboxylase | H+ + L-glutamate --> carbon dioxide + gamma-aminobutyrate | *GAD1* |
|  | glycine-cleavage complex (lipoamide) | dihydrolipoamide + NAD --> H+ + lipoamide + NADH | *GCV1*, *GCV3* |
|  | malate synthase | acetyl-CoA + glyoxylate + H2O --> (S)-malate + coenzyme A + H+ | *DAL7*, *MLS1* |
|  | phosphogluconate dehydrogenase | 6-phospho-D-gluconate + NADP (+) --> carbon dioxide + D-ribulose 5-phosphate + NADPH | *GND1*, *GND2*, *LPD1*, *GCV2*, *KGD1*, *KGD2* |
| **Hydrocortisone pathway** | | | |
| **EvolveX score** | 1064.52 mmol/g CDW |  | |
|  | **compound name** |  | |
| **Evolution environment** | 2-oxoglutarate |  |  |
|  | L-glucitol |  |  |
|  | methionine |  |  |
|  | **reaction name** | **reaction** | **gene(s) encoding for the enzyme(s) catalyzing the reaction** |
| **Deleted reactions** | glucose 6-phosphate dehydrogenase | D-glucose 6-phosphate + NADP(+) --> 6-O-phosphono-D-glucono-1,5-lactone + H+ + NADPH | *ZWF1* |
|  | glutamate decarboxylase | H+ + L-glutamate --> carbon dioxide + gamma-aminobutyrate | *GAD1* |
|  | glycine-cleavage complex (lipoamide) | dihydrolipoamide + NAD --> H+ + lipoamide + NADH | *GCV1*, *GCV3* |
|  | malate synthase | acetyl-CoA + glyoxylate + H2O --> (S)-malate + coenzyme A + H+ | *DAL7*, *MLS1* |

| **Nicotianamine pathway** | | | |
| --- | --- | --- | --- |
| **EvolveX score** | 459.93 mmol/g CDW |  | |
|  | **compound name** |  | |
| **Evolution environment** | glycine |  |  |
|  | valine |  |  |
|  | spermidine |  |  |
|  | **reaction name** | **reaction** | **gene(s) encoding for the enzyme(s) catalyzing the reaction** |
| **Deleted reactions** | isocitrate dehydrogenase (NAD+) | isocitrate + NAD --> 2-oxoglutarate + carbon dioxide + NADH | *IDH1*, *IDH2* |
|  | pyruvate decarboxylase | H+ + pyruvate --> acetaldehyde + carbon dioxide | *PDC1*, *PDC5*, *PDC6* |
|  | pyruvate dehydrogenase | coenzyme A + NAD + pyruvate --> acetyl-CoA + carbon dioxide + NADH | *PDB1*, *PDA1*, *LPD1*, *PDX1*, *LAT1* |
|  | fumarate reductase (FMN) | FMNH2 + fumarate <=> FMN + succinate | *FRD1* |
| **Patchoulol pathway** | | | |
| **EvolveX score** | 1035.86 mmol/g CDW |  | |
|  | **compound name** |  | |
| **Evolution environment** | 2-oxoglutarate |  |  |
|  | choline |  |  |
|  | methionine |  |  |
|  | **reaction name** | **reaction** | **gene(s) encoding for the enzyme(s) catalyzing the reaction** |
| **Deleted reactions** | glutamate decarboxylase | H+ + L-glutamate --> carbon dioxide + gamma-aminobutyrate | *GAD1* |
|  | malate synthase | acetyl-CoA + glyoxylate + H2O --> (S)-malate + coenzyme A + H+ | *DAL7*, *MLS1* |
|  | oxoglutarate dehydrogenase (lipoamide) | 2-oxoglutarate + H+ + lipoamide --> carbon dioxide + S(8)-succinyldihydrolipoamide | *KGD1*, *KGD2*, *LPD1* |
|  | phosphogluconate dehydrogenase | 6-phospho-D-gluconate + NADP(+) --> carbon dioxide + D-ribulose 5-phosphate + NADPH | *GND1*, *GND2* |
| **Poly-beta-hydroxybutyrate** | | | |
| **EvolveX score** | 696.21 mmol/g CDW |  | |
|  | **compound name** |  | |
| **Evolution environment** | choline |  |  |
|  | ethanol |  |  |
|  | methionine |  |  |
|  | **reaction name** | **reaction** | **gene(s) encoding for the enzyme(s) catalyzing the reaction** |
| **Deleted reactions** | 3-methyl-2-oxopentanoate decarboxylase | (S)-3-methyl-2-oxopentanoate + H+ --> 2-methylbutanal + carbon dioxide | *THI3*, *PDC1*, *PDC5*, *PDC6* |
|  | isocitrate dehydrogenase (NADP) | isocitrate + NADP(+) <=> 2-oxoglutarate + carbon dioxide + NADPH | *IDP2* |
|  | malate synthase | acetyl-CoA + glyoxylate + H2O --> (S)-malate + coenzyme A + H+ | *DAL7*, *MLS1* |
|  | sulfate adenylyltransferase (ADP) | ADP + H+ + sulphate --> 5'-adenylyl sulfate + phosphate | *APA1* |

| **Propane-1,2-diol** | | | |
| --- | --- | --- | --- |
| **EvolveX score** | 88.39 mmol/g CDW |  | |
|  | **compound name** |  | |
| **Evolution environment** | fumarate |  |  |
|  | cysteine |  |  |
|  | glucitol |  |  |
|  | **reaction name** | **reaction** | **gene(s) encoding for the enzyme(s) catalyzing the reaction** |
| **Deleted reactions** | 6-phosphogluconolactonase | 6-O-phosphono-D-glucono-1,5-lactone + H2O --> 6-phospho-D-gluconate + H+ | *SOL3*, *SOL4* |
|  | mitochondrial alcohol dehydrogenase | acetaldehyde + H+ + NADH --> ethanol + NAD | *ADH4* |
|  | fumarase, cytoplasmic | fumarate + H2O <=> (S)-malate | *FUM1* |
|  | phosphoserine transaminase | 3-phospho-hydroxypyruvate + L-glutamate --> 2-oxoglutarate + 3-phospho-serine | *SER1* |
| **Propane-1,3-diol** | | | |
| **EvolveX score** | 438.12 mmol/g CDW |  | |
|  | **compound name** |  | |
| **Evolution environment** | ammonium |  |  |
|  | ornithine |  |  |
|  | spermidine |  |  |
|  | **reaction name** | **reaction** | **gene(s) encoding for the enzyme(s) catalyzing the reaction** |
| **Deleted reactions** | alanine glyoxylate aminotransferase | glyoxylate + L-alanine --> L-glycine + pyruvate | *AGX1* |
|  | homoserine dehydrogenase (NADH) | H+ + L-aspartate 4-semialdehyde + NADH --> L-homoserine + NAD | *HOM6* |
|  | isoleucine transaminase | 2-oxoglutarate + L-isoleucine <=> (S)-3-methyl-2-oxopentanoate + L-glutamate | *BAT1* |
|  | L-alanine transaminase | 2-oxoglutarate + L-alanine <=> L-glutamate + pyruvate | *ALT1* |
| **Taxa-4(20),11(12)-dien-5alpha-acetoxy-10beta-ol** | | | |
| **EvolveX score** | 1034.31 mmol/g CDW |  | |
|  | **compound name** |  | |
| **Evolution environment** | 2-oxoglutarate |  |  |
|  | glucitol |  |  |
|  | methionine |  |  |
|  | **reaction name** | **reaction** | **gene(s) encoding for the enzyme(s) catalyzing the reaction** |
| **Deleted reactions** | glucose 6-phosphate dehydrogenase | D-glucose 6-phosphate + NADP(+) --> 6-O-phosphono-D-glucono-1,5-lactone + H+ + NADPH | *ZWF1* |
|  | glutamate decarboxylase | H+ + L-glutamate --> carbon dioxide + gamma-aminobutyrate | *GAD1* |
|  | glycine-cleavage complex (lipoamide) | dihydrolipoamide + NAD --> H+ + lipoamide + NADH | *GCV1*, *GCV3* |
|  | malate synthase | acetyl-CoA + glyoxylate + H2O --> (S)-malate + coenzyme A + H+ | *DAL7*, *MLS1* |

| **Valencene** | | | |
| --- | --- | --- | --- |
| **EvolveX score** | 1035.86 mmol/g CDW |  | |
|  |  |  | |
| **Evolution environment** | 2-oxoglutarate |  |  |
|  | methionine |  |  |
|  | pyruvate |  |  |
|  | **reaction name** | **reaction** | **gene(s) encoding for the enzyme(s) catalyzing the reaction** |
| **Deleted reactions** | glutamate decarboxylase | H+ + L-glutamate --> carbon dioxide + gamma-aminobutyrate | *GAD1* |
|  | malate synthase | acetyl-CoA + glyoxylate + H2O --> (S)-malate + coenzyme A + H+ | *DAL7*, *MLS1* |
|  | oxoglutarate dehydrogenase (dihydrolipoamide S-succinyltransferase) | coenzyme A + S(8)-succinyldihydrolipoamide --> dihydrolipoamide + succinyl-CoA | *KGD1*, *KGD2*, *LPD1* |
|  | phosphogluconate dehydrogenase | 6-phospho-D-gluconate + NADP(+) --> carbon dioxide + D-ribulose 5-phosphate + NADPH | *GND1*, *GND2* |

**Supplementary Table 6**. Oxalate pathway.

| **reaction name** | **reaction** |
| --- | --- |
| Oxaloacetase | oxaloacetate [cytoplasm] + H2O [cytoplasm] --> acetate [cytoplasm] + oxalate [cytoplasm] |
| Oxalate-CoA ligase | oxalate [cytoplasm] + CoA [cytplasm] + ATP [cytoplasm --> oxalyl-CoA [cytoplasm] + AMP [cytoplasm] + diphosphate [cytoplasm] |
| Glyoxylate dehydrogenase | oxalyl-CoA [cytoplasm] + NADPH [cytoplasm] + H+ [cytoplasm] --> CoA [cytoplasm] + glyoxylate [cytoplasm] NADP(+) [cytoplasm] |
| Glyoxylate reductase | glyoxylate [cytoplasm] + NADPH [cytoplasm] + H+ [cytoplasm] --> glycolate [cytoplasm] + NADP(+) [cytoplasm] |
| Glycolate transport | glycolate [cytoplasm] --> glycolate [extracellular] |
| Glycolate exhange | glycolate [extracellular] --> |

Toivari, M., Ilmén, M., Penttilä, M., Improved production of oxalyl-CoA, glyoxylate and/or glycolic acid. US20200199632A1, 2020

**Supplementary Table 7**. Strains used and constructed in this work.

| **Name** | **Description** |
| --- | --- |
| H3887 | Wild type *S. cerevsiae* CEN.PK113-7D *(MATa URA3 HIS3 LEU2 TRP1 MAL2-8c SUC2*) * |
| H5746 | H3887 with *FAT2* *panE2* *OXA* |
| H5770 | H5746 with *GLYR1* |
| H5677 | H5746 with *icl1Δ1 ser3Δ1 ser33Δ1* |
| H5763 | H5746 with *icl1Δ1 ser3Δ1 ser33Δ1* |
| H5971 | Isolate H5677_1 |
| H5972 | Isolate H5677_2 |
| H5973 | Isolate H5677_3 |
| H5974 | Isolate H5763_1 |
| H5975 | Isolate H5763_2 |
| H5976 | Isolate H5763_3 |
| H5977 | Isolate H5677_1 with *GLYR1* |
| H5978 | Isolate H5677_2 with *GLYR1* |
| H5979 | Isolate H5677_3 with *GLYR1* |
| H5980 | Isolate H5763_1 with *GLYR1* |
| H5981 | Isolate H5763_2 with *GLYR1* |
| H5982 | Isolate H5763_3 with *GLYR1* |
| H6741 | H5977 with *agx1∆1* |
| H6742 | H5978 with *agx1∆1* |
| H6743 | H5979 with *agx1∆1* |
| H6744 | H5980 with *agx1∆1* |
| H6745 | H5981 with *agx1∆1* |
| H6746 | H5982 with *agx1∆1* |

*The parental CEN.PK yeast strains were obtained from Dr. P. Kötter (J.W. Goethe Universität, Germany).

**Supplementary Table 8**. Modifications to flux lower and upper bounds. These lower bounds were set to 0 in *S. cerevisiae* GEM.

| reaction id | reaction name | reaction | bound |
| --- | --- | --- | --- |
| r_0399 | fatty-acid--CoA ligase (decanoate) | ATP [peroxisome] + coenzyme A [peroxisome] + decanoate [peroxisome] <=> AMP [peroxisome] + decanoyl-CoA [peroxisome] + diphosphate [peroxisome] | lb |
| r_0400 | fatty-acid--CoA ligase (dodecanoate) | ATP [peroxisome] + coenzyme A [peroxisome] + laurate [peroxisome] <=>  AMP [peroxisome] + diphosphate [peroxisome] + lauroyl-CoA [peroxisome] | lb |
| r_0402 | fatty-acid--CoA ligase (hexadecanoate) | ATP [peroxisome] + coenzyme A [peroxisome] + palmitate [peroxisome] <=>  AMP [peroxisome] + diphosphate [peroxisome] + palmitoyl-CoA [peroxisome] | lb |
| r_0410 | fatty-acid--CoA ligase (octanoate) | ATP [peroxisome] + coenzyme A [peroxisome] + octanoate [peroxisome] <=>  AMP [peroxisome] + diphosphate [peroxisome] + octanoyl-CoA [peroxisome] | lb |
| r_0412 | fatty-acid--CoA ligase (tetradecanoate) | ATP [peroxisome] + coenzyme A [peroxisome] + myristate [peroxisome] <=>  AMP [peroxisome] + diphosphate [peroxisome] + myristoyl-CoA [peroxisome] | lb |
| r_0446 | formate-tetrahydrofolate ligase | ATP [cytoplasm] + formate [cytoplasm] + THF [cytoplasm] <=>  10-formyl-THF [cytoplasm] + ADP [cytoplasm] + phosphate [cytoplasm] | lb |
| r_0487 | glycerol dehydrogenase (NADP-dependent) | glycerol [cytoplasm] + NADP(+) [cytoplasm] -->  glycerone [cytoplasm] + H+ [cytoplasm] + NADPH [cytoplasm] | ub |
| r_2194 | fatty-acid--CoA ligase (dodecanoate), ER membrane | coenzyme A [endoplasmic reticulum membrane] + ATP [endoplasmic reticulum membrane] +  laurate [endoplasmic reticulum membrane] <=>  lauroyl-CoA [endoplasmic reticulum membrane] + AMP [endoplasmic reticulum membrane] +  diphosphate [endoplasmic reticulum membrane] | lb |
| r_2195 | fatty-acid--CoA ligase (tetradecanoate), ER membrane | coenzyme A [endoplasmic reticulum membrane] + ATP [endoplasmic reticulum membrane] +  myristate [endoplasmic reticulum membrane] <=>  myristoyl-CoA [endoplasmic reticulum membrane] + AMP [endoplasmic reticulum membrane] +  diphosphate [endoplasmic reticulum membrane] | lb |
| r_2196 | fatty-acid--CoA ligase (hexadecanoate), ER membrane | coenzyme A [endoplasmic reticulum membrane] + ATP [endoplasmic reticulum membrane] +  palmitate [endoplasmic reticulum membrane] <=>  palmitoyl-CoA [endoplasmic reticulum membrane] + AMP [endoplasmic reticulum membrane] +  diphosphate [endoplasmic reticulum membrane] | lb |
| r_2197 | fatty-acid--CoA ligase (hexadecenoate), ER membrane | coenzyme A [endoplasmic reticulum membrane] + ATP [endoplasmic reticulum membrane] +  palmitoleate [endoplasmic reticulum membrane] <=>  palmitoleoyl-CoA(4-) [endoplasmic reticulum membrane] + AMP [endoplasmic reticulum membrane] +  diphosphate [endoplasmic reticulum membrane] | lb |
| r_2198 | fatty-acid--CoA ligase (octadecanoate), ER membrane | coenzyme A [endoplasmic reticulum membrane] + ATP [endoplasmic reticulum membrane] +  stearate [endoplasmic reticulum membrane] <=>  stearoyl-CoA [endoplasmic reticulum membrane] + AMP [endoplasmic reticulum membrane] +  diphosphate [endoplasmic reticulum membrane] | lb |
| r_2199 | fatty-acid--CoA ligase (octadecenoate), ER membrane | coenzyme A [endoplasmic reticulum membrane] + ATP [endoplasmic reticulum membrane] +  oleate [endoplasmic reticulum membrane] <=>  oleoyl-CoA [endoplasmic reticulum membrane] + AMP [endoplasmic reticulum membrane] +  diphosphate [endoplasmic reticulum membrane] | lb |
| r_2200 | fatty-acid-CoA ligase (dodecanoate), lipid particle | coenzyme A [lipid particle] + ATP [lipid particle] + laurate [lipid particle] <=>  diphosphate [lipid particle] + AMP [lipid particle] + lauroyl-CoA [lipid particle] | lb |
| r_2201 | fatty-acid--CoA ligase (tetradecanoate), lipid particle | coenzyme A [lipid particle] + ATP [lipid particle] + myristate [lipid particle] <=>  diphosphate [lipid particle] + AMP [lipid particle] + myristoyl-CoA [lipid particle] | lb |
| r_2202 | fatty-acid--CoA ligase (hexadecanoate), lipid particle | coenzyme A [lipid particle] + ATP [lipid particle] + palmitate [lipid particle] <=>  diphosphate [lipid particle] + AMP [lipid particle] + palmitoyl-CoA [lipid particle] | lb |
| r_2203 | fatty-acid--CoA ligase (hexadecenoate), lipid particle | coenzyme A [lipid particle] + ATP [lipid particle] + palmitoleate [lipid particle] <=>  diphosphate [lipid particle] + AMP [lipid particle] + palmitoleoyl-CoA(4-) [lipid particle] | lb |
| r_2204 | fatty-acid--CoA ligase (octadecanoate), lipid particle | coenzyme A [lipid particle] + ATP [lipid particle] + stearate [lipid particle] <=>  diphosphate [lipid particle] + AMP [lipid particle] + stearoyl-CoA [lipid particle] | lb |
| r_2205 | fatty-acid--CoA ligase (octadecenoate), lipid particle | coenzyme A [lipid particle] + ATP [lipid particle] + oleate [lipid particle] <=>  diphosphate [lipid particle] + AMP [lipid particle] + oleoyl-CoA [lipid particle] | lb |
| r_2206 | fatty-acid-CoA ligase (hexadecenoate), peroxisome | ATP [peroxisome] + coenzyme A [peroxisome] + palmitoleate [peroxisome] <=>  AMP [peroxisome] + diphosphate [peroxisome] + palmitoleoyl-CoA(4-) [peroxisome] | lb |
| r_2207 | fatty-acid--CoA ligase (octadecanoate), peroxisome | ATP [peroxisome] + coenzyme A [peroxisome] + stearate [peroxisome] <=>  AMP [peroxisome] + diphosphate [peroxisome] + stearoyl-CoA [peroxisome] | lb |
| r_2208 | fatty-acid--CoA ligase (octadecenoate), peroxisome | ATP [peroxisome] + coenzyme A [peroxisome] + oleate [peroxisome] <=>  AMP [peroxisome] + diphosphate [peroxisome] + oleoyl-CoA [peroxisome] | lb |
| r_2209 | fatty-acid--CoA ligase (arachidate), cell envelope | ATP [cell envelope] + coenzyme A [cell envelope] + arachidate [cell envelope] <=>  AMP [cell envelope] + diphosphate [cell envelope] + icosanoyl-CoA [cell envelope] | lb |
| r_2210 | fatty-acid--CoA ligase (behenate), cell envelope | ATP [cell envelope] + coenzyme A [cell envelope] + behenate [cell envelope] <=>  AMP [cell envelope] + diphosphate [cell envelope] + docosanoyl-CoA [cell envelope] | lb |
| r_2211 | fatty-acid--CoA ligase (lignoceric acid), cell envelope | ATP [cell envelope] + coenzyme A [cell envelope] + lignoceric acid [cell envelope] <=>  AMP [cell envelope] + diphosphate [cell envelope] + tetracosanoyl-CoA [cell envelope] | lb |
| r_2212 | fatty-acid--CoA ligase (cerotic acid), cell envelope | ATP [cell envelope] + coenzyme A [cell envelope] + cerotic acid [cell envelope] <=>  AMP [cell envelope] + diphosphate [cell envelope] + hexacosanoyl-CoA [cell envelope] | lb |
| r_2213 | fatty-acid--CoA ligase (behenate), ER membrane | coenzyme A [endoplasmic reticulum membrane] + ATP [endoplasmic reticulum membrane] +  behenate [endoplasmic reticulum membrane] <=>  docosanoyl-CoA [endoplasmic reticulum membrane] +  AMP [endoplasmic reticulum membrane] + diphosphate [endoplasmic reticulum membrane] | lb |
| r_2214 | fatty-acid--CoA ligase (lignoceric acid), ER membrane | coenzyme A [endoplasmic reticulum membrane] + ATP [endoplasmic reticulum membrane] +  lignoceric acid [endoplasmic reticulum membrane] <=>  tetracosanoyl-CoA [endoplasmic reticulum membrane] +  AMP [endoplasmic reticulum membrane] + diphosphate [endoplasmic reticulum membrane] | lb |
| r_2215 | fatty-acid--CoA ligase (cerotic acid), ER membrane | Coenzyme A [endoplasmic reticulum membrane] + ATP [endoplasmic reticulum membrane] +  cerotic acid [endoplasmic reticulum membrane] <=>  hexacosanoyl-CoA [endoplasmic reticulum membrane] +  AMP [endoplasmic reticulum membrane] + diphosphate [endoplasmic reticulum membrane] | lb |
| r_2216 | fatty-acid-CoA ligase (behenate), lipid particle | coenzyme A [lipid particle] + ATP [lipid particle] + behenate [lipid particle] <=>  diphosphate [lipid particle] + AMP [lipid particle] + docosanoyl-CoA [lipid particle] | lb |
| r_2217 | fatty-acid--CoA ligase (lignoceric acid), lipid particle | coenzyme A [lipid particle] + ATP [lipid particle] + lignoceric acid [lipid particle] <=>  diphosphate [lipid particle] + AMP [lipid particle] + tetracosanoyl-CoA [lipid particle] | lb |
| r_2218 | fatty-acid--CoA ligase (cerotic acid), lipid particle | coenzyme A [lipid particle] + ATP [lipid particle] + cerotic acid [lipid particle] <=>  diphosphate [lipid particle] + AMP [lipid particle] + hexacosanoyl-CoA [lipid particle] | lb |
| r_4158 | NADPH2:quinone oxidoreductase | H+ [cytoplasm] + NADPH [cytoplasm] + 2.0 quinone [cytoplasm] <=>  NADP(+) [cytoplasm] + 2.0 1,4-benzosemiquinone [cytoplasm] | lb |
| r_4198 | Benzil reductase ((S)-benzoin forming) IRC24 (EC 1.1.1.320) (Increased recombination centers protein 24) | NADP(+) [cytoplasm] + (S)-benzoin [cytoplasm] <=>  H+ [cytoplasm] + NADPH [cytoplasm] + benzil [cytoplasm] | ub |
| r_4275 | Fe(II):NADP+ oxidoreductase | NADPH [cytoplasm] + 2.0 iron(3+) [extracellular] <=>  H+ [cytoplasm] + 2.0 iron(2+) [cytoplasm] + NADP(+) [cytoplasm] | lb |
| r_4276 | Fe(II):NADP+ oxidoreductase | NADPH [cytoplasm] + 2.0 iron(3+) [vacuole] <=>  H+ [cytoplasm] + 2.0 iron(2+) [cytoplasm] + NADP(+) [cytoplasm] | lb |
| r_4292 | 4-Aminobutyraldehyde:NAD+ oxidoreductase | 4-aminobutanal [cytoplasm] + H2O [cytoplasm] + NADP(+) [cytoplasm] <=>  gamma-aminobutyrate [cytoplasm] + 2.0 H+ [cytoplasm] + NADPH [cytoplasm] | lb |
| r_4330 | Pyridoxine:NADP+ 4-oxidoreductase | NADP(+) [cytoplasm] + pyridoxine [cytoplasm] <=>  H+ [cytoplasm] + NADPH [cytoplasm] + pyridoxal [cytoplasm] | ub |
| r_4490 | Xylitol:NADP+ 4-oxidoreductase (L-xylulose-forming) | H+ [cytoplasm] + NADPH [cytoplasm] + L-xylulose [cytoplasm] <=>  NADP(+) [cytoplasm] + xylitol [cytoplasm] | lb |
| r_4576 | (R)-2,3-Dihydroxy-3-methylbutanoate: NADP+ oxidoreductase (isomerizing) | NADP(+) [cytoplasm] + (R)-2,3-dihydroxy-3-methylbutanoate [cytoplasm] <=>  H+ [cytoplasm] + NADPH [cytoplasm] + 3-hydroxy-3-methyl-2-oxobutanoate [cytoplasm] | ub |
| r_4580 | (R)-2,3-dihydroxy-3-methylbutanoate: NADP+ oxidoreductase (isomerizing) | NADP(+) [cytoplasm] + (R)-2,3-dihydroxy-3-methylbutanoate [cytoplasm] <=>  H+ [cytoplasm] + NADPH [cytoplasm] + (2S)-2-acetolactate [cytoplasm] | ub |

**Supplementary Table 9**. Plasmids used and constructed in this work

| **Name** | **Description** | **Reference** |
| --- | --- | --- |
| B7770 | pJKLiF-045 Cas9 (KanMX) | Kuivanen et al. 2018 |
|  | **Plasmid constructs** |  |
| B11962 | X-2-PGK1p-FAT2 | Kakko et al. 2023 |
| B11964 | XI-5-TEF1p-OXA-PGK1t | Kakko et al. 2023 |
| B11963 | XII-4-TDH3p-panE2 | Kakko et al. 2023 |
| B11795 | XII-5-TPI1p-GLYR1 | Kakko et al. 2023 |
| B11968 | pCR-ICL1 deletion cassette | This article |
| B11969 | pCR-SER33 deletion cassette | This article |
| B11970 | pCR-SER3 deletion cassette | This article |
|  | **gRNA plasmids** |  |
| B11965 | XI-2-ICL1 gRNA | This article |
| B11966 | XI-2-SER33 gRNA | This article |
| B11967 | XI-2-SER3 gRNA | This article |
| B11954 | XI-2-AGX1 gRNA | This article |
| B10453 | X-2, XI-5, and XII-4 gRNA pCfB3053 | Jessop‐Fabre et al. 2016 |

Kuivanen, J.; Holmström, S.; Lehtinen, B.; Penttilä, M.; Jäntti, J., A High-Throughput Workflow for CRISPR/Cas9 Mediated Combinatorial Promoter Replacements and Phenotype Characterization in Yeast. Biotechnology Journal 2018, 13 (9), 1700593.

Kakko, N., Rantasalo, A., Koponen, T., Vidgren, V., Kannisto, M., Maiorova, N., Nygren, H., Mojzita, D., Penttilä, M. and Jouhten, P., 2023. Inducible Synthetic Growth Regulation Using the ClpXP Proteasome Enhances cis, cis-Muconic Acid and Glycolic Acid Yields in Saccharomyces cerevisiae. ACS Synthetic Biology, 12 (4), 1021-1033.

Jessop‐Fabre, M. M.; Jakočiūnas, T.; Stovicek, V.; Dai, Z.; Jensen, M. K.; Keasling, J. D.; Borodina, I., EasyClone‐MarkerFree: A vector toolkit for marker‐less integration of genes into Saccharomyces cerevisiae via CRISPR‐Cas9. Biotechnology Journal 2016, 11 (8), 1110-1117.

**Supplementary Table 10**. Heterologous genes used.

| **Name** | **Description** | **Reference** |
| --- | --- | --- |
| *FAT2* | *S. cerevisiae* strain S288C oxalyl-CoA synthetase  GenBank: 852523  ORF *S. cerevisiae* (PCS60 YBR222C) | Toivari et al. 2020 |
| *OXA* | *Aspergillus niger* oxaloacetase | Toivari et al. 2020 |
| *panE2* | *Methylobacterium extorquens* oxalyl-CoA reductase  ORF *M. extorquens* (WP_01 5822665). | Toivari et al. 2020 |
| *GLYR1* | *Arabidopsis thaliana* glyoxylate reductase  GenBank: 822139 | Toivari et al. 2020 |

Toivari, M., Ilmén, M., Penttilä, M., Improved production of oxalyl-CoA, glyoxylate and/or glycolic acid. US20200199632A1, 2020

**Supplementary Table 11.** ENA codes for study accession number PRJEB49512.

| **Acession number** | **Lineage** | **Sample** |
| --- | --- | --- |
| ERS9600773 | H5677_1 | end-point population |
| ERS9600774 | H5677_1 | intermediate population |
| ERS9600775 | H5677_2 | intermediate population |
| ERS9600776 | H5677_2 | end-point population |
| ERS9600777 | H5677_1 | evolved isolate |
| ERS9600778 | H5677_2 | evolved isolate |
| ERS9600779 | H5677_3 | evolved isolate |
| ERS9600780 | H5677_3 | intermediate population |
| ERS9600781 | H5677_3 | end-point population |
| ERS9600782 | H5677 | parental clone |
| ERS9600783 | H5763_1 | intermediate population |
| ERS9600784 | H5763_1 | end-point population |
| ERS9600785 | H5763_2 | intermediate population |
| ERS9600786 | H5763_2 | end-point population |
| ERS9600787 | H5763_1 | evolved isolate |
| ERS9600788 | H5763_2 | evolved isolate |
| ERS9600789 | H5763_3 | evolved isolate |
| ERS9600790 | H5763_3 | intermediate population |
| ERS9600791 | H5763_3 | end-point population |
| ERS9600792 | H5763 | parental clone |
| ERS9608795 | H5677_1 | intermediate population |
| ERS9608796 | H5677_2 | intermediate population |
| ERS9608797 | H5677_2 | end-point population |
| ERS9608798 | H5763_1 | intermediate population |
| ERS9608799 | H5763_3 | intermediate population |
| ERS9608800 | H5763_3 | end-point population |

**Supplementary Table 12.** Integration cassettes used as reference sequences for SNV analysis of heterologous genes.

| **FAT2 integration cassette** |
| --- |
| CGTCTATGAGGAGACTGTTAGTTGGATATCAGTAATGAGACGAAAAAGCTCGAAATGAATGGTATATTCTTTTTGCTACTGGCAACTGTTGAATATTTAATGTTAAAACAAACTAACTGAGGTATATTCGTATCTGTATGTACACATATACTATATACAGGAAAAGATAAGCAAGAGAGAGGATATCAACTACGAGAGCGATCGATTATATATCAAAAGCTGTCCGCTTTGCCACCCATAATCGGCGCTTAGTTTCGGAGTTCAATCATAATTCTACCACCTTACACTCAACTTACTCTTTAACTCCTATAGTATAATATCGCCACTGACCCCATATTAAAAAATTTTTTTGCTCGATCTTCTATCCTCTTTAGGTTAATTGTCGCTGTTATTGTCTAGATTTTTTCTCGGAGATGGCGCATCTATTTGCCGTCAAAAGATCCTCTCATACCATATTAAGTAAATTGCCTCCATTTCTTTTTCCTCGGGCAGAGAAACTCGCAGGCAACTTGCTCTCGAAGTGGTCACGTATTAAGTCCTCAGCGAGCTCGCATGGAATGCGTGCGATTTATTTAGAAGTGTCAACAACGTATCTACCAACGGAATGCGTGCGGTGAGTAAGGAAAGAGTGAGGAACTATCGCATACCTGCATTTAAAGATGCCGATTTGGGCGCGAATCCTTTATTTTGGCTTCACCCTCATACTATTATCAGGGCCAGAAAAAGGAAGTGTTTCCCTCCTTCTTGAATTGATGTTACCCTCATAAAGCACGTGGCCTCTTATCGAGAAAGAAATTACCGTCGCTCGTGATTTGTTTGCAAAAAGAACAAAACTGAAAAAACCCAGACACGCTCGACTTCCTGTCATCCTATTGATTGCAGCTTCCAATTTCGTCACACAACAAGGTCCTAGCGACGGCTCACAGGTTTTGTAACAAGCAATCGAAGGTTCTGGAATGGCGGGAAAGGGTTTAGTACCACATGCTATGATGCCCACTGTGATCTCCAGAGCAAAGTTCGTTCGATCGTACTGTTACTCTCTCTCTTTCAAACAGAATTGTCCGAATCGTGTGACAACAACAGCCTGTTCTCACACACTCTTTTCTTCTAACCAAGGGGGTGGTTTAGTTTAGTAGAACCTCGTGAAACTTACATTTACATATATATAAACTTGCATAAATTGGTCAATGCAAGAAATACATATTTGGTCTTTTCTAATTCGTAGTTTTTCAAGTTCTTAGATGCTTTCTTTTTCTCTTTTTTACAGATCATCAAGGAAGTAATTATCTACTTTTTACAACAAATATAAAACAATGACAAGTGCCGCTACTGTTACTGCTTCGTTCAACGATACTTTTAGCGTATCCGATAATGTCGCCGTTATTGTTCCTGAAACGGACACTCAGGTGACCTACAGGGATCTATCCCACATGGTGGGTCACTTCCAGACCATGTTCACAAATCCTAATTCTCCATTGTACGGAGCTGTTTTCAGACAAGATACAGTGGCGATATCCATGCGTAATGGGCTGGAATTTATCGTCGCTTTCCTCGGTGCTACTATGGACGCTAAAATTGGCGCGCCCTTGAATCCCAATTATAAGGAAAAGGAGTTCAATTTTTATTTGAATGACCTGAAATCTAAGGCGATTTGCGTCCCAAAGGGTACCACAAAGTTACAGAGTTCTGAAATTCTAAAATCTGCCTCCACGTTTGGATGTTTTATCGTAGAGCTGGCCTTCGATGCGACCAGGTTTAGGGTAGAGTATGATATATACTCTCCAGAGGACAACTACAAAAGGGTTATTTACCGGTCTTTGAACAACGCCAAATTTGTCAACACAAATCCCGTTAAATTCCCTGGGTTTGCCCGTTCCAGTGACGTTGCCCTGATTTTGCATACCAGTGGTACCACCTCCACTCCAAAAACGGTGCCTTTGTTACATTTGAACATTGTGAGAAGCACGTTGAACATTGCTAACACTTACAAGCTAACGCCCTTGGACAGATCTTATGTCGTGATGCCTCTTTTCCACGTCCATGGGTTAATTGGTGTTTTACTTTCCACTTTTAGAACTCAGGGTTCTGTTGTGGTTCCCGATGGATTCCATCCAAAGTTATTCTGGGACCAATTTGTTAAGTACAACTGTAATTGGTTCAGTTGCGTTCCCACAATAAGCATGATTATGCTGAACATGCCCAAACCAAACCCTTTCCCACACATTAGATTCATCAGATCGTGTTCTTCTGCTTTGGCTCCAGCAACGTTCCATAAGCTGGAGAAGGAATTCAATGCACCTGTCTTGGAGGCCTATGCGATGACCGAAGCATCACATCAAATGACCTCAAACAATCTGCCTCCAGGAAAGAGAAAGCCTGGTACTGTGGGCCAGCCACAAGGAGTCACCGTCGTCATTCTAGATGACAATGACAATGTCTTGCCCCCGGGCAAAGTCGGCGAAGTTTCCATCAGAGGCGAAAACGTCACTTTGGGGTATGCTAATAATCCAAAAGCTAACAAGGAGAACTTCACCAAGAGAGAGAACTATTTCAGAACCGGTGACCAAGGTTATTTCGACCCTGAGGGGTTTTTGGTCCTTACAGGCAGAATCAAAGAGCTTATCAACAGGGGTGGTGAAAAGATTTCACCCATTGAGCTCGACGGCATTATGCTATCGCATCCAAAGATCGATGAAGCCGTTGCATTTGGTGTTCCCGACGATATGTACGGCCAAGTAGTTCAAGCCGCCATTGTTTTGAAGAAGGGAGAAAAAATGACCTACGAAGAACTGGTGAACTTCTTAAAGAAGCACCTAGCCTCTTTCAAAATTCCAACCAAGGTGTACTTTGTTGATAAGCTACCAAAAACCGCTACAGGTAAAATCCAGAGAAGAGTTATCGCAGAAACTTTTGCTAAGAGCAGCAGAAATAAGTAGCGCGTGCATTCATCCGCTCTAACCGAAAAGGAAGGAGTTAGACAACCTGAAGTCTAGGTCCCTATTTATTTTTTTATAGTTATGTTAGTATTAAGAACGTTATTTATATTTCAAATTTTTCTTTTTTTTCTGTACAGACGCGTGTACGCATGTAACATTATACTGAAAACCTTGCTTGAGAAGGTTTTGGGACGCTCGAAGATCGCGTCCCAATTCGCCCTATAGTGAGTCGTATTACGCGCGCTCACTGGCCGTCGTTTTACAACGTCGTGACTGGGAAAACCCTGGCGTTACCCCTGCAGGACTAGTGCTGAGGCATTAATCCTGCATAATCGGCCTCACAGAGGGATCCCGTTACCCATCTATGCTGAAGATTTATCATACTATTCCTCCGCTCGTTTCTTTTTTCAGTGAGGTGTGTCGTGAAAGAAAACCCACAATTAAACTTTCAACAACCGGGCGACTAGGAAGAGAGTAGTGGGCGCGGATGACGAAGGCTAAGGTCACTTCTTCGTTTCCTTTATTGGGGTTTCCGTGTAGCCTTCCCCTGAATAGTGTGGGACGTTTTATGAGAAGCCGTAAGAAATAGGCAAATTGAGTTATGACAAGTAGACATGATGCCGCAGCCTTGCCTGACTTTACGTCTCCTTCATGAATAAGTTTTTCTATCGAGTTCTTTTCCTTTTTTCGCCTTAATTAGCTCAATTAAGCCTGTCCTCACTACTTTTCTTTTTCTTATCGGCTTTGTGCCACACCTAACCTTCGAATGCTGTTTTATTCCGTTCTTACATGGGATGGTAATGCCTTGGCGAGA |
| **OXA integration cassette** |
| GCGGAGAAGTCGTTGATAGCATTTCCGAAGGCTTTTCCACATTGACTGCAGTATCTAAAAGAGCTCCAGCAGCGGTCTTAGTAGTTCCTGTGCTTGCCGCTTTGCAGTATACAGGGACATTGGGAACTACGGAGGAAAAATCGGAATGCTCTGAGCTCTTCTCAAAACCAGACGAACTCGTCTTATTTCTCAGTAAATTACCCAACATATAAACTCTCCCTCTTCACCTTCTCTACAAGGCCACACACAAGACAGTTTTAACAGGCAAATGGTACTGGAGGCTCTCACCAATGGGGTTTAAGACGCTCCAAACACACGCTCTCCTTTTTTTCTTTATATATCGGTAGCAACGAAAGCTAGTCGCAGAACGTTACACACTAAAGCAGATCAATCAATCAATCCTGAGAACCTGCTTCGTTCAAACCATCCACTAGAAGAAACACGCACGTGTACCCAAAAATAGAGACAAACAAACAGACAAAAATAACAATGATTCCTACCGACTAAAGTAAAACAACACTCACGTAATAAAAAATCCTAGATACAACTCACTTGTATTCCTCTCTTCTTTGACCATCACTAAAGCTTCTCTCTTACTATATTCCTCAAATAAAAAAAAGTTGCAATGATTACTTACCAATGTGCCATAAACTCCGTGCACCAATTAAGTCCTCAGCGAGCTCGCATGGAATGCGTGCGATTTATTTAGAAGTGTCAACAACGTATCTACCAACGGAATGCGTGCGCCTTGCCAACAGGGAGTTCTTCAGAGACATGGAGGCTCAAAACGAAATTATTGACAGCCTAGACATCAATAGTCATACAACAGAAAGCGACCACCCAACTTTGGCTGATAATAGCGTATAAACAATGCATACTTTGTACGTTCAAAATACAATGCAGTAGATATATTTATGCATATTACATATAATACATATCACATAGGAAGCAACAGGCGCGTTGGACTTTTAATTTTCGAGGACCGCGAATCCTTACATCACACCCAATCCCCCACAAGTGATCCCCCACACACCATAGCTTCAAAATGTTTCTACTCCTTTTTTACTCTTCCAGATTTTCTCGGACTCCGCGCATCGCCGTACCACTTCAAAACACCCAAGCACAGCATACTAAATTTCCCCTCTTTCTTCCTCTAGGGTGTCGTTAATTACCCGTACTAAAGGTTTGGAAAAGAAAAAAGAGACCGCCTCGTTTCTTTTTCTTCGTCGAAAAAGGCAATAAAAATTTTTATCACGTTTCTTTTTCTTGAAAATTTTTTTTTTTGATTTTTTTCTCTTTCGATGACCTCCCATTGATATTTAAGTTAATAAACGGTCTTCAATTTCTCAAGTTTCAGTTTCATTTTTCTTGTTCTATTACAACTTTTTTTACTTCTTGCTCATTAGAAAGAAAGCATAGCAATCTAATCTAAGTTTTAATTACAAAATGAAGGTTGACACCCCAGACTCCGCTTCTACTATTTCTATGACTAACACCATTACCATCACCGTTGAACAAGATGGTATCTACGAAATCAATGGTGCTAGACAAGAACCAGTTGTCAACTTGAATATGGTTACTGGTGCTTCCAAGTTGAGAAAGCAATTGAGAGAAACTAACGAATTATTGGTTTGCCCAGGTGTTTACGATGGTTTGTCTGCTAGAATTGCTATCAACTTGGGTTTCAAGGGTATGTATATGACTGGTGCTGGTACTACTGCTTCAAGATTGGGTATGGCTGATTTGGGTTTGGCTCATATCTATGACATGAAGACTAACGCTGAAATGATTGCTAATTTGGACCCATACGGTCCACCTTTGATTGCTGATATGGATACAGGTTATGGTGGTCCATTGATGGTTGCTAGATCTGTCCAACAATATATTCAAGCTGGTGTTGCCGGTTTCCATATCGAAGATCAAATTCAAAACAAGAGATGCGGTCATTTGGCTGGTAAAAGAGTTGTTACTATGGACGAATACTTGACCAGAATTAGAGCTGCTAAGTTGACCAAGGATAGATTGAGATCTGACATCGTTTTGATTGCAAGAACTGATGCCTTGCAACAACATGGTTACGATGAATGCATTAGAAGATTGAAGGCTGCTAGAGATTTGGGTGCTGATGTTGGTTTGTTGGAAGGTTTCACTTCTAAAGAAATGGCCAGAAGATGCGTTCAAGATTTGGCTCCATGGCCTTTGTTGTTGAACATGGTTGAAAATGGTGCAGGTCCAGTTATCTCTGTTGATGAAGCTAGAGAAATGGGTTTCAGAATTATGATTTTCTCCTTCGCTTGTATTACCCCAGCTTACATGGGTATTACTGCTGCTTTGGAAAGATTGAAAAAGGATGGTGTTGTAGGTTTGCCAGAAGGTATGGGTCCTAAAAAGTTGTTTGAAGTTTGCGGTTTGATGGACTCCGTTAGAGTTGATACTGAAGCTGGTGGTGATGGTTTTGCTAACGGTGTTTGATTAATTAACCCATGTCTCTACTGGTGGTGGTGCTTCTTTGGAATTATTGGAAGGTAAGGAATTGCCAGGTGTTGCTTTCTTATCCGAAAAGAAATAAATTGAATTGAATTGAAATCGATAGATCAATTTTTTTCTTTTCTCTTTCCCCATCCTTTACGCTAAAATAATAGTTTATTTTATTTTTTGAATATTTTTTATTTATATACGTATATATAGACTATTATTTATCTTTTAATGATTATTAAGATTTTTATTAAAAAAAAATTCGCTCCTCTTTTAATGCCTTTATGCAGTTTTTTTTTCCCATTCGATATTTCTATGTTCGGGTTCAGCGTATTTTAAGTTTAATAACTCGAAAATTCTGCGTTCGTTAACGCGTGCATTCATCGCGTCCCAATTCGCCCTATAGTGAGTCGTATTACGCGCGCTCACTGGCCGTCGTTTTACAACGTCGTGACTGGGAAAACCCTGGCGTTACCCCTGCAGGACTAGTGCTGAGGCATTACTATTGGCTGCTTCATAGTACACCCAATTGCCCACAACCCAAGTTAATCATAAGCTCTTGTTGTCGATGAATTGCTCAAAATGTGGCCATTCTTTCAATTTTGCACCTCTAAAACTGCTCCGTGGGTTGTGACAACAACCTTAAGGGTAATGGACTTTTTTAGAAAGACAAATGAGATGATTTCACCTACTGGTATATCATTTATTTATACACGACCGTTTTGACATTTCAGAGTTGCACGTTGGTATATCCTAATATTCTCACAAGCATCACAGTGAAGAAACGCAAGAAAATGGAAAAAATTCCCAGGTGGTTGTTGTCTTCTCTTATATCGTCGGTGCTGTGCATACTTGGGGCCCTGTGTGTGCCGTTGTTATCGGTTGCCTTCGATAGCAAGCGCAATAGCCAATCTAAGTTGGTCAACTATGGTCTTTCTCTAAGTGCCGGATCTATGATCA |
| **panE2 integration cassette** |
| TGTATCCGGCTGTTCCTTCATAGCCCTTTCAATGAACGTTGCAGCCCTTTGAAGATTGGCCATTTTGTCAGGACTCGAGCCTGACAGTTGGACCAACGCAACTTTAATTTTTTGTGAAAGAATCTTCGAAGCACTCATACTGGCGATCTTCACGCCCTCCTGCTATTACAAAAGCTGTGTTTTTACAAGAATCAAATTAAGTTAGCAAGATATTATACAACATTATTGATAATTTCAATATCGTGTTCGTACCTGATGACGTATCTGTGCATTGATAAGGCCCGCATGGTTTCAGAAAGCAGAGCGGAACGATTCCAAATTAGTGGCCTTGTGCTTTGCATGTCAATTGTGTTACCTTCAGCTCGTGGATTTGTTTTATCAATACACAGTCTACAGTCAAGAATTTTTTTTATCAAATTTTGCGTTCGAGCGTATAAAATAGCCGCTGTAGCTACTTAAGTTCCTGTTCAGCGATAGTTTTTTTCCATCACACGTACTATGGCAATTAAGTCCTCAGCGAGCTCGCATGGAATGCGTGCGATTTATTTAGAAGTGTCAACAACGTATCTACCAACGGAATGCGTGCGCAGTTCGAGTTTATCATTATCAATACTGCCATTTCAAAGAATACGTAAATAATTAATAGTAGTGATTTTCCTAACTTTATTTAGTCAAAAAATTAGCCTTTTAATTCTGCTGTAACCCGTACATGCCCAAAATAGGGGGCGGGTTACACAGAATATATAACATCGTAGGTGTCTGGGTGAACAGTTTATTCCTGGCATCCACTAAATATAATGGAGCCCGCTTTTTAAGCTGGCATCCAGAAAAAAAAAGAATCCCAGCACCAAAATATTGTTTTCTTCACCAACCATCAGTTCATAGGTCCATTCTCTTAGCGCAACTACAGAGAACAGGGGCACAAACAGGCAAAAAACGGGCACAACCTCAATGGAGTGATGCAACCTGCCTGGAGTAAATGATGACACAAGGCAATTGACCCACGCATGTATCTATCTCATTTTCTTACACCTTCTATTACCTTCTGCTCTCTCTGATTTGGAAAAAGCTGAAAAAAAAGGTTGAAACCAGTTCCCTGAAATTATTCCCCTACTTGACTAATAAGTATATAAAGACGGTAGGTATTGATTGTAATTCTGTAAATCTATTTCTTAAACTTCTTAAATTCTACTTTTATAGTTAGTCTTTTTTTTAGTTTTAAAACACCAAGAACTTAGTTTCGAATAAACACACATAAACAAACAAAATGTCCATTGCTATAGTTGGTGCTGGTGCTATTGGTGGTTATTTGGGTGTTAGATTGGCTGAAGCTGGTGAAGATGTTACTTTCATTGCTAGATCAAACGCTGCTGCTATTCAAGCTGATGGTATGAGATTGATTGAAGAAGATGGTACTGAAATCCACTCCAAGTCTGTTAAGGCTACTAGATCAATGCAAGAAGCTGGTGTTCATGAAGTTGTTTTGTTGACTGTTAAGGCCCATCAAGTTGGTCCAATTGCTGCTGACTTGCATCATTTGATTGGTCCAGATACTGTTGTTGTCACTATGCAAAATGGTATTCCTTGGTGGTATTTCTTGGGTGGTTATTCTGGTGATCATGCTGGTACTAGATTGGAATCTGCTGATCCAGGTGGTTTGATTGCTGATCACTTAGATCCAAAACACGTTATCGGTTCTGTTGTTTATCCAGCTACTGTTTTGACTGATCCAGGTACTGTTAAGGTTATCGAAGGTAATAGATTCGGTTTGGGTGAATTGGATGGTTCCAAATCTGAAAGAGTTTTGGCCTTGTCTCAAAGATTGGCTAGAGCTGGTTTTAGAGCACCAGTTACATCTGATATTAGAGCCGAAATTTGGTTGAAGTTGTGGGGTAATTTGTCCTTCAATCCAATTTCTGCTTTGACCCATGCTACCTTGGAAGATATATGTAGATTTCCAGATACCAGAGCTATTGCTGCTGAAATGATGAGAGAAGCTGAAGTTATTGCTAACAAGTTGGGTGTTACTTTCAGATTGGGTATCGATAAGAGAATTGCTGGTGCTGAAAAAGTTGGTCCACATAAGACTTCTATGTTGCAAGATGTTGAAGCCGGTAGACCAATTGAATTGGAAGCTTTGGTTGGTTCCGTTATCGAATTGGGTAGATTGACTGGTACTCCAACTCCACATATTGATACTGTTTTCGCCTTGATGAGATTATTGGCCCAATCTTTGGAAAGAGCACAAGGTAGATTGGCTATTCAAGGTGCTTAACGCGTGCATTCATCCGCTCTAACCGAAAAGGAAGGAGTTAGACAACCTGAAGTCTAGGTCCCTATTTATTTTTTTATAGTTATGTTAGTATTAAGAACGTTATTTATATTTCAAATTTTTCTTTTTTTTCTGTACAGACGCGTGTACGCATGTAACATTATACTGAAAACCTTGCTTGAGAAGGTTTTGGGACGCTCGAAGATCGCGTCCCAATTCGCCCTATAGTGAGTCGTATTACGCGCGCTCACTGGCCGTCGTTTTACAACGTCGTGACTGGGAAAACCCTGGCGTTACCCCTGCAGGACTAGTGCTGAGGCATTAATAGTTTACTCAATTCTTGAAGCCAATTTGTACAATTCCCCATTAGAGTCAAATAAAAGGATGCCTCACGGAGGTATGTTACCCGCGCTATTTCACATGGCTCATTGAATTAGAGGTGGAATTTGGTGTACCCTCCCCTCCTCATCTGATGAAGTAGTGATCCGACAATTCTTAAAAGTTGTAGACATTACTTTTACCACCAACTAAGTTGTATTTATATTGCTACCCTTATCCTTTTATATCTAACTAGCGCTCATAAGGTTGGGGCAATACTAAAACTGTGTTCTTATTCAACTCATTAAATACGTGGCAGTACGTACCCTATTAGAAACAATAGGAAACAGCAGAGTCGGAAGAAGCCAAATGCCAGATTTGAAGTCCAAAACCTTGTCAAGCCAATCTTTGGGAGCGGCTATTCCTCCAGAAATTGTGTACCAAATACTTACATACCAGTTTAGGGATTTGTTAAGAAATGACCATCCAGGTACGGCAGAAAA |


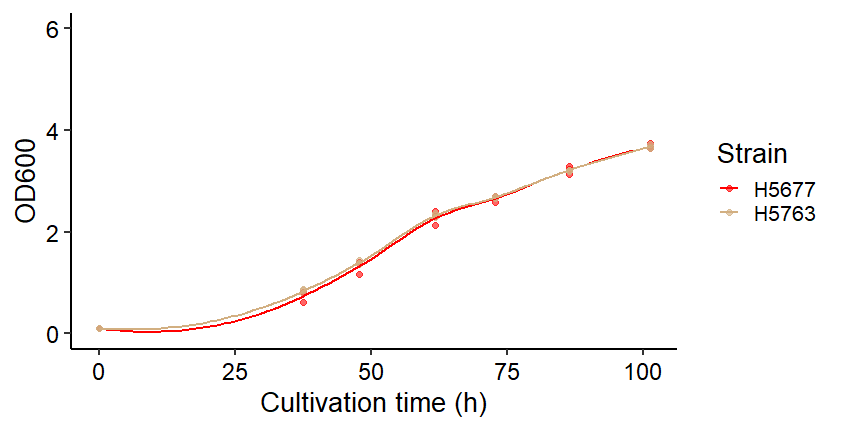


**Supplementary Figure 1.** Growth dynamics as culture turbidity (OD600) as a function of cultivation time of parental strains (H5677 in red and H5763 in beige). Full lines are the *loess* fits to average culture turbidities of three biological replicates.


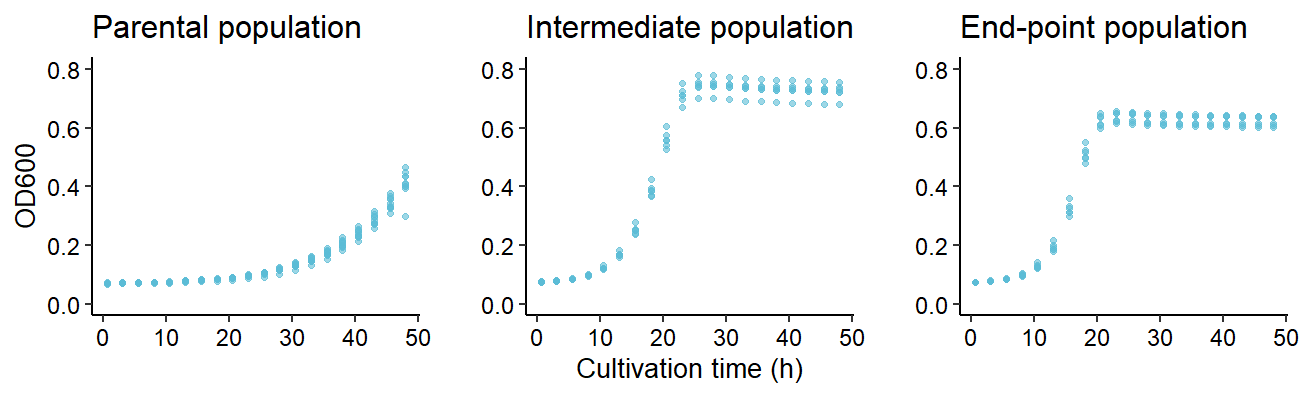


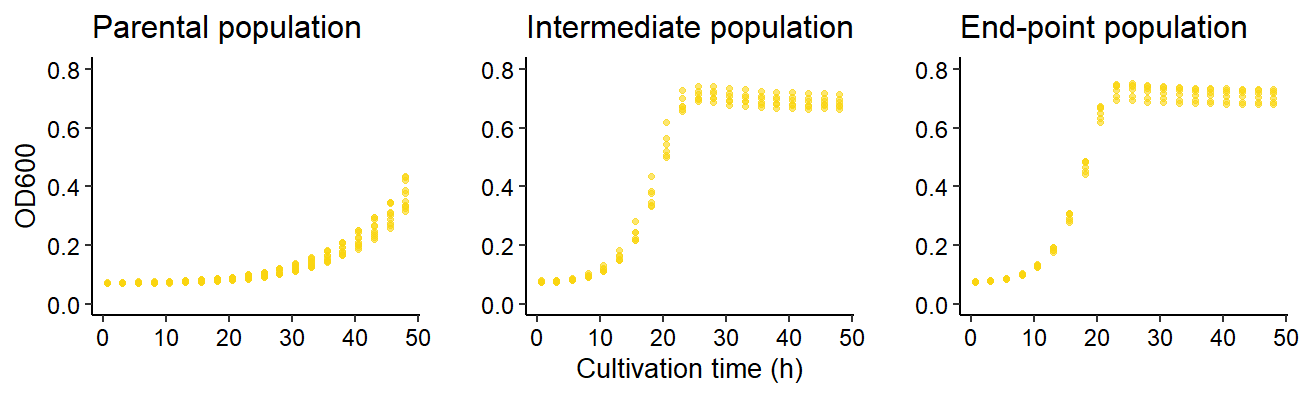


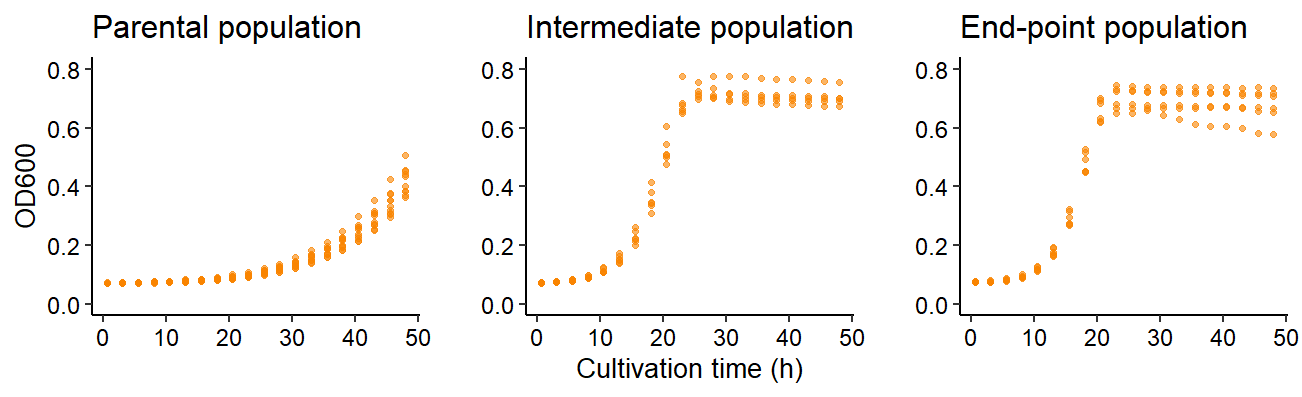


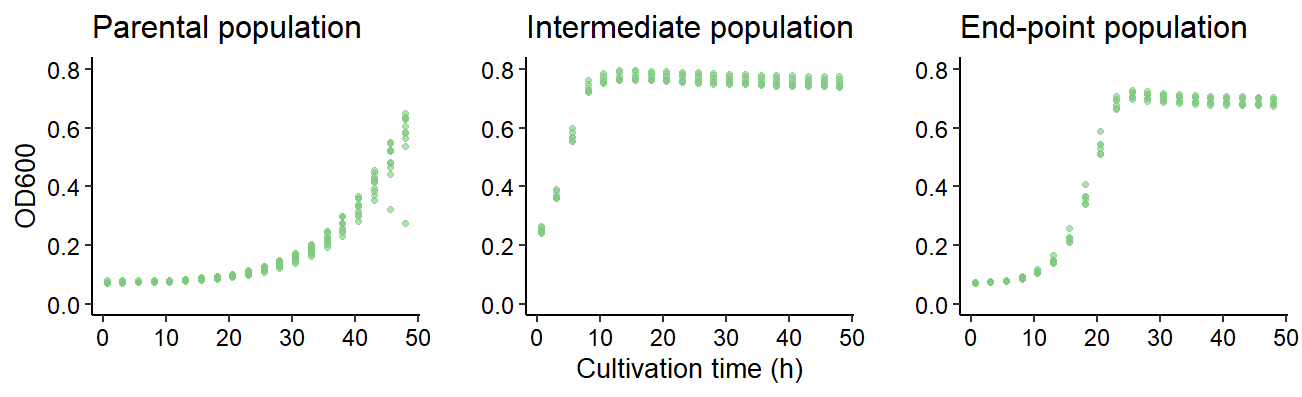


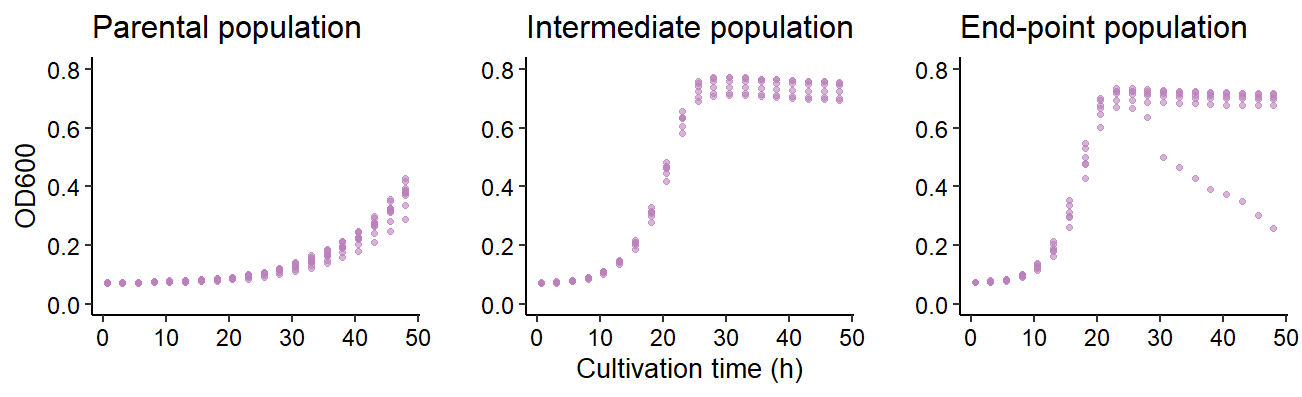


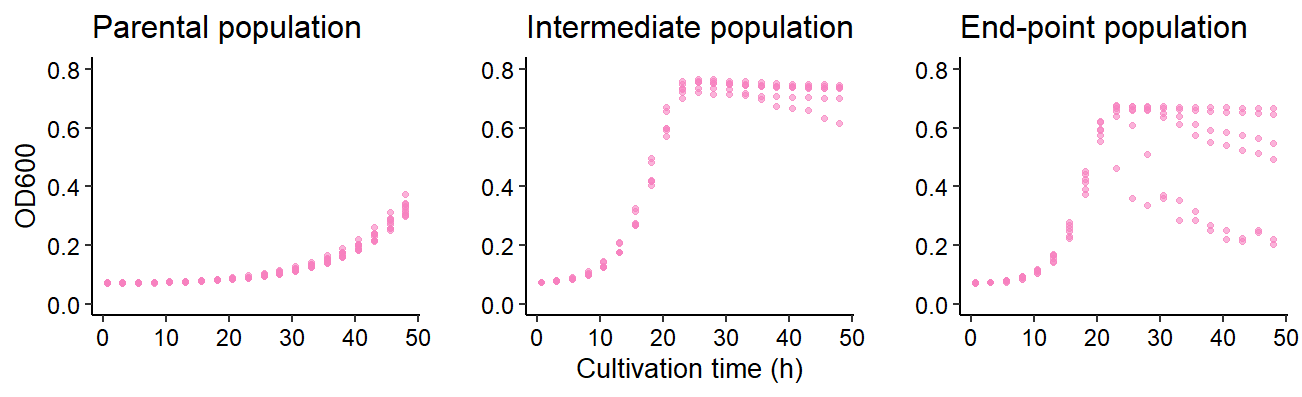


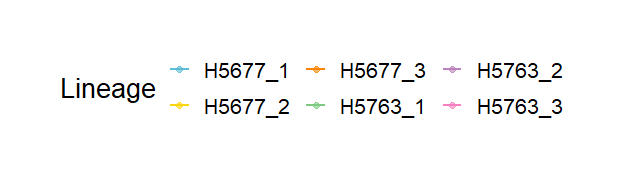


**Supplementary Figure 2.** The bioscreen growth profiles for all six evolution lineages at specific time-points during the ALE experiment. The growth profiles of all 10 biological replicates are shown for each evolved lineage as raw bioscreen values for the parental population (transfer 0), intermediate population (transfer 10) and end-point population (transfer 30). The first 50 h of each cultivation is shown. The growth profiles for lineage H5677_1 are shown in blue, H5677_2 in yellow, H5677_3 in orange, H5763_1 in green, H5763_2 in purple and H5763_3 in pink.


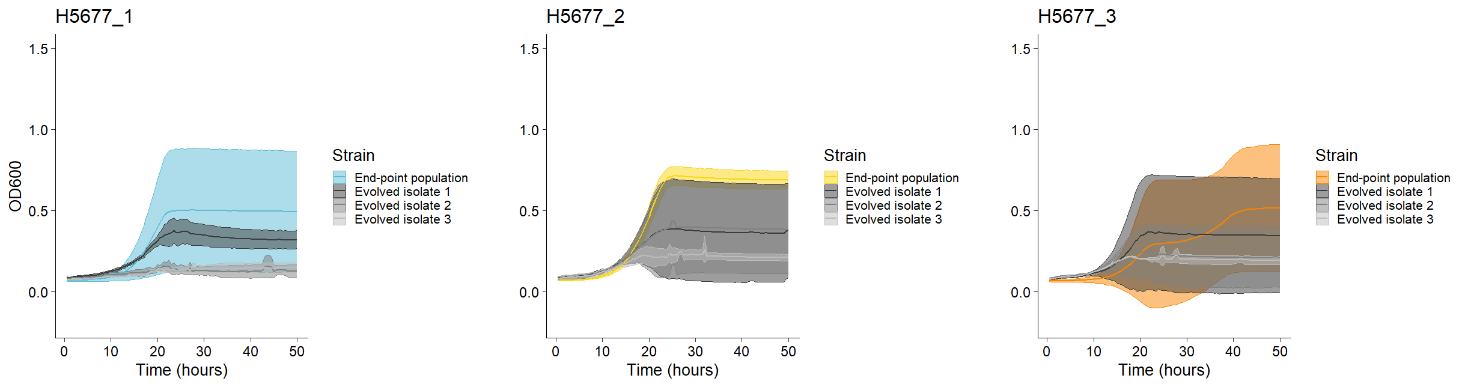


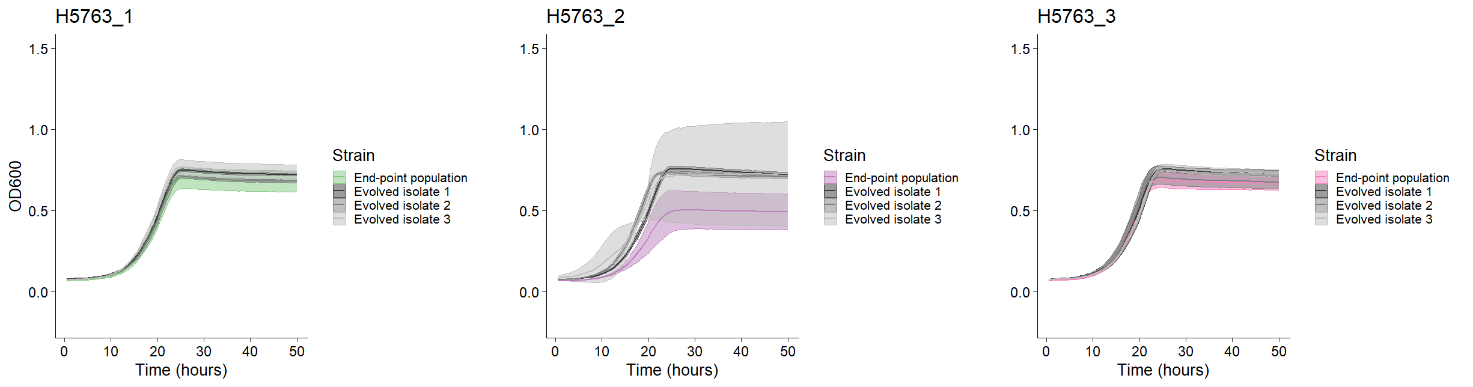


**Supplementary Figure 3:** Bioscreen growth profiles of end-point populations compared to all randomly chosen evolved isolates, for all six evolution lineages, respectively. Full lines are the average of raw bioscreen values of 3 biological replicates. The ribbon surrounding each average line shows the mean ± standard deviation. The first 50 h of each cultivation is shown. The growth profile for the end-point population of lineage H5677_1 is shown in blue, H5677_2 in yellow, H5677_3 in orange, H5763_1 in green, H5763_2 in purple and H5763_3 in pink, with the evolved isolates in shades of grey shown with their respective lineage.


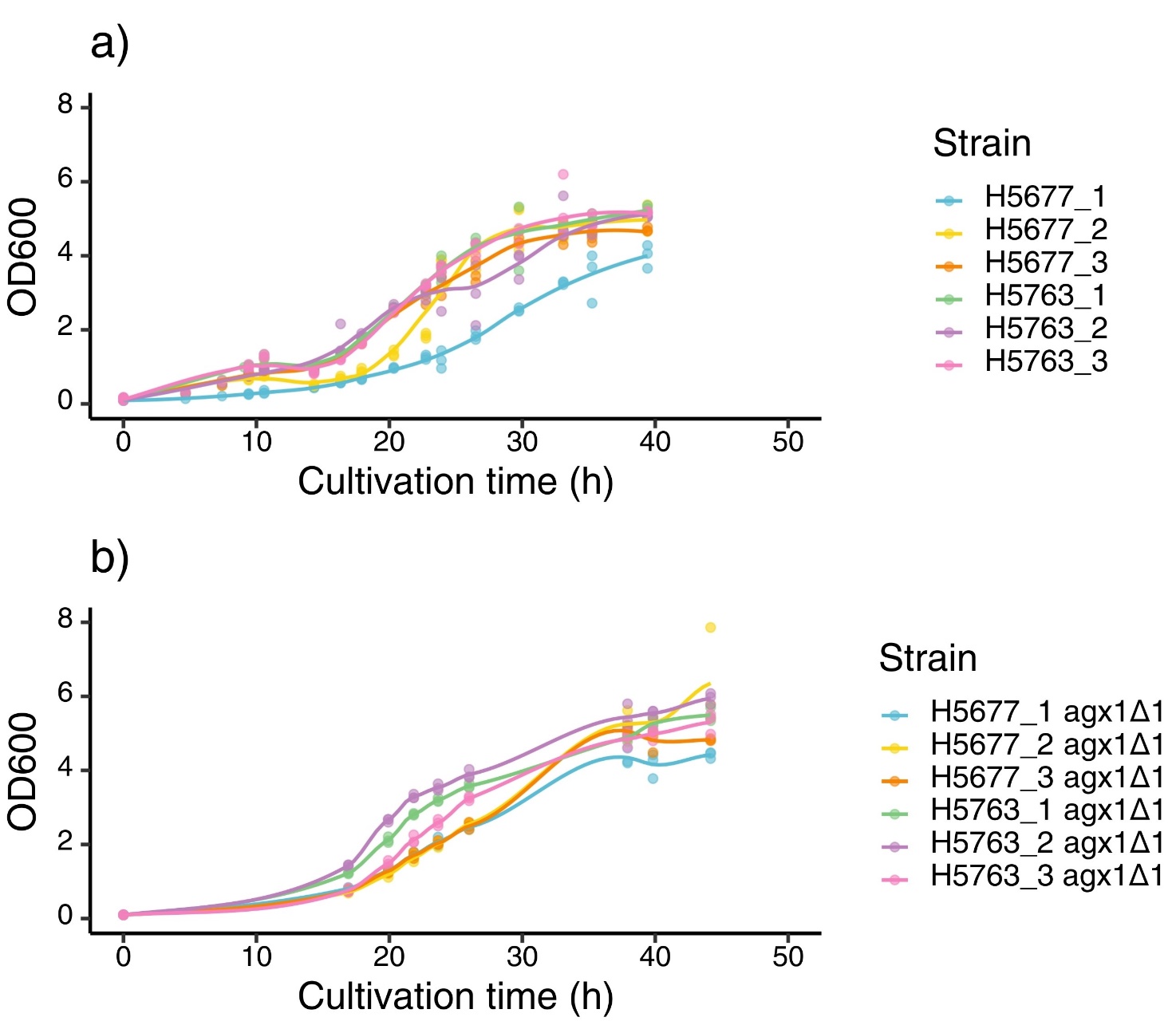


**Supplementary Figure 4.** Growth dynamics as culture turbidity (OD600) as a function of cultivation time of evolved isolates with *GLYR1* integrated, with and without *AGX1* deletion. Full lines are the *loess* fits to average culture turbidities of three or six biological replicates. The growth profiles for lineage H5677_1 are shown in blue, H5677_2 in yellow, H5677_3 in orange, H5763_1 in green, H5763_2 in purple and H5763_3 in pink.


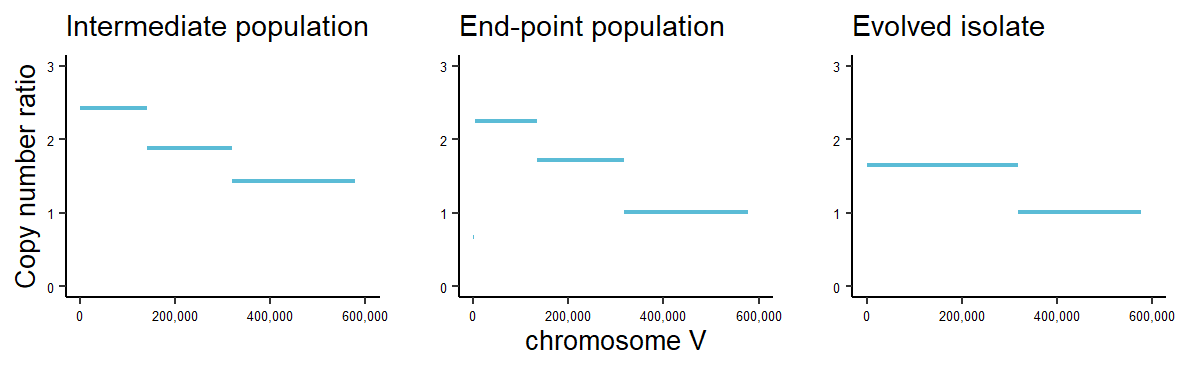


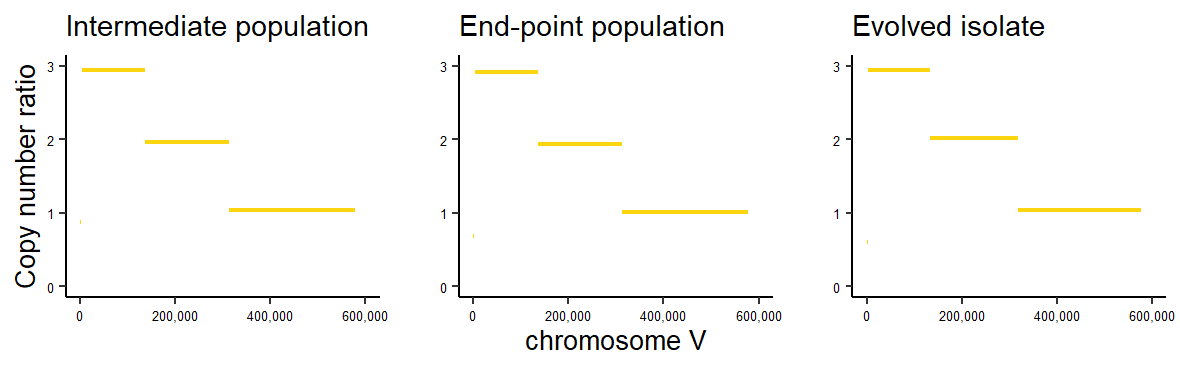


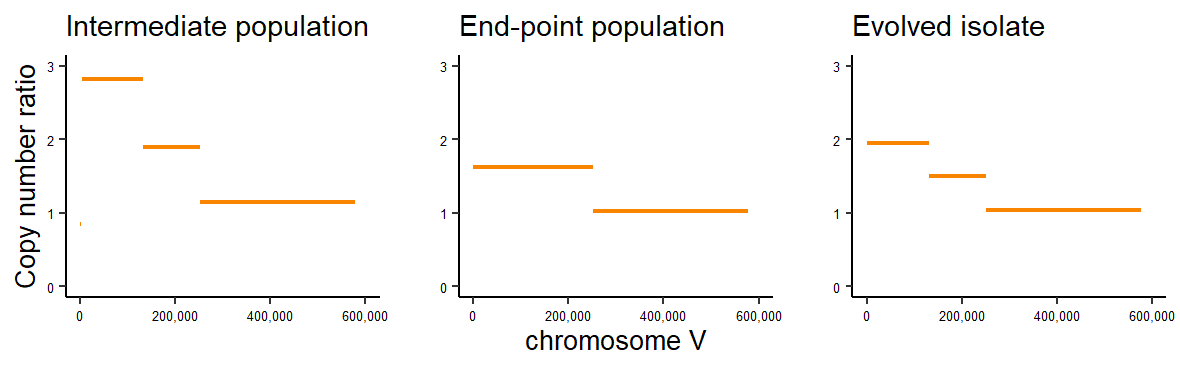


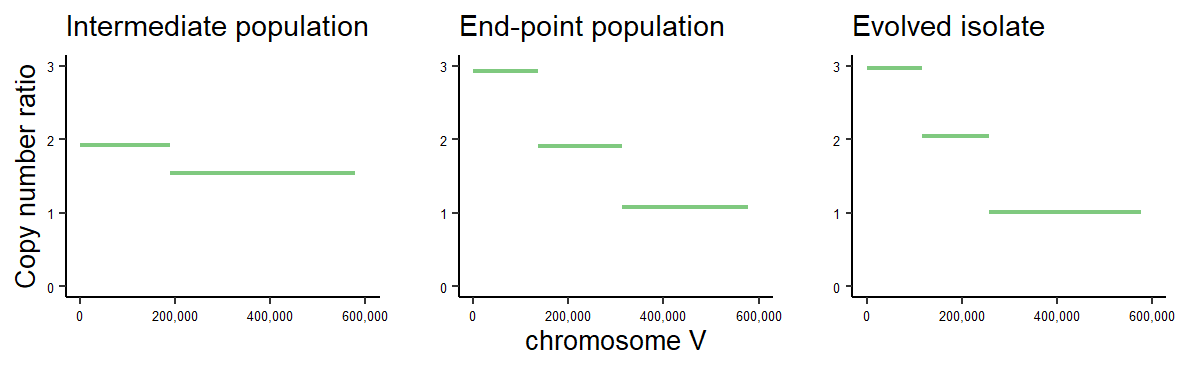


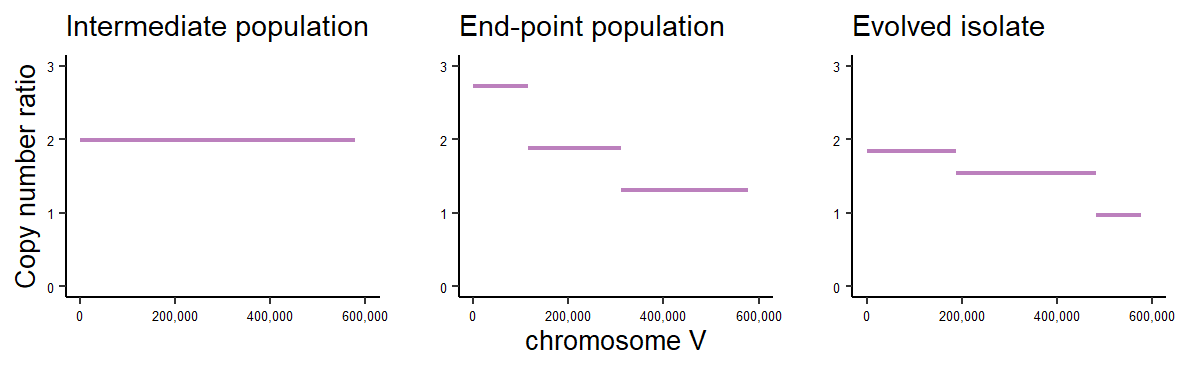


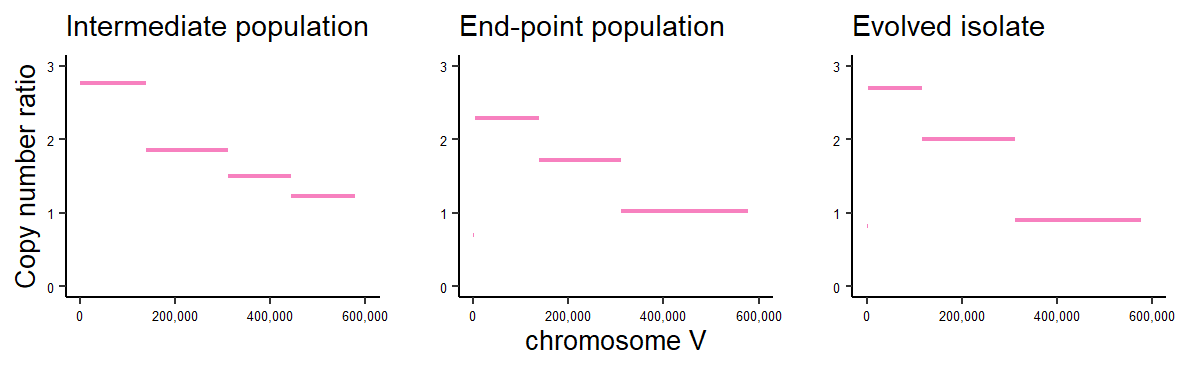


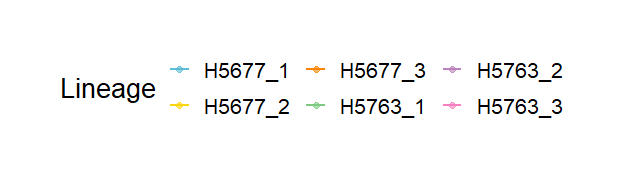


**Supplementary Figure 5.** Copy number variation (CNV) in chromosome V for all six evolution lineages compared to their respective parental clones (H5677 and H5763). The CNVs detected in chromosome V are shown for all evolved lineages at the intermediate population (transfer 10), end-point population (transfer 30) and for the evolved isolate. Lineage H5677_1 is shown in blue, H5677_2 in yellow, H5677_3 in orange, H5763_1 in green, H5763_2 in purple and H5763_3 in pink.


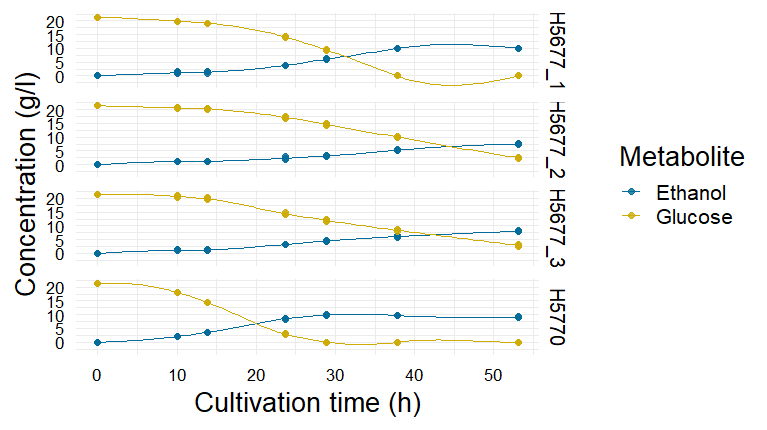


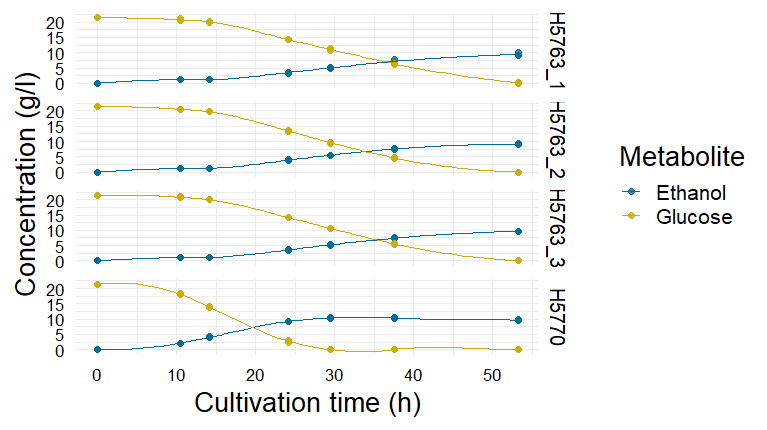


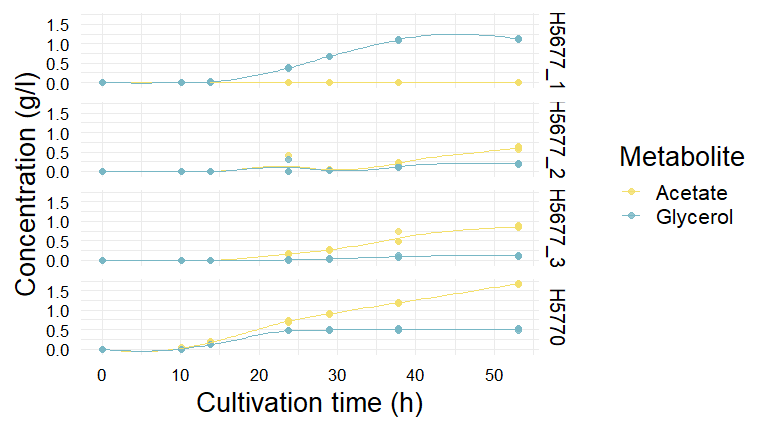


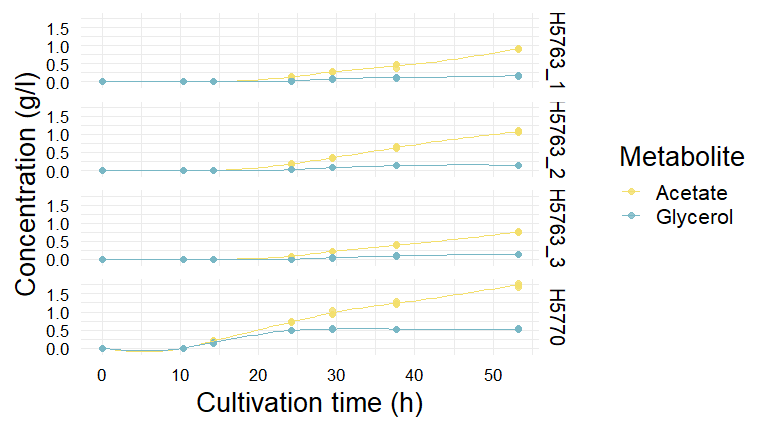


**Supplementary Figure 6.** Major byproduct and secondary metabolite concentrations quantified with HPLC. Extracellular metabolite concentrations over time are shown for glucose in dark yellow, ethanol in dark blue, acetate in pale yellow, and glycerol in pale blue. The curves are shown as *loess* fits to averages of three biological replicates.
